# CROssBAR: comprehensive resource of biomedical relations with knowledge graph representations

**DOI:** 10.1093/nar/gkab543

**Published:** 2021-06-28

**Authors:** Tunca Doğan, Heval Atas, Vishal Joshi, Ahmet Atakan, Ahmet Sureyya Rifaioglu, Esra Nalbat, Andrew Nightingale, Rabie Saidi, Vladimir Volynkin, Hermann Zellner, Rengul Cetin-Atalay, Maria Martin, Volkan Atalay

**Affiliations:** Department of Computer Engineering, Hacettepe University, Ankara 06800, Turkey; Institute of Informatics, Hacettepe University, Ankara 06800, Turkey; Cancer Systems Biology Laboratory, Graduate School of Informatics, METU, Ankara 06800, Turkey; European Molecular Biology Laboratory, European Bioinformatics Institute (EMBL–EBI), Hinxton, Cambridgeshire CB10 1SD, UK; Cancer Systems Biology Laboratory, Graduate School of Informatics, METU, Ankara 06800, Turkey; European Molecular Biology Laboratory, European Bioinformatics Institute (EMBL–EBI), Hinxton, Cambridgeshire CB10 1SD, UK; Department of Computer Engineering, METU, Ankara 06800, Turkey; Department of Computer Engineering, EBYU, Erzincan 24002, Turkey; Department of Computer Engineering, METU, Ankara 06800, Turkey; Department of Computer Engineering, İskenderun Technical University, Hatay 31200, Turkey; Cancer Systems Biology Laboratory, Graduate School of Informatics, METU, Ankara 06800, Turkey; European Molecular Biology Laboratory, European Bioinformatics Institute (EMBL–EBI), Hinxton, Cambridgeshire CB10 1SD, UK; European Molecular Biology Laboratory, European Bioinformatics Institute (EMBL–EBI), Hinxton, Cambridgeshire CB10 1SD, UK; European Molecular Biology Laboratory, European Bioinformatics Institute (EMBL–EBI), Hinxton, Cambridgeshire CB10 1SD, UK; European Molecular Biology Laboratory, European Bioinformatics Institute (EMBL–EBI), Hinxton, Cambridgeshire CB10 1SD, UK; Cancer Systems Biology Laboratory, Graduate School of Informatics, METU, Ankara 06800, Turkey; Section of Pulmonary and Critical Care Medicine, University of Chicago, Chicago, IL 60637, USA; European Molecular Biology Laboratory, European Bioinformatics Institute (EMBL–EBI), Hinxton, Cambridgeshire CB10 1SD, UK; Department of Computer Engineering, METU, Ankara 06800, Turkey

## Abstract

Systemic analysis of available large-scale biological/biomedical data is critical for studying biological mechanisms, and developing novel and effective treatment approaches against diseases. However, different layers of the available data are produced using different technologies and scattered across individual computational resources without any explicit connections to each other, which hinders extensive and integrative multi-omics-based analysis. We aimed to address this issue by developing a new data integration/representation methodology and its application by constructing a biological data resource. CROssBAR is a comprehensive system that integrates large-scale biological/biomedical data from various resources and stores them in a NoSQL database. CROssBAR is enriched with the deep-learning-based prediction of relationships between numerous data entries, which is followed by the rigorous analysis of the enriched data to obtain biologically meaningful modules. These complex sets of entities and relationships are displayed to users via easy-to-interpret, interactive knowledge graphs within an open-access service. CROssBAR knowledge graphs incorporate relevant genes-proteins, molecular interactions, pathways, phenotypes, diseases, as well as known/predicted drugs and bioactive compounds, and they are constructed on-the-fly based on simple non-programmatic user queries. These intensely processed heterogeneous networks are expected to aid systems-level research, especially to infer biological mechanisms in relation to genes, proteins, their ligands, and diseases.

## INTRODUCTION

Data driven approaches are rising as strong candidates to aid researchers in proposing effective personalized solutions especially to complex diseases, with their capabilities regarding the comprehensive analysis of available large-scale biological and biomedical data. One critical shortcoming here is related to data connectivity. Different institutions, with their distinct expertise and infrastructure, continuously update and maintain specific parts of the complex biomedical data. For this reason, connections between data-points across different resources are neither well-established nor explicit, even though entities are biologically related and complementary to each other. In addition to the connectivity problem, another issue related to biomedical data is the incompleteness in knowledge space (e.g. little is known about the possible ligands of a target biomolecule, or the phenotypic implications of a newly identified variant). There is a clear requirement for innovative computational approaches to integrate available biomedical big-data and to complete missing information with accurate *in silico* associations.

There are studies, tools and resources that integrate biological data (either from other data sources or by direct curation) and communicate it via textual or visual representations. One of the most commonly used resources in this sense are biological pathway databases such as Reactome (1), KEGG (2) and WikiPathways (3), where the interactions/reactions are communicated via network representations. STRING and STITCH databases are two well-known molecular interaction services, in which protein-protein and protein-chemical interactions are integrated from various resources, including both experimentally proven and electronically predicted data points, and presented to users as pre-computed networks (4,5). GeneMANIA is an online platform for exploring the relationships between genes over network representations generated by utilizing large-scale genomics and proteomics data, for gene prioritization and function prediction (6). Apart from these well-known resources, there are other relevant studies in the literature. One of the earliest applications of the integration of structured biomedical data was about relating semantically same or similar terms from different ontological systems (without providing any visual output) (7,8). Another one of the early applications of heterogeneous biomedical data integration was the BioGraph data mining platform, which is shown to be successfully utilized for disease gene prioritization via random walks on the generated network (9). In the Bio4j project, authors aimed to construct a graph-based platform as an infrastructure for integrating biological data. They stored public data obtained from sources such as UniProt, Gene Ontology and Expasy in independent graphs to be queried via domain specific languages such as Angulillos (10). In project Rephetio, authors systematically integrated biomedical data from various resources and stored them in a graph database to construct the Hetionet resource, with the primary purpose of inferring new drug/compound–disease relations. Hetionet can be browsed by users via database queries in Cypher language (11). With a similar approach, BioGrakn project aimed to construct a biomedical knowledge graph (KG) using the Grakn database infrastructure. Users are required to download and run the system locally, which can be queried via the Graql language (12). Another system named BioGraph integrates gene/protein, function and cancer related miRNA data from various source databases and lets users to query the data using Gremlin query language to produce information on returned entities and simple network-based visualizations (13). In a few studies, authors discussed alternative ways of extracting and relating biomedical data; (i) from the literature (i.e. articles and similar unstructured textual sources) via text mining (14–17), and (ii) from public biological databases providing ontological data via semantic integration (18), with the aim of constructing biomedical knowledge bases or graphs. Two recent studies evaluated and discussed the use of Wikidata, which is a community-driven semantic knowledge base, as data resource and infrastructure for the generation of biomedical knowledge graphs, over use cases and potential applications (19,20). Lately, numerous bio-pharmaceutical companies start investing in biomedical data integration and mining using graph databases and knowledge graph-based representations (21,22).

Apart from the few widely adopted tools and services, many of the studies mentioned above, especially the ones dealing with heterogeneous biological data, suffer from issues that limit their functionality and/or usability. For example, some of them require highly specialized inputs from users, such as complex database queries, to generate the desired output, which may not be easy for researchers with little or no programmatic background. Some others do not provide an easily-interpretable visualization of the output data, which decreases their usability, since a complex corpus of information is usually hard to consume (without further computational analysis) when communicated only via textual or tabular definitions. Additionally, some resources are not properly maintained, causing the size and the content of the incorporated data to fall behind. In some cases, the authors just published a large dataset (e.g. a graph) for users to download, without any means of a relevant interactive sub-set generation, based on user queries. Finally, considering the proprietary resources belonging to pharmaceutical/bio-development companies, which are claimed to fulfil most of the current biomedical data integration and representation requirements, these tools and services are only utilized internally (in the course of their drug discovery and development projects), not open to public research. Our literature review revealed that there is a critical requirement for fully open access, continuously updated and online biomedical data integration and representation tools/services with coding-free user interfaces, combined with cutting-edge artificial intelligence-based data enrichment applications, to be freely and easily used by the life-sciences research community.

**Figure 1.**
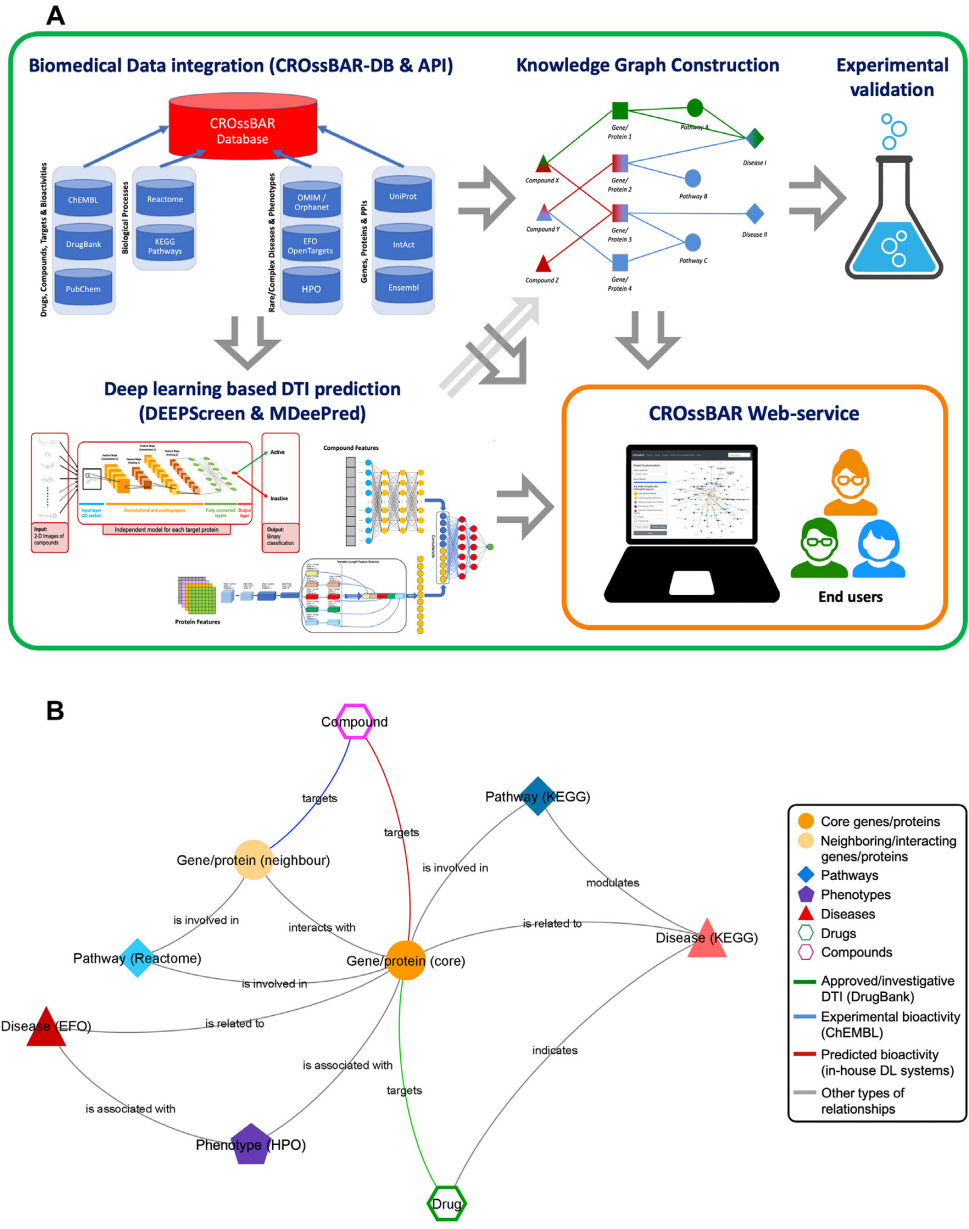
(**A**) Overall schematic representation of the CROssBAR system within five main pillars; (i) large-scale biological and biomedical data integration, (ii) deep-learning based prediction of missing relations, (iii) construction of knowledge graph representations with serial association and filtering operations, (iv) experimental validation of the computational results and (v) open-access web-service with an easy-to-use interface and rich visualization options and (**B**) different types of biological/biomedical components and relationships, and their visual representation in CROssBAR knowledge graphs as nodes and edges.

In this study, we aimed to address these shortcomings by developing a computational method for integrating, representing and visualizing heterogeneous biological data, together with the application of this method as a comprehensive open access system entitled CROssBAR. For this, we first integrated various well-known biological data sources and established a new database. Second, we enriched the data by inferring the missing relations between existing data points via building and applying machine learning models. Third, we constructed informative knowledge graphs based on specific user queries of single or multiple biomedical components/terms such as a gene, protein, disease, phenotype, pathway, drug and/or compound. Schematic representation of the study is given in Figure 1A. CROssBAR is available as a web-service/tool at https://crossbar.kansil.org.

In the following sections, we explain the methodological design of CROssBAR and present the results of its application with a use case, the coronavirus disease 2019 (COVID-19) knowledge graph, and two data exploration examples. We also conducted an *in vitro* study in terms of measuring the changes in gene expression of Chloroquine Phosphate (CQ) treated liver cells and comparing the results with COVID-19 KGs. Finally, we discussed the diversity and stability of the content of CROssBAR knowledge graphs over data-centric analyses.

## MATERIAL AND METHODS

### CROssBAR database and API

#### Integrated data resources and the CROssBAR-DB

We developed extract-transform-load (ETL) pipelines in Java 8 using the Spring batch framework to structure the jobs in CROssBAR-DB. These jobs are executed on state-of-the-art EMBL-EBI LSF clusters in a parallel distributed fashion to reduce the processing time. The data are finally stored in MongoDB in the form of independent data collections, thus, providing schemaless flexibility and faster development, while sustaining data relationships in the form of nested documents. The pipelines have been both unit and integration tested using Spock framework in Groovy language.

The public databases integrated in the CROssBAR system can be listed along with the type of the biological/biomedical data they contain as follows:

- UniProt Knowledgebase (protein sequence and annotations including functions, domains, families, interactions, disease relations, pathway memberships, and more),
- IntAct (protein-protein interactions),
- InterPro (protein domain and family information),
- DrugBank (approved and investigational drugs and their targets),
- ChEMBL (small molecule compounds, targets, bioassays and bioactivities collected from literature and other sources),
- PubChem (small molecule compounds, targets, bioassays and bioactivities collected from various resources),
- Reactome (pathway entries and their relations to proteins, currently incorporated directly from UniProt),
- KEGG (pathway and disease entries together with their relations to genes, currently not direct part of CROssBAR-DB),
- Experimental Factor Ontology – EFO (disease terms integrated from multiple disease-centric databases including OMIM and Orphanet, organized under an ontological system) and
- Human Phenotype Ontology – HPO (phenotypic abnormality terms that relate to both genes and disease entries).

**Figure 2.**
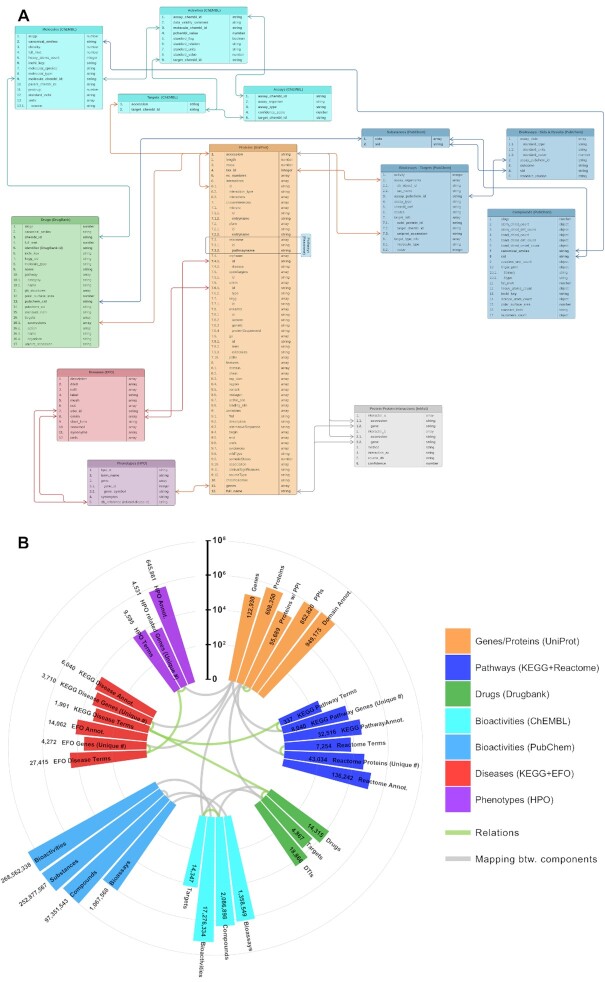
CROssBAR database; (**A**) schema-like representation of the independent collections of CROssBAR Mongo NoSQL database, displaying cross-collection relations; (**B**) CROssBAR database statistics displayed in a circular bar-graph layout, bar lengths are shown in logarithmic scale, each high-level biomedical data component group is displayed with a different colour, grey curves show the matching components in different database collections (i.e. bars connected with grey curves signify the same types of biomedical data -keys-, these mappings are utilized for relating independent database collections to each other), green curves signify the biomedical relationships (e.g. drug–target protein interactions) between different CROssBAR components, statistics are also provided in Supplementary Table S1 (abbreviations; Dis.: disease, Pat.: pathway, DTIs: drug-target interactions).

These biological/biomedical entity types and their relations are displayed in Figure 1B as nodes and edges of a network. CROssBAR-DB schema-like representation is provided both in Figure 2A and in the GitHub repository of the project (https://github.com/cansyl/CROssBAR), where the attributes/fields belonging to each collection can be observed in full detail. The statistics regarding the number of terms and annotations incorporated into CROssBAR-DB from each resource listed above is given in Figure 2B.

#### Obtaining biomedical data via the CROssBAR-API

CROssBAR data services (CROssBAR-API) are developed in Java 8 using Spring Boot's web module in a RESTful architecture style. The API currently provides 13 endpoints documented using Swagger API which allows endpoints to be tested within the documentation, and gives all information about the expected response schema. The API leverages the CROssBAR-DB hosted in a MongoDB platform to fetch data and filter results for users. The web services have been both unit and integration tested using Spock framework in Groovy language. CROssBAR-API is publicly available at https://www.ebi.ac.uk/Tools/crossbar/swagger-ui.html, where users can query independent collections over the indexed attributes/fields. Screenshots of the Swagger API are given in the Supplementary Figure S2, where 13 CROssBAR-DB collections are shown. Here, the collections entitled ‘Activities’, ‘Assays’, ‘Molecules’ and ‘Targets’ correspond to bioactivity, bioassay, compound and target entries in the ChEMBL database, respectively. ‘Drugs’ corresponds to drug entries in DrugBank, ‘EFO disease terms’ corresponds to the disease entries in the Experimental Factor Ontology, ‘HPO’ corresponds to phenotype entries in the Human Phenotype Ontology, ‘Proteins’ correspond to a subset of the protein entries in the UniProtKB, and ‘Intact’ refers to protein-protein interactions that belong to entries in the Proteins collection. The remaining four collections belong to the PubChem data. There is no one-to-one correspondence between the incorporated data resources and the CROssBAR-DB collections since some of the resources had to be split to multiple collections for easier query (e.g. ChEMBL and PubChem). Also, some of the sources are directly incorporated from the UniProt database, thus, reside in the proteins collection (e.g. both terms and annotations for InterPro, Reactome, and only annotations for OMIM and Orphanet).

It is possible to obtain cross-collection relational data (i.e. integrated relational data from multiple collections) by writing programmatic queries and submitting them via the API, similar to our application in CROssBAR-WS to construct the knowledge graphs. Currently, CROssBAR knowledge graphs do not include PubChem data due to both elevated computational demand (the sizes of PubChem collections are large) and high redundancy (a large portion of bioactivity data points in PubChem and ChEMBL databases are shared). However, it is possible to query the CROssBAR-DB using the provided API service, to obtain data entries from PubChem database collections.

### Deep-learning-based prediction of bioactivities

#### Bioactivity dataset construction

One critical topic in developing drug/compound-target protein interaction (DTI) prediction models is the source dataset to be used in system training procedures. It is especially critical to construct large-scale DTI datasets to train deep-learning models. To address this issue, we prepared a DTI dataset from the ChEMBL database that is suitable for training machine learning systems, with standardized filtering operations on targets, compounds and bioactivities. The dataset is periodically updated with each ChEMBL database release. We employed this dataset for the training and validation of the deep-learning based DTI prediction models we developed in the framework of the CROssBAR project, and also as the source dataset for drug/compound-target interaction space visualization (these methods are described below). It can also be used for developing new DTI prediction models. The current version of the bioactivity dataset (ChEMBL v27) is available for public use in: https://github.com/cansyl/CROssBAR/blob/master/CROssBAR_DB_API/ChEMBL27_preprocessed_activities_sp_b_pchembl.zip. Details regarding the dataset can be found in our previous article (23).

#### Deep learning base predictor 1 – DEEPScreen

DEEPScreen was the first tool that we utilized to produce DTI predictions to be integrated into CROssBAR. DEEPScreen is a high-performance drug–target interaction predictor that uses deep convolutional neural networks and 2D structural compound representations (i.e. simple images) to predict their activity against intended target proteins. DEEPScreen system is composed of 704 target protein specific prediction models, each independently trained using experimental bioactivity measurements against many drug candidate small molecules, and optimized according to the characteristics of target proteins. The main novelty of DEEPScreen is employing readily available 2D structural representations of compounds at the input level instead of conventional drug/compound descriptors (e.g. molecular fingerprints) that display limited performance. DEEPScreen produces binary predictions, meaning that a compound is either predicted as active or inactive against a target protein. During the development of this method, we also carried out cell-based *in vitro* wet-lab experiments on computationally generated DTI predictions, with the purposes of both validating the accuracy of the prediction models, and gaining biological insight in the framework of health and disease, especially to contribute to the understanding of processes active in liver cancer. DEEPScreen can be used for the fast screening of the chemogenomic space, to provide completely new DTIs that can later be investigated experimentally within the framework of drug discovery and repurposing (23). The source code, datasets and the results of DEEPScreen are available at https://github.com/cansyl/deepscreen.

To enrich the DTI data in CROssBAR, DEEPScreen was employed to scan a considerable portion of the chemogenomic space and predicted >21 million new DTIs between 1.3 million drug candidate compounds in the ChEMBL database and 532 target proteins. A filtered version of these predictions (∼8 million) was incorporated in CROssBAR and displayed to users as part of CROssBAR-KGs. These predictions can directly be downloaded from: https://github.com/cansyl/CROssBAR/blob/master/CROssBAR_DB_API/CROssBAR_DEEPScreen_Largescale_DTI_predictions_ filtered.tsv.zip.

#### Deep learning base predictor 2 – MDeePred

Our second deep-learning based DTI prediction system ‘MDeePred’ adopts the proteochemometric approach, where both the compound and target protein features are employed at the input level to model their interaction, which enables the prediction of binders to under-studied or previously non-targeted proteins (24). In MDeePred, multiple types of protein features such as sequence, structural, evolutionary and physicochemical properties are incorporated within multi-channel 2-D vectors, which is then fed to state-of-the-art pairwise input hybrid deep neural networks, together with molecular fingerprint-based vectors of compounds. MDeePred predicts real-valued drug/compound-target protein interactions, which can be interpreted as comparable response values such as IC50/Kd/Ki/potency (24). The source code and datasets of MDeePred are available at https://github.com/cansyl/MDeePred.

In the framework of this study, we trained two MDeePred prediction models, with the aim of incorporating their DTI predictions to the COVID-19 CROssBAR-KG. One of these models was trained using ChEMBL experimental bioactivity data of orthologous ACE/ACE2 receptors from different organisms (i.e. human, rat, mouse and rabbit) and used for predicting new inhibitor drugs for human ACE2 receptor. The second model was trained using ChEMBL bioactivity data points that belong to 3C-like proteinase sub-unit of replicase polyprotein 1ab of closely related coronavirus strains (i.e. SARS, MERS, Feline and NL63 coronaviruses) and used for predicting new inhibitor drugs for SARS-CoV-2 3C-like proteinase. For both models, only ∼10 000 drug entries in the DrugBank database (the ones with investigational and approved drug status) were used as the query/test set, since the principal requirement for new potential COVID-19 treatments is to be exempt from early drug development procedures (e.g. pre-clinical analyses, phase I clinical trials, …). Five drugs with high predicted affinities (i.e. most of them with predicted IC50 < 2 uM for 3C-like proteinase and IC50 < 100 nM for ACE2) were selected for human ACE2 (i.e. 7-hydroxystaurosporine, eribaxaban, becatecarin, ticagrelor and amcinonide) and for SARS-CoV-2 3C-like proteinase (i.e. diloxanide furoate, quinfamide, phenyl aminosalicylate, netarsudil and amlodipine) and included in the COVID-19 CROssBAR-KG. Both the ChEMBL derived training datasets of these models and the full prediction results are provided in the GitHub repository of the CROssBAR project (https://github.com/cansyl/CROssBAR). The chemogenomic modelling approach used in MDeePred enabled us to provide predictions for these two targets, which would otherwise be impossible due to the unavailability of training data points, as both SARS-CoV-2 3C-like proteinase and human ACE2 protein have insufficient number of experimental bioactivity data points in source databases for conventional ligand-based modeling.

### Construction of knowledge graphs

In CROssBAR, the data is stored in a non-relational database (MongoDB), as separate collections for easy maintenance and fast querying. As a result, the database itself is not a knowledge graph. Instead, biologically relevant small-scale knowledge graphs are constructed on-the-fly, triggered by users’ queries with a single or multiple term(s) such as the names or identifiers of genes/proteins, diseases/phenotypes, compounds/drugs and/or pathways/biological processes of interest.

In CROssBAR knowledge graphs, biological entities are represented as vertices/nodes. Distinct types of nodes are defined for: (i) biomolecules (i.e. genes/proteins), (ii) biological mechanisms (i.e. pathways), pathologies: (iii) diseases and (iv) phenotypes, small molecule ligands for treatment: (v) drugs and (vi) drug candidate compounds. Relations between different types of biological entities are expressed by the edges of the graph. Edge types vary according to defined relations. For a relation between; (i) two genes/proteins, the edge is labelled as ‘interacts with’, (ii) a gene/protein and a disease, the edge label is ‘is related to’, (iii) a drug/compound and a gene/protein, the edge label is ‘targets’, (iv) a gene/protein and a pathway, the edge label is ‘is involved in’, (v) a gene/protein and a phenotype term, the edge label is ‘is associated with’, (vi) a drug and a disease, the edge label is ‘indicates’, (vii) a disease and a pathway, the edge label is ‘modulates’ and (viii) a disease and a phenotype term, the edge label is ‘is associated with’ (Figure 1B).

**Figure 3.**
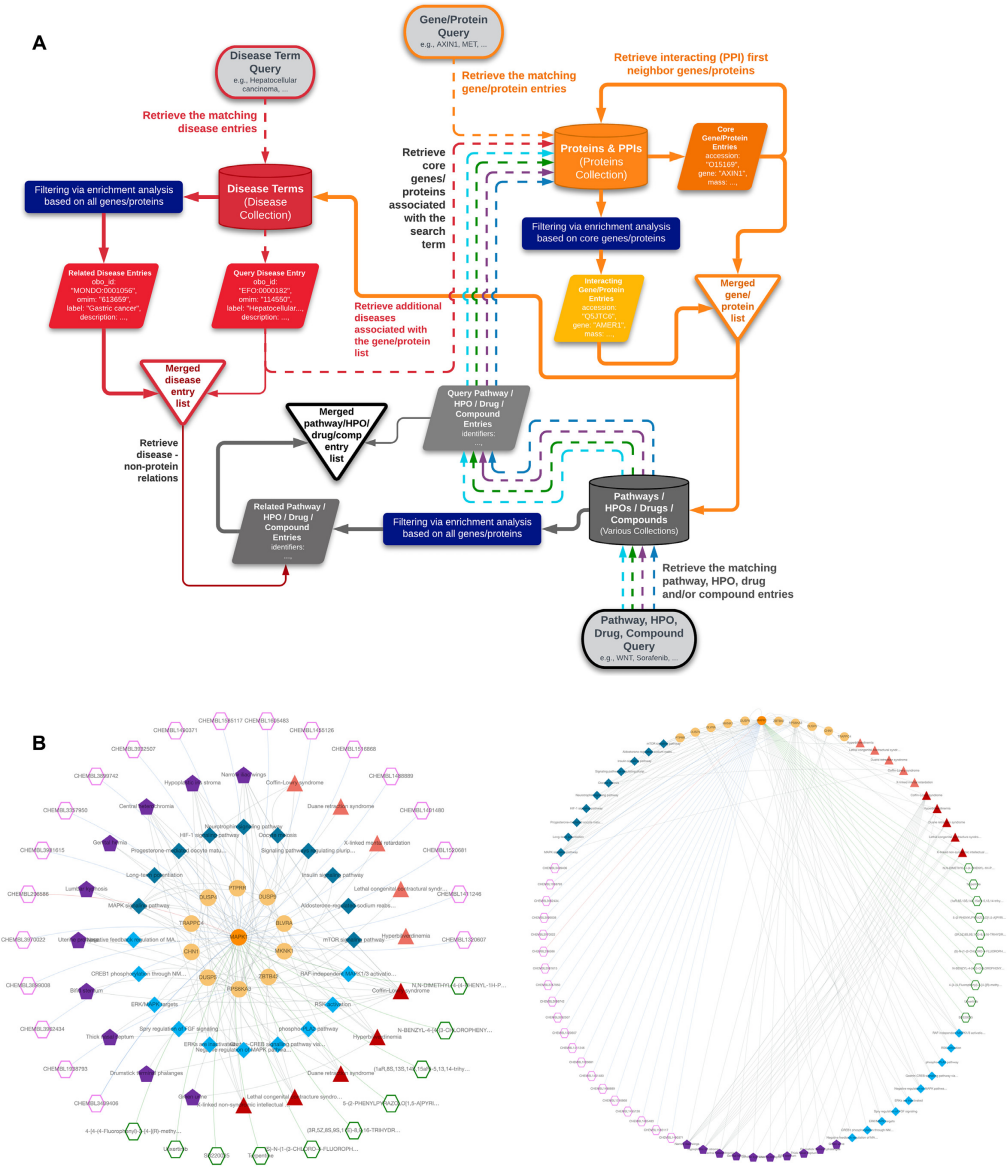
(**A**) Simplified workflow of the knowledge graph construction procedure, explained over an example disease term query. With the initiation of graph construction by a disease query, the system: (1) finds the matching disease entry from the relevant collection, (2) gathers genes/proteins that are associated with the query disease (i.e. core genes/proteins), (3) collects additional genes/proteins (i.e. first-neighbours) using PPIs of core genes/proteins, (4) identifies biological processes (pathways), of which these genes/proteins (core + neighbouring) are members, (5) gathers phenotypic terms (HPO) associated with the whole gene/protein set, (6) obtains known drugs and drug candidate compounds targeting these genes/proteins, together with our deep-learning-based interaction predictions and (7) revisits the disease collection to make another query with all collected genes/proteins, to obtain the disease entries that have similar implications as the query disease. The Full-scale workflow of the CROssBAR knowledge graph construction process is provided in Supplementary Figure S1; (B) an example KG obtained from CROssBAR-WS, generated on-the-fly with the user's query of ‘MAPK1’ gene (with the node limit of 10 for each biomedical/biological component, and other default parameters), displayed under the layout selections of multi-layered CROssBAR (left) and circular (right).

A simplified form of the knowledge graph construction workflow is displayed in Figure 3A. In this figure, parts related to disease and gene/protein collection queries are shown in full detail, and queries on the rest of the components are simplified. The full-scale version of the knowledge graph construction procedure is displayed in Supplementary Figure S1. Here, the finalized filtered dataset of each biological component (i.e. genes/proteins, diseases, phenotypes, drugs, compounds and pathways) is shown with a shape surrounded by a black frame, and the graph is built using entities/terms in these datasets, together with their inter and intra-component relations.

#### Node filtering via overrepresentation analysis

Since the construction of knowledge graphs is based on including all biological components/terms that are associated to the query term(s) directly or indirectly, without further filtering operations, searches would result in huge graphs composed of tens of thousands of nodes and edges. In this case, graphs would be unusable due to multiple reasons. First of all, it would not be possible to visually perceive a biologically relevant result from the giant network. Second, constructing and interactively displaying this graph would have computational requirements so high that it would not be feasible. To address this problem, we applied a multi-staged overrepresentation-based enrichment analysis during the construction of graphs. In this analysis, we calculate an independent enrichment score for each biological entity in the database (i.e. a disease, phenotype, drug, compound, gene/protein or pathway), to be considered as its relevance to the graph that is being constructed. The calculation of enrichment score and its statistical significance is done using a modified version of the hypergeometric test for overrepresentation (25), which also corresponds to one-tailed Fisher's exact test, and it is calculated based on the statistics of relations/connections with gene/protein nodes. For example, the enrichment score (*E_D__,W_*) and its significance (*S_D,W_*) in terms of *P*-value, for a disease term *D*, for graph *W* is calculated as follows:

(1) $$\begin{equation*}{E_{D,W}} = \frac{{{{{m_D^2}} \!\mathord{\left/ {\vphantom {{m_D^2} {{n_W}}}}\right.} \!{{{n_W}}}}}}{{{{{{M_D}}} \!\mathord{\left/ {\vphantom {{{M_D}} N}}\right.} \!{N}}}}\end{equation*}$$

(2) $$\begin{equation*}{S_{D,W}} = \sum\nolimits_{i = {m_D}}^{{n_W}} {\frac{{\left( {\begin{array}{@{}*{1}{c}@{}} {{M_D}}\\ {{m_D}} \end{array}} \right)\left( {\begin{array}{@{}*{1}{c}@{}} {N - {M_D}}\\ {{n_W} - {m_D}} \end{array}} \right)}}{{\left( {\begin{array}{@{}*{1}{c}@{}} N\\ {{n_W}} \end{array}} \right)}}} \end{equation*}$$

where *E_D,W_* is the enrichment score calculated for the disease term *D* for graph *W; m_D_^2^* represent the square of the number of genes/proteins in graph *W* that are associated with disease *D; n_W_* represents the total number of gene/protein nodes in graph *W*; *M_D_* is the total number of genes/proteins (not necessarily in graph *W*) that is associated with disease *D*; and *N* represents the total number of reviewed human gene/protein entries (i.e. UniProtKB/Swiss-Prot entries) in the CROssBAR database that is annotated with any disease entry. *S_D,W_* represents the significance (*P*-value) for the disease term *D* for graph *W* calculated in the hypergeometric test.

Considering the enrichment analysis for diseases, while constructing the graph, an enrichment score is calculated for each disease entry in the CROssBAR database and these scores are used to rank disease entries according to their biological relevance to graph *W* (i.e. in the order of decreasing scores). A cut-off value *k* is employed to include the top *k* relevant disease entries to graph *W*. The default value for *k* is 10, which means that only top-10 relevant diseases will be included. Apart from diseases, the same methodology is used to filter out terms of neighboring genes/proteins, pathways, phenotypes, drugs and compounds. Significance values are not directly used in the filtering operation, since the main objective here is not only including significantly over-represented terms, but just reducing the number of nodes in the graph by filtering out the ones that are least relevant. Significance values are then calculated for the top 100 terms from each biological component and displayed in query reports for users who are interested. In the traditional way of calculating an enrichment score, *m_D_* is without square. The reason behind taking the square of *m_D_* here is mainly to highlight the term with higher *m_D_* value (i.e. a higher degree) in a case of multiple terms with very similar enrichment scores.

#### Formalizing the graph construction around gene/protein entries

During the construction of a knowledge graph, first, the gene/protein entries that are directly connected to the query term (i.e. core proteins) are fetched (e.g. member genes/proteins of a queried signaling pathway). After that, neighboring/interacting genes/proteins are added to the graph by calculating enrichment scores for each interacting protein, using the equation above, and filtering out based on the selected cut-off value. This is followed by the enrichment-based filtering and addition of terms from other biological component types; however, this time, both core and neighboring genes/proteins are taken into consideration to calculate the enrichment scores. If the user starts a heterogenous search that contains multiple terms from different component types, both core and neighboring genes/proteins are independently collected for each non-protein query term, queried gene/protein entries are added to this list (if there is any), and the term collection process is continued using the union of these genes/proteins as the source (Figure 3A). This approach enables the exploration of direct and indirect relations between all query terms.

#### Gene/protein filtering based on source organism(s)

We set a taxonomic filter for the inclusion of gene/protein entries in knowledge graphs, where the default selection is human (tax id: 9606), since the main focus of CROssBAR is biomedicine. Even though there are entries for proteins from hundreds of different organisms in the UniProtKB/Swiss-Prot database, only a few of these non-human protein entries possess annotations in terms of pathway memberships, targeting drugs/compounds and phenotype/disease implications. Thus, many of these protein entries are less useful in terms of constructing biomedical knowledge graphs. Nevertheless, it is possible use the taxonomic filter in the web-service to include genes/proteins from a few additional organisms namely, *Rattus norvegicus* (rat) [10116], *Mus musculus* (mouse) [10090], *Sus scrofa* (pig) [9823], *Bos taurus* (bovine) [9913], *Oryctolagus cuniculus* (rabbit) [9986], *Saccharomyces cerevisiae* (strain ATCC 204508/S288c) (Baker's yeast) [559292], *Mycobacterium tuberculosis* (strain ATCC 25618/H37Rv) (MYCTU) [83332], *Escherichia coli* (strain K12) (ECOLI) [83333], severe acute respiratory syndrome coronavirus (SARS-CoV) [694009] and severe acute respiratory syndrome coronavirus 2 (SARS-CoV-2) [2697049].

#### Bioactive compound and bioactivity selection procedure

Small molecule compounds are selected and incorporated to KGs based on their reported bioactivities against target proteins. In a KG, a compound is represented as a node and a bioactivity is represented as an edge between a compound node and a gene/protein node. We start the bioactive compound collection procedure with a set of target gene/protein entries at hand (gathered in a previous step of the KG construction process), and obtain the compounds that are reported to be bio-actively interacting with these proteins, as their targets. Despite having a simple logic, this procedure is extremely complex due to practical reasons. Since there are more than 15 million bioactivity measurements in the ChEMBL database (v27), we rigorously filter these data points with the aim of providing only the most relevant bioactivity/compound information in CROssBAR-KGs. Since CROssBAR is a gene/protein centric system, we first filter out the data points where the target is not a single protein. We also set an organism filter for the targets, where the default selection is human. Additionally, we filter out bioactivities if their standard (activity) type is not one of these: IC50, EC50, AC50, XC50, Ki, Kd, potency; since these standard types provide roughly comparable measures of half-maximal response. Furthermore, we eliminated data points without a pChEMBL value, which standardizes the above-mentioned standard types under one number in the negative logarithmic scale. Bioactivity data points with an assigned pChEMBL value have usually received additional curation, and thus, they are more reliable. Finally, with the aim of only taking data points at the active binding range (i.e. high affinities between the ligand and the target) we discard the data points with a pChEMBL value <5 (i.e. XC50 > 10 μM). Despite these filtering operations, we still usually end up with tens of thousands of compounds before the compound enrichment analysis, which significantly increases the KG construction run time. Exploiting the fact that it is a better choice to include a compound with higher binding affinity compared to a compound with a lower binding affinity for the same target protein, we set the pChEMBL value cut off value to 8, at the beginning of the compound collection procedure. Then, we reduce the cut off value and re-run the query if the total number of gathered compound entries is less than 1000 in the first run. We iteratively repeat this procedure until we obtain at least 1000 compound entries. Similarly, if the number of returned compounds is >2500 in the first run, we further increase the cut off iteratively until we obtain <2500 compound entries. This number (i.e. 1000–2500) is still much higher than the number of compounds we incorporate to a KG, which is between 0 and 50; however, we aim to enter the enrichment analysis with a high amount to be able to select the compounds that are interacting with multiple proteins in the network, not just one. Another reason is to be able to select diverse compounds, in terms of their scaffolds/structures, which is explained below (under the compound clustering sub-section).

#### Compound clustering

There are more than one million compound entries in the ChEMBL database, most of which have bioactivity data points against target biomolecules. Since it is not feasible to include each and every bioactive compound node in a KG (otherwise the graph would be extremely crowded), only the most overrepresented compounds are tried to be incorporated. We observed that some of the compounds with the same (or a very similar) enrichment score(s) are also structurally very similar to each other. These are mostly molecules with matching scaffolds, which are screened against the same target and produced similar results in the same bioassay. Since their enrichment scores are similar as well, they are either selected or discarded together. To provide a better selection of compounds in the graph, we incorporated a structural property-based filtering in the enrichment analysis. The aim here is to select overrepresented compounds that are as diverse from each other as possible in terms of molecular structures, so that users will be provided with a variety of ligands for the target proteins in the graph. To achieve this, we calculated pairwise molecular similarities between all compounds in the CROssBAR-DB using circular fingerprints (ECFP4) and the Tanimoto coefficient. After that, we clustered the compounds based on a predefined similarity cut-off value of 0.5, meaning that each cluster is composed of compounds that are at least 50% similar to each other. The cluster information is pre-calculated and recorded on our server. Each time a knowledge graph is being constructed, enrichment score ranked compounds are checked one by one in terms of their cluster membership and if there already is a compound from the same cluster in the graph, the compound in turn is discarded (i.e. not incorporated into the graph). The same clustering-based selection approach is applied to incorporate compounds that are computationally predicted to interact with the proteins in the graph.

Following the finalization of the compound nodes, we check whether some of these compounds correspond to drugs that are already incorporated into the KG (since ChEMBL also contains bioactivity measurements belonging to approved or investigational drugs), using the identifier mapping between ChEMBL and DrugBank databases. When a positive case is detected, we merge these two nodes and set the node type as a drug, since drugs are considered more reliable in terms of evidence on their molecular properties and interactions, compared to drug candidate compounds. If there are interactions reported in both DrugBank (as DTIs) and ChEMBL (as bioactivity measurements), we place all of the necessary edges from this drug node to the corresponding target protein nodes in the KG.

#### Evidence-based labelling of compound-target relationships

Relations that signify biophysical interactions between drugs/compounds and target proteins are obtained from three different sources with varying degrees of confidence. The most reliable relation in CROssBAR-DB is obtained from the DrugBank database, where the reported drug–target interaction (DTI) is verified by extensive analyses as part of an official drug development process. The data that came second in terms of reliability are from bioactivity databases such as ChEMBL. In these resources, reported bioactivities are obtained by experimental bioassays; however, they are not as extensively verified as in drug discovery and development procedures. The third in the list of interaction sources is the deep-learning based in-house computational predictions that we produced. These predictions are not verified by any experimental means so they should be considered with caution, even though we carried out numerous computational validation experiments for all predictions and provided *in vitro* experimental verification for a small selected set (23). As a result, predicted relations comprise the least reliable part of the DTI information provided in CROssBAR-KGs. With the aim of providing this evidence-based relation confidence information to users, we used edge labels in KGs. In terms of visualization, these labels are encoded on graphs as colors, such that green color corresponds to DTIs obtained from approved or investigational drugs, blue color corresponds to experimental bioactivity measures obtained from ChEMBL, and the red color corresponds to computationally predicted interactions. During the generation of KGs, if a specific relation is obtained from multiple sources (e.g. when the same relation is reported both in DrugBank and in ChEMBL) the edge label of the more reliable relation is incorporated. To accomplish this task, KG construction process comprises an edge label update procedure.

Another process we applied at this step is the edge addition. Some drugs possess bioactivity data points in ChEMBL in addition to their approved targets in DrugBank. To detect this, we first do a mapping between ChEMBL compound entries and DrugBank drug entries, to find the equivalent ChEMBL entry for each drug. After that, we identify the reported ChEMBL bioactivities between that compound and all of the proteins presented in the KG. We add blue colored edges to represent those relations which were not already incorporated into the KG via DrugBank. The same procedure is applied for adding red colored edges to the drugs and compounds that possess additional computationally predicted target interactions.

### CROssBAR Web-Service, user interface and layout

CROssBAR Web-Service (CROssBAR-WS) comprises both the backend and the frontend processes to construct KGs and to display them to users. CROssBAR-WS utilizes an underlying complex API query set that gathers data from the CROssBAR-DB. The underlying API query collects relevant terms (entries) from 9 independent CROssBAR database collections together with their relations, and it is given in the GitHub repository of the project (https://github.com/cansyl/CROssBAR). CROssBAR-WS also contains a sophisticated graphical user interface that runs on our server. Technologies (all open access) used in the construction of CROssBAR-WS are PHP, JavaScript (cytoscape.js), jQuery, CSS (BootStrap), MySQL.

A user query initiates the term (node) gathering procedure first from the related database collection(s), using the corresponding CROssBAR API(s). Together with the terms that match the search term, the data regarding related/connected terms are obtained from the corresponding collection(s). After that, the next CROssBAR-DB collection is queried with the terms gathered at the previous step. The order of the API queries follows the logic defined for the construction of the KGs, as given in Figure 3A (simplified version), and in Supplementary Figure S1 (full version). Following the initiation of a query, the growing knowledge graph is displayed on the web-browser in real time (using Cytoscape Web), starting from the collection and filtering of core and neighboring genes/proteins. The process is continued with the collection, filtering and addition of phenotype/pathway/disease terms, drugs, bioactive compounds and predicted interacting compounds to the KG (as nodes), together with their relations with gene/protein nodes (as edges). The construction process is finished with the addition of respective edges between non-gene/protein nodes.

**Figure 4.**
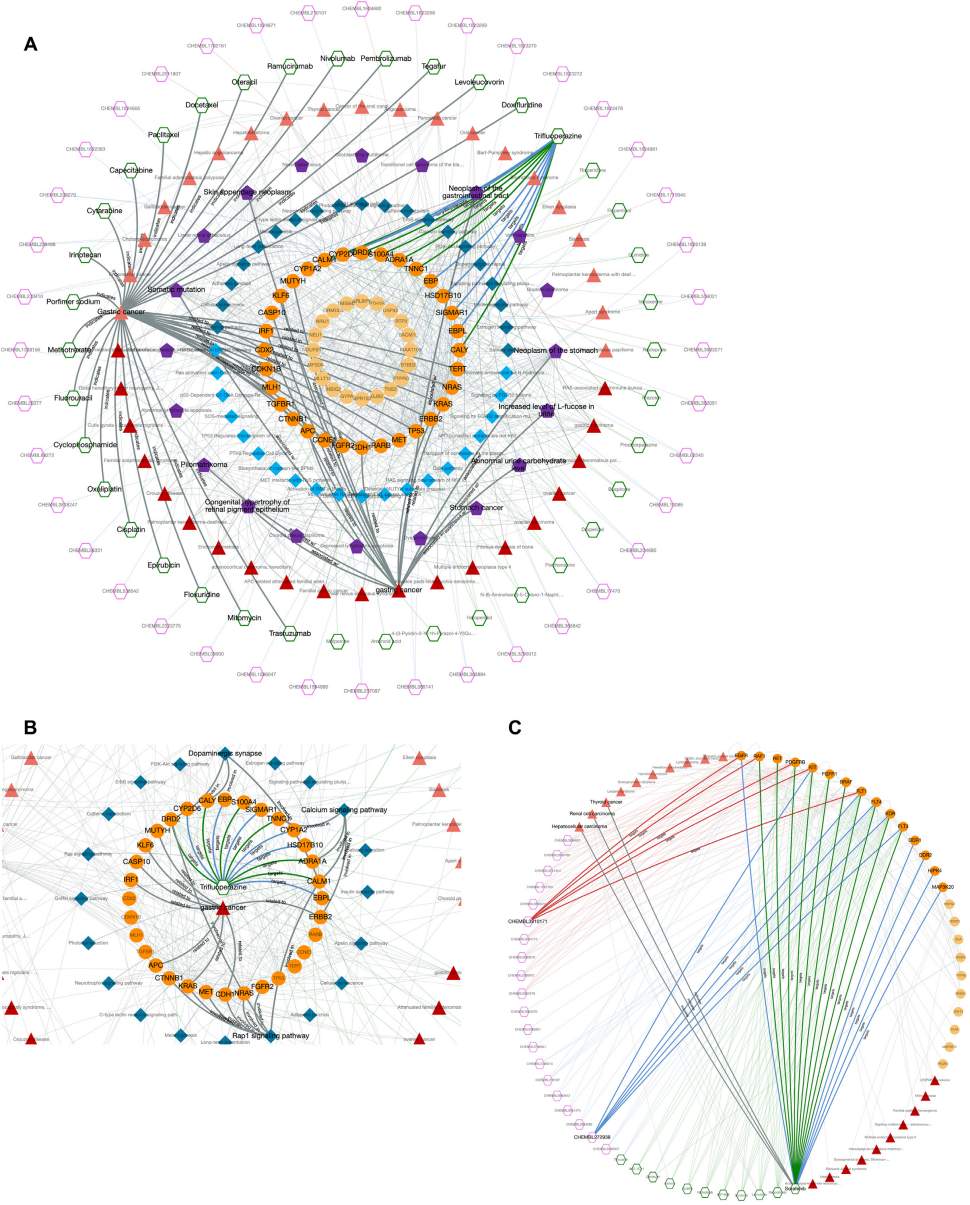
Example cases of data exploration using the CROssBAR web-service; (**A**) the output knowledge graph of trifluoperazine and gastric cancer query; (**B**) critical signalling pathways and their relation to trifluoperazine and gastric cancer over critical genes/proteins and (**C**) target interaction similarity between structurally dissimilar molecules: Sorafenib, CHEMBL272938 and CHEMBL3910171 (circular layout display).

An important topic in graph/network visualization is the layout. In CROssBAR-WS, we incorporated the standard layouts of Cytoscape Web, such as circle, cose, grid and concentric. However, none of these layouts were sufficient for communicating highly heterogeneous graphs with seven different types of nodes and nine different types of edges. To address this problem, we developed the CROssBAR layout, in which biological terms (nodes) from a specific biological/biomedical component (e.g. diseases, pathways, …) are placed on circular points within a fixed radius. There are two versions of the CROssBAR layout, nested layers (default) and isolated layers. In the isolated version, each layer is circularized independently (Supplementary Figure S3), whereas in the nested version, layers are intertwined (Figures 3B and 4A). With the aim of preventing overlapping nodes, the radius of each circle is selected as a different value in the nested version. Both versions come with 4-layer and 7-layer (default) display options, which can be selected by the user solely based on visual preference. Biologically similar components are merged under one layer in the 4-layer display. Curved edge style (i.e. unbundled-bezier) is applied in all layout types to reduce the amount of edge crossing. The output of an example ‘MAPK’ gene query is shown in CROssBAR (nested) and circular layouts in Figure 3B. More information regarding the usage of CROssBAR-WS and its user interface can be found at https://crossbar.kansil.org/tutorial.php.

CROssBAR web-service queries run in linear time and the actual duration of the process is correlated with the total number of core genes/proteins obtained within the query, together with the annotation volume of these genes/proteins. Highly studied genes/proteins usually have high number of associations, which in turn, extends the actual query runtime in practice. According to our tests, most of the queries with disease, gene/protein, drug and compound terms (in terms of both single and combinatory term searches) take 1–3 min to complete (from job submission to the display of the whole graph). However, most pathway and some phenotypic term queries take longer, especially when the number of directly associated genes/proteins is over one hundred. With the aim of creating a better user experience, we applied a procedure in which the collected nodes and their edges are instantaneously and interactively displayed on the screen, before the end of the job. This way, users do not have to wait for the whole job to be finished before starting to explore the KGs. A detailed runtime analysis is provided under the Results section.

The entire CROssBAR web-service including the web-site and the underlying API queries can be found in the GitHub repository of the project (https://github.com/cansyl/CROssBAR).

### Generation of COVID-19 knowledge graphs

Construction of the large-scale COVID-19 graph started with acquiring the related EFO disease term named: ‘COVID-19’ (id: MONDO:0100096). We also incorporated the disease term for ‘Severe acute respiratory syndrome’ (id: EFO:0000694) (the original SARS) into the graph since SARS is better annotated compared to COVID-19. The full-scale COVID-19 KG construction is continued as follows:

#### COVID-19 related genes/proteins and PPIs

We obtained COVID-19 related genes/proteins and their interactions from IntAct database's Coronavirus dataset (downloaded on March 2021). Unlike a genetic disease, human genes/proteins represent only a portion of infectious diseases due to host-pathogen molecular interactions. Therefore, we aimed to incorporate SARS-CoV and SARS-CoV-2 genes/proteins besides the host genes/proteins into the graph. Without any filtering, the KG contained 2951 gene/protein and metabolite nodes from various organisms and 7706 edges. Due to high number of genes/proteins in the KG, there was a risk of incorporating non-specific/irrelevant terms from the other biological components at later steps. To address this risk, we applied several filtering operations on this dataset. First, we eliminated all non-gene/protein nodes and we discarded the genes/proteins if the corresponding organism is not human or SARS-CoV/SARS-CoV-2. Second, we eliminated the protein entries that are not reviewed (i.e. not a member of UniProtKB/Swiss-Prot) except SARS-CoV-2 ORF10 (accession: A0A663DJA2), which currently is an unreviewed protein entry in UniProtKB/TrEMBL (as of March 2021). We also filtered out a portion of the host genes/proteins using interaction-based data using confidence scores reported in IntAct. We discarded the edges between host proteins and SARS-CoV and/or SARS-CoV-2 proteins if the confidence score was <0.35. We also discarded the edges between host proteins in the KG (i.e. neighboring proteins) if their interaction confidence score is <0.6. We removed the disconnected components made up of host proteins, which were formed due to the edge filtering operation. Orthology relations between SARS-CoV and SARS-CoV-2 genes/proteins were annotated with ‘is ortholog of’ edge type. The subunits of large protein complexes such as the NSPs of replicase polyprotein 1ab of SARS-CoV/SARS-CoV-2 were mapped to their corresponding protein complex nodes and excluded from the graph. After these operations, the finalized number of genes/proteins is 778 (746 host genes/proteins, and 15 SARS-CoV and 17 SARS-CoV-2 genes/proteins) and the total number of edges (i.e. PPIs including both virus-human and human-human associations) is 1674. After this point, we started collecting new nodes and edges from various biological components based on the overrepresentation analysis and curation.

#### COVID-19 related drugs and compounds

Approved/investigational drug interactions of COVID-19 related genes/proteins were retrieved from DrugBank database, v5.1.6 release. To incorporate only the most relevant drug-target interactions, a drug overrepresentation analysis was applied with respect to the associations with target genes/proteins in the KG using the hypergeometric distribution, as described in the section entitled ‘Construction of knowledge graphs’. The selected drugs were mapped to their corresponding protein targets via the edge label of green color, as this represents the highest level of confidence in terms of receptor-ligand interactions. DrugBank also has a COVID-19 specific drug list, which includes a curated list of drugs currently under research for COVID-19 treatment. These drugs were included in the KG as well. Drugs without any known targets (or the targets are known but not presented in the KG), were included by connecting directly to the COVID-19 disease node. We also incorporated drug repurposing-based curated and experimental results from critical SARS-CoV-2 related publications such as Gordon *et al.* (26), and we mapped these interactions to our KG with suitable edge labels based on the data source. Finally, we added drug-disease relationships based on reported drug indications obtained from the KEGG resource. The KG contains well-studied drugs for COVID-19 treatment such as Remdesivir (DB14761), Favipiravir (DB12466) and Dexamethasone (DB01234) etc., as well as rather under-studied or non-studied ones (in the context of COVID-19) such as Isosorbide (DB09401) and Rocaglamide (DB15495).

For the retrieval of compound-target interactions based on experimentally measured bioactivities, ChEMBL database (v27) database was utilized. We retrieved the ChEMBL bioactivity data points in binding assays, where the targets are human, SARS-CoV and SARS-CoV-2 proteins, where the pChEMBL value is greater than or equal to 5. Enrichment analysis was applied to select the most relevant ones. Here, only drugs/compounds with enrichment scores greater than 1 and *P*-value less than 0.05 were considered. Compounds were clustered based on Tanimoto coefficient based molecular similarities with a threshold of 0.5, and top 5 overrepresented compound nodes, that are in different clusters, were selected for each target protein (if exist) and incorporated into the KG. We also incorporated selected compound—host target protein and compound—SARS-CoV-2 organism interactions from ChEMBL’s SARS-CoV-2 curated dataset, including both binding and functional assays. Finally, the edge labels are set accordingly (i.e. blue colored edges).

For the computationally predicted drug and compound—target protein interactions, our in-house deep-learning-based tools DEEPScreen (23) and MDeePred (24) were used. DEEPScreen large-scale prediction run results were scanned and 326 bioactive drug/compound-target interaction predictions for 18 human proteins were incorporated to the KG following the application of overrepresentation analysis, similar to the one applied for selecting experimental bioactivities from ChEMBL. We trained two prediction models, one for human ACE2 receptor protein and one for SARS-CoV-2 3C-like proteinase using ChEMBL bioactivity datasets as our training dataset. Both models were used to scan full DrugBank drugs dataset to predict new binders for ACE2 and 3C-like proteinase for *in silico* drug repurposing. The details of this process are given under the Methods sub-section entitled ‘Deep learning-based predictors and dataset construction’. We only incorporated five selected inhibitors for each protein in order to avoid the crowding of the KG, however full-sized prediction sets are provided in the GitHub repository of the project (https://github.com/cansyl/CROssBAR). The selected bioactive drug predictions for ACE2 are Eribaxaban (DB06920), 7-Hydroxystaurosporine (DB01933), Becatecarin (DB06362), Ticagrelor (DB08816) and Amcinonide (DB00288); whereas the predictions for the 3C-like protease are Quinfamide (DB12780), Diloxanide furoate (DB14638), Phenyl aminosalicylate (DB06807), Netarsudil (DB13931) and Amlodipine (DB00381). These predicted interactions are labelled with red colored edges.

We also merged nodes with respect to drug-compound entry correspondences in DrugBank and ChEMBL databases. This way, some of the drug nodes also contain experimental bioassay-based relations (i.e. blue colored edges) and computationally predicted relations (i.e. red colored edges). At the end of these procedures, the total number of drugs (nodes) in the KG is 158 and the total number of drug interactions (edges) is 346. The total number of drug candidate small-molecule compounds in the KG is 167 and the total number of compound interactions (edges) is 664. Out of all drug/compound-target interaction edges, 120 correspond to drug development procedures, 382 to experimental bioassays and 508 to deep-learning-based predictions.

#### Pathways of COVID-19 related host genes/proteins

Signaling and metabolic pathway information was taken from Reactome (via CROssBAR database) and KEGG pathways data sources. The most relevant pathways were determined by overrepresentation analysis and mapped to the related genes/proteins in the KG. Some of the incorporated pathways are directly related to SARS-CoV-2 infection such as ‘Viral mRNA Translation’ (R-HSA-192823), ‘Maturation of replicase proteins’ (R-HSA-9694301) and ‘ISG15 antiviral mechanism’ (R-HSA-1169408), and the others are innate pathways of the host (human) such as ‘Endocytosis’ (hsa04144), ‘Cell cycle’ (hsa04110) or ‘NF-kappa B signaling pathway’ (hsa04064). We also incorporated pathway-disease relations (in the sense of pathways that are modulated due to presence of certain diseases) based on the relationships obtained from the KEGG database. The finalized number of pathways in the KG is 100 (32 for KEGG and 68 for Reactome, among which there are corresponding terms) and the total number of gene/protein-pathway associations (edges) is 1333 (557 for KEGG and 776 for Reactome).

#### COVID-19 related phenotypic implications

The resource for the phenotype terms is the Human Phenotype Ontology (HPO) database. For each phenotype term that is associated with at least one gene in the KG according to HPO data, we calculated an enrichment score and *P*-value via overrepresentation analysis. From the score-ranked HPO term list we selected phenotype terms that are not in a close parent-child relationship with each other in the HPO direct acyclic graph. HPO also has a curated list of SARS related phenotype terms. These terms were also added into the network and mapped to ‘COVID-19’ and ‘Severe acute respiratory syndrome’ disease nodes if their associated genes are not presented in the KG. This way, COVID-19 related phenotypes including symptoms such as Fever (HP:0001945), Myalgia (HP:0003326), Respiratory distress (HP:0002098), Immunodeficiency (HP:0002721) and etc. are included in the graph. The finalized number of phenotype terms in the KG (nodes) is 43 and the number of HPO term - gene/protein associations (edges) is 2427. There are also 56 HPO term - disease associations.

#### Other associated diseases of COVID-19 related host genes/proteins

The aim behind this step is collecting non-infectious (mostly genetic) diseases that utilize the same (or similar) biological mechanisms/processes of human, so that it may indicate potential risks for COVID-19 patients, or potential COVID-19 related repurposing options for drugs that are currently used to treat these diseases. For this, disease terms that are associated with genes/proteins in the COVID-19 KG were collected from the CROssBAR database resources: EFO disease collection (mainly including OMIM and Orphanet disease entries) and KEGG diseases database. The most relevant disease terms were selected based on the results of the overrepresentation analysis. Finally, disease-HPO term relations were also integrated into the KG using the disease association information provided in the HPO resource. At the end of this step, diseases such as Small cell lung cancer (H00013), Amyotrophic lateral sclerosis - ALS (H00058), Bruck syndrome (Orphanet:2771), Osteosarcoma (EFO:0000637), and etc. have entered the KG. The finalized number of disease terms in the KG is 41 (19 for KEGG and 22 for EFO) and the number of disease - gene/protein associations (edges) is 120 (67 for KEGG and 53 for EFO).

The finalized large-scale COVID-19 KG includes 1289 nodes (i.e. genes/proteins, drugs/compounds, pathways, diseases/phenotypes) and 6743 edges (i.e. various types of relations).

For the construction of the simplified COVID-19 KG, the starting point was the COVID-19 associated proteins in the UniProt COVID-19 portal (https://covid-19.uniprot.org/), instead of the IntAct Coronavirus dataset. The remaining steps of building the graph were mainly similar except that, additional nodes representing the organisms: human, SARS-CoV and SARS-CoV-2 were placed in the graph and connected to the corresponding proteins. The aim here was to prevent the presence of singleton protein nodes due to the reduced number of included genes/proteins and PPIs in the simplified graph. It is also important to note that the simplified version is not just a subset of the large-scale KG. Since the starting point of gene/protein collection were different between two KGs, the resulting graphs have slightly different contents as well. For example, the drugs Siltuximab (DB09036) and Pirfenidone (DB04951) are specific to the simplified KG. The simplified COVID-19 KG includes a total of 435 nodes and 1061 edges. The detailed statistics for both KGs are provided in Supplementary Table S5.

For the Cytoscape network files of both COVID-19 KGs, overrepresentation analysis results, and for more information about the CROssBAR COVID-19 KGs please visit the CROssBAR project GitHub repository at: https://github.com/cansyl/CROssBAR. For directly visualizing and exploring the COVID-19 KGs interactively, please visit CROssBAR web-service at: https://crossbar.kansil.org/covid_main.php.

### *In vitro* experimental procedures for chloroquine treatment on liver cells

#### Cell culture

Normal hepatocyte-like epithelial Huh7 cells and mesenchymal-like Mahlavu liver cells were grown in Dulbecco's modified Eagle's medium (DMEM) supplemented with 10% fetal bovine serum (FBS) (Gibco/Thermo Fisher Scientific), 0.1mM non-essential amino acids (Gibco/Thermo Fisher Scientific), and 100 units/ml Penicillin/Streptomycin. Cells were maintained in 37°C in a humidified incubator under %5 CO_2_.

#### NCI-60 sulforhodamine B(SRB) cytotoxicity assay

Huh7 and Mahlavu liver cells were grown in 96-well plates (1000–200 cells/well) in an incubator for 24 h. Both Mahlavu and Huh7 cells were treated with Chloroquine Phosphate (CQ) and water control in 40 μM to 0.3 μM concentrations for 72 h. After fixation with cold 10% (w/v) trichloroacetic acid (MERCK) for an hour at +4°C, plate wells were washed three times with ddH_2_O. Each well was stained with 50 μl of 0.4%SRB dye(Sigma-Aldrich) and incubated at RT for 10 min. To remove unbound SRB dye, wells were washed with 1% acetic acid for four times and left to air-dying. The protein-bound SRB was solubilized in 100 μl/well 10 mM Tris-base solution, and the absorbance was measured with 96-well plate reader at 515 nm wavelength (ELx800, BioTek).

#### Gene expression analysis of chloroquine with NanoString multiplex gene expression panel

Huh7 and Mahlavu liver cells were treated with CQ at cytotoxic doses of 3.6 μM and 12 μM, respectively, for 48 h. NanoString nCounter multiplex gene expression analysis, which includes 770 genes and various canonical pathways such as PI3K, MAPK, STAT, RAS, Cell cycle, DNA damage control, apoptosis, Hedgehog, Wnt, Transcriptional regulation, chromatin modification, and TGF-β, was applied on the RNA extracted from cells using the RNeasy mini kit (Qiagen), followed by the hybridization with code sets and scanning using the nCounter Digital Analyzer as instructed by the manufacturer (NanoString Technologies). Results were analyzed using the Advanced Analysis Module on nSolver™ 3.0 software for quality control, normalization, and differential expression. The expression levels of each gene were normalized to those of control genes. After obtaining the differentially expressed gene list with the native software, a further filtering operation was applied based on the *P*-value (i.e. <0.01), to identify the finalized list of genes with statistically significant expression changes. Differential expression of key pathways was revealed for both Huh7 and Mahlavu cell lines using the fold change of the member genes and their significance values. The resulting files are provided in the GitHub repository of the project (https://github.com/cansyl/CROssBAR).

## RESULTS

CROssBAR is composed of five sub-projects: (i) the construction of the CROssBAR database and its API service to house and serve the integrated biomedical data, (ii) training deep-learning based drug/compound-target protein interaction (DTI) prediction models and their application to identify previously unknown ligands for target proteins, (iii) network based organization and analysis of large-scale biomedical data using heterogeneous knowledge graph representations, (iv) *in vitro* wet-lab experiments at different levels of the project in order to validate/assess the relevance of *in silico* generated knowledge and (v) the establishment of an open access web-service, where user defined biomedical term queries are processed via on-the-fly generation of knowledge graphs with both tabular and network-based visualization and download options. CROssBAR system is schematically represented in Figure 1A. The results and outputs of these sub-projects are summarized below, also, technical/methodological information is provided for each one in the Methods section.

### Constituents of the CROssBAR system

#### Biological data integration

CROssBAR database (CROssBAR-DB) comprises selected features from multiple data sources namely UniProt, IntAct, InterPro, Reactome, Ensembl, DrugBank, ChEMBL, PubChem, KEGG, OMIM, Orphanet, Gene Ontology, Experimental Factor Ontology (EFO) and Human Phenotype Ontology (HPO). Extract-Transform-Load (ETL) pipelines were developed for heavy lifting of data from these resources by persisting specific data attributes with the implementation of logic rules. These pipelines fetch, cleanse, validate and consolidate the data, and thus, implement a multi-omics data integration approach to release a single resource based on MongoDB collections. CROssBAR-DB, which provides a broad spectrum of information such as biological functions, domains, interactions, pathways, diseases, phenotypes, drugs, compounds, and their associations with biomolecules, is hosted and maintained by the EMBL-EBI. The database schema-like representation and current data statistics of the CROssBAR-DB are shown in Figure 2A and B, respectively. CROssBAR-DB is periodically updated on demand/request basis via an automated procedure, which makes the underlying data up to date most of the time. CROssBAR-DB can be queried via a public RESTful API at: www.ebi.ac.uk/Tools/crossbar/swagger-ui.html, which provides a multi-faceted view of the stored data through 13 endpoints (Supplementary Figure S2). The professional service providing approach applied in CROssBAR allows proper and constant maintenance of both the database and its APIs.

#### Deep learning-based prediction of relationships

The identification of novel drug-like compounds and discovering new usages of existing drugs are critical for drug discovery and development. Traditionally, this is accomplished via costly and time-consuming procedures and the rate of identifying novel drugs has decreased in recent years.

Out of all different biomedical entity relation types, drug/compound-target protein interaction is one of those with the highest rate of data incompleteness considering the current knowledge. There are >100 million distinct drug candidate compound records in public bioactive chemical databases such as ChEMBL and PubChem, let alone the theoretical number of all possible small molecules around 10^60^. Considering their pairwise combinations against hundreds of thousands of target biomolecules such as single proteins and macromolecular complexes, the current knowledge corresponds to <0.001% of the whole compound-target space (27). The high rate of missing DTI data negatively impacts the development of new computational methods in the field of drug discovery and development as well.

To address this issue, we previously developed two novel deep-learning-based predictive systems: DEEPScreen (*23*) and MDeePred (*24*) as a part of the CROssBAR system, to enrich the available bioactivity data by identifying unknown interactions between drugs/drug-candidate compounds and target proteins. DEEPScreen employs convolutional neural networks to process 2D structural images of drugs/compounds in 704 individually optimized high performance target-based prediction models, suited for well-studied targets (*23*). MDeePred utilizes both compound and target protein features within a pairwise input hybrid deep neural network architecture to produce real valued bioactivity predictions, especially for targets with a few training instances (*24*). We showed that, both of these methods perform better than the state-of-the-art DTI predictors (in terms of prediction performance) on multiple hold-out test datasets, furthermore, we validated them with additional *in silico* and *in vitro* analyses (23,24). In the framework of this study, we trained both systems using carefully filtered and integrated data in CROssBAR-DB, and ran our trained-models on large compound and human protein spaces to obtain comprehensive bio-interaction predictions, which are included in our knowledge graphs. We also developed an accompanying computational tool, iBioProVis, which is an unsupervised-learning-based visualization system for exploring large drug/compound-target interaction datasets in reduced dimensions (28).

#### Knowledge graph representations

The term knowledge graph (KG) defines a specialized data representation structure, in which collections of entities (nodes) are linked to each other (edges) in a semantic context (29). In this study, we chose to represent heterogeneous biomedical data using a KG-based structure. In CROssBAR knowledge graphs (CROssBAR-KG), biological components/terms (i.e. drugs, compounds, genes/proteins, bio-processes/pathways, phenotypes and diseases) are represented as nodes, and their known or predicted pairwise relationships are annotated as edges (a protein and its coding gene is treated as one merged term/entry/node). The logic behind the construction of a knowledge graph is centered around queried biological components/terms, as shown in Figure 3A with a simplified workflow diagram and an example disease term query. At each step of the KG construction process, an overrepresentation-based enrichment analysis has been performed to select the terms that are significantly associated with the growing graph, and to discard the rest. This analysis comprises a series of hypergeometric tests, based on the recorded relations in the CROssBAR database. Here, we applied a layered construction approach, always taking the genes/proteins at the centre of the enrichment analysis. Finally, additional relationships are incorporated to the graph as edges between existing nodes (e.g. drug–disease, disease–pathway and disease–HPO), to further enrich the provided relational information. During the construction of graphs, terms (nodes) and their pairwise relationships (edges) are obtained from the CROssBAR-DB. CROssBAR-KGs reveal the direct and indirect relationships between all of the terms in the graph. These intensely-processed heterogeneous biological networks are expected to aid biomedical research, especially to infer mechanisms of diseases in relation to biomolecules, systems and candidate drugs.

#### Open-access web-service

We developed the CROssBAR web-service (CROssBAR-WS) to make CROssBAR-KGs available to the public in an easily interpretable and interactive way (https://crossbar.kansil.org). KGs are presented visually on web-browsers as flexible Cytoscape (30) networks. Users can create simple queries by typing names or identifiers of biomedical terms, individually or in combination, to obtain a relevant graph of molecular relationships. Combinatory term query is especially critical as it provides the ability to investigate the indirect biological relationships between the terms from both the same and different biomedical components. Since there are billions of different ways to query CROssBAR, it was not feasible to pre-calculate the resulting graphs; therefore, they are set to be constructed on-the-fly, in real-time. Several options are provided to users to customize the procedure both before and after the search, such as the UniProt databases to be used (UniProtKB/Swiss-Prot or UniProtKB/Swiss-Prot + UniProtKB/TrEMBL), taxon(s) to be selected, inclusion/exclusion of experimental bioactivities and *in silico* DTI predictions, and the number of terms/nodes to include from each component type (which are selected based on enrichment scores). It is also possible to display the resulting graph using a variety of layout options, including our in-house CROssBAR-layout with isolated (Supplementary Figure S3) or nested (Figure 4A) layer versions and 4- or 7-layer display choices. The interactive visualization also lets users prepare a custom display by relocating the nodes/edges as desired. Saving options let users to store the graph in different formats, including json, figure-ready snapshots and protein-centric delimited data-tables. Finally, at the end of each query, a report is provided with the names, identifiers, enrichment scores and *P*-values of the terms (including the ones that were not incorporated into the graph due to low enrichment scores) are provided.

### Biomedical data exploration via CROssBAR

It is possible to query CROssBAR with a combination of terms which allows the user to investigate relationships between multiple entities of interest from the same or different biological/biomedical component(s). This type of query may be composed of any number and combination of components (displayed in Figure 1B as nodes), and the output knowledge graph will contain relationships of different semantic interpretations (shown in Figure 1B as edges) directly or indirectly. Combinatory term queries work with the same graph construction logic as single term queries by collecting core genes/proteins and expanding to other components from this core set. Considering a case where the user is investigating a hypothesis involving a few genes/proteins, a signalling pathway, a target disease together with its phenotypic implication(s), one or more drug(s) and/or drug candidate compound(s) that are being evaluated to intervene with the corresponding pathology, the search is to be initiated by entering the terms of interest to the dedicated fields in the CROssBAR-WS interface (https://crossbar.kansil.org). Our automated pipeline starts the KG construction process (as displayed in Figure 3A within a simplified version and in Supplementary Figure S1 in the full-scale) by first collecting the corresponding database entries for all of the query terms, which is followed by the identification and collection of gene/protein entries that are directly associated with these query terms (these association/relationship types shown in Figure 1B), as core genes/proteins. In other words, this core gene/protein set constitutes queried genes/proteins plus genes/protein entries that are directly connected to the queried pathway(s), disease(s), phenotype(s), drug(s) and compound(s). Following that, neighbouring gene/protein entries are collected using PPI data of core genes/proteins. While finalizing neighbouring genes/proteins, an enrichment analysis is applied to select only the most relevant terms that are highly connected to the core genes/proteins and infrequently connected to other gene/protein entries in the database (number of the terms to be included in a KG can be determined by the user). This is followed by the collection of additional pathways, diseases, phenotypes, drugs and compounds that are highly associated with the total gene/protein set (core and neighbouring genes/proteins), using the same enrichment-based filtering logic. Lastly, non-gene/protein edges are identified and added to the graph.

To provide an example on possible ways of using the CROssBAR system, we explored the relation between a drug (trifluoperazine) and a disease (gastric cancer), to make a very quick and rough evaluation about the potential repurposing of this drug towards the disease of interest. Trifluoperazine is an approved antipsychotic agent mainly used in the treatment of schizophrenia (31). As far as we are aware, trifluoperazine has no *in vitro*, *in vivo* or clinical studies concerning the treatment of gastric cancer, although there are studies on other types of cancer such as colorectal (32), pancreatic (33) and lung (31), in the literature. Also, there is a study indicating the inverse association between antipsychotic use and the risk of gastric cancer (34). Thus, this may be a convenient scenario for investigating the relationship between two potentially related biomedical entities/terms, gastric cancer and trifluoperazine. To construct the corresponding knowledge graph, we queried the CROssBAR-WS with these drug and disease entries and selected the number of nodes to be incorporated to the graph (from each biomedical component) as 20. The resulting graph is shown in Figure 4A.

Trifluoperazine exerts its antipsychotic effect with the blockage of dopamine D2 receptor. This relation is shown in the graph, where trifluoperazine binds to the DRD2 gene/protein node and is associated with the dopaminergic synapse pathway. In the KG, it is possible to observe trifluoperazine's other approved targets such as CALM1, ADRA1A and TNNC1 proteins (approved drug-target interaction edges are in green colour), and these proteins are members of the calcium signalling pathway. Moreover, DRD2 and CALM1 are associated with the rap1 signalling pathway. Also, according to the KG, both calcium and rap1 signalling pathways have other gene/protein associations such as ERBB2, KRAS and CDH1, which are further associated with the gastric cancer disease. In the light of these relations, trifluoperazine can be explored via additional *in silico*, *in vitro* / *in vivo* and clinical studies, in terms of its potential to become a repurposed agent for the treatment of gastric cancer, which may show its activity on gastric cancer cells via calcium (35,36) and rap1 signalling pathways (37) (Figure 4B).

Some of the proteins that are associated with the gastric cancer (e.g. KRAS, ERBB2, TP53, etc.) are also related to other cancer disease nodes in the graph such as the pancreatic cancer, ovarian cancer, endometrial cancer and cholangiocarcinoma, which means that trifluoperazine may also have a potential against these cancer types, worthy of further exploration. Other antipsychotic or anxiolytic agents such as risperidone, haloperidol, perphenazine, buspirone, droperidol, and prochlorperazine are enriched in the network as well, which bind to DRD2, CALM1 and/or ADRA1A. These drugs may also become alternative repurposed drugs for gastric cancer treatment or other cancers presented in the KG. In addition to the above-mentioned approved drug-target interactions, the graph also includes enriched drugs and drug like compounds having experimentally measured bioactivities from ChEMBL (shown with blue coloured edges) or DTIs that are computationally predicted by our in-house tool DEEPScreen (shown with red coloured edges) against the targets DRD2, ADRA1A, EBP and SIGMAR1; which can also be considered for the diseases in the graph. Finally, there are several phenotypic implication terms (from HPO) on the KG, such as the abnormal urine carbohydrate level and the congenital hypertrophy of retinal pigment epithelium, which are associated with gastric cancer disease node and/or gastric cancer related genes. These phenotypic implications could also be helpful considering clinical studies.

Apart from the exploration of potential drug repurposing applications, a drug search on CROssBAR can also be utilized towards identifying new drug-like compounds with similar target-based bioactivities. This kind of exploration can be useful for medicinal chemists and other researchers working on drug discovery. It is generally accepted that compounds with highly similar molecular structures also have similar bioactivities; however, there is no generally accepted approach for identifying compounds that can be alternatives to an approved drug, when there is no structural similarity between the drug and the candidate compounds. In this example, we queried Sorafenib (https://www.drugbank.ca/drugs/DB00398), a drug approved for the treatment of primary kidney and primary liver cancers, in CROssBAR-WS to construct its respective knowledge graph (Figure 4C). An interesting observation on the resulting KG are the compound nodes: CHEMBL272938 (https://www.ebi.ac.uk/chembl/compound_report_card/CHEMBL272938/) and CHEMBL3910171 (https://www.ebi.ac.uk/chembl/compound_report_card/CHEMBL3910171/), which contain high number of shared targets with Sorafenib (5 out 10 of the approved target proteins of Sorafenib are also the targets of these compounds) indicated by the bioassay-based interaction data (blue coloured edges on the graph) for CHEMBL272938 and by computationally predicted interactions (red coloured edges on the graph) for CHEMBL3910171. It is also important to note that, the other half of the approved targets of Sorafenib could also be shared with these compounds; however, we currently do not have further experimental information about it. High overlap between the targets may indicate the potential of these compounds to be alternatives for Sorafenib; however, additional computational and experimental analyses are required to comment further. It is also important to note that this result could not be obtained with a conventional molecular similarity search, since Sorafenib, CHEMBL272938 and CHEMBL3910171 have dissimilar structures (neither a substructure search, nor a pairwise molecular similarity search—with the minimum similarity threshold of 40%—on the ChEMBL database could detect any similarity between these three molecules).

### COVID-19/SARS-CoV-2 molecular relationship KGs

**Figure 5.**
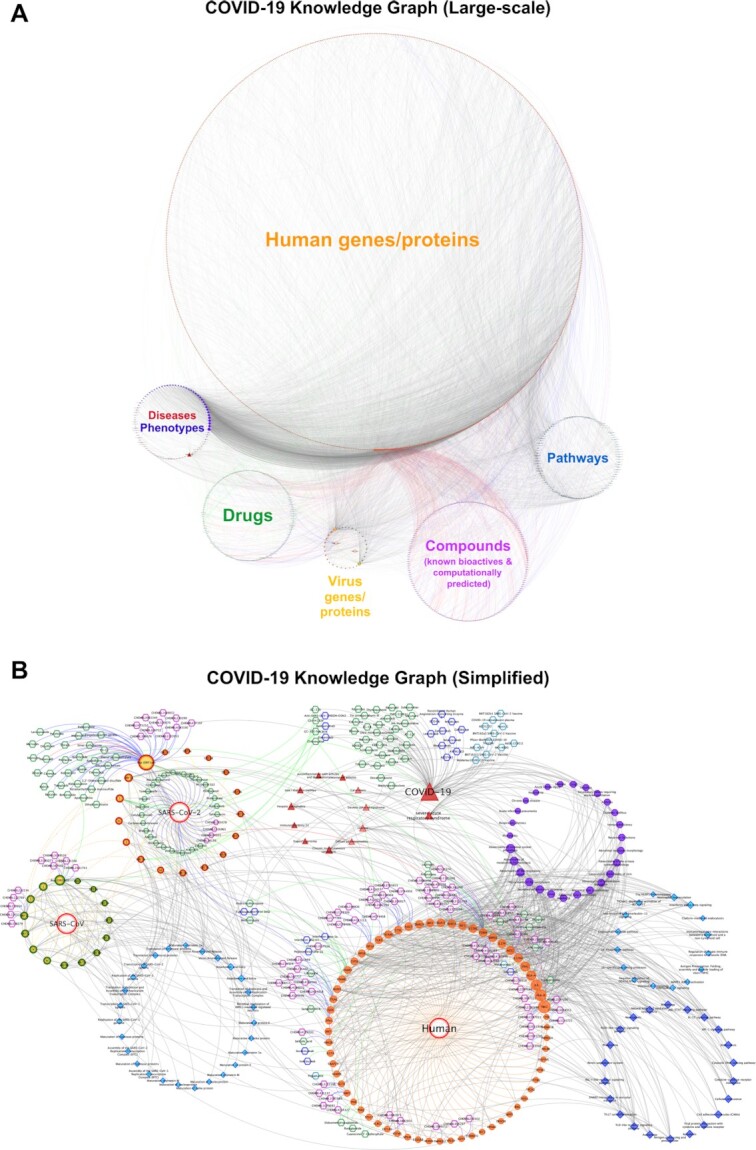
The use case of CROssBAR COVID-19 knowledge graphs (https://crossbar.kansil.org/covid_main.php): (**A**) the large-scale KG (1289 nodes and 6743 edges) and (**B**) the simplified KG (435 nodes and 1061 edges). Both of these graphs reveal the most overrepresented biological processes during a SARS-CoV-2 infection (i.e. cell cycle, viral mRNA translation, endocytosis, interleukin signalling, etc.), as well as, the potential treatment options with COVID-19 related pre-clinical/clinical results (e.g. Remdesivir, Favipiravir, Dexamethasone, etc.) and our novel *in silico* predictions (for both virus and host proteins) considering long-term drug discovery or short-term drug repositioning applications (e.g. tocilizumab, cyclosporine, becatecarin, tenecteplase, simvastatin, etc.). It also displays rare and complex diseases and phenotypic implications with similar host protein associations (e.g. arthritis, diabetes, respiratory distress, fever, etc.).

As a use case of the system, we present Coronavirus disease 2019 (COVID-19) CROssBAR-KGs (https://crossbar.kansil.org/covid_main.php). Starting from the end of 2019, the new coronavirus (SARS-CoV-2) pandemic has ravaged the entire globe and caused immeasurable damage (38). As of March 2021, the scientific endeavour to develop effective drugs is still at peak, and systemic evaluation of the current knowledge about SARS-CoV-2 infection is expected aid researchers in this struggle. To demonstrate the capabilities of CROssBAR, we have constructed two different versions of the COVID-19 knowledge graph, (i) a large-scale version including nearly the entirety of the related information on different CROssBAR-integrated data sources, which is ideal for further network and/or machine learning based analysis or a detailed inspection (Figure 5A) and (ii) a simplified version distilled to include only the most relevant genes/proteins as provided in UniProt-COVID-19 portal (https://covid-19.uniprot.org), which is ideal for fast interpretation (Figure 5B). Technical details about the construction of COVID-19 graphs are given in the Methods section.

It is interesting to observe the indirect relations between diseases/phenotypes in these KGs and COVID-19 over the incorporated host proteins and enriched pathways, and between COVID-19 and our *in silico* predicted drugs, as they may reveal further evidence to be utilized against COVID-19 (Figure 5A and B). For this, we conducted a short literature-based validation study and found that many of these drugs have already been experimented at preclinical or clinical stages for potential COVID-19 treatments (Supplementary Table S6). Although these preclinical and clinical studies are required to be completed before assessing the efficacy of these drugs, it is possible to state that CROssBAR returned a few potential drugs (along with the ones that have already been tested/used as part of different COVID-19 treatment strategies), that might be interesting to explore further with more directed *in silico* and/or *invitro / in vivo* approaches. Please refer to Supplementary Material section 1 for more information on this topic.

### *In vitro* comparative study on liver cells

**Figure 6.**
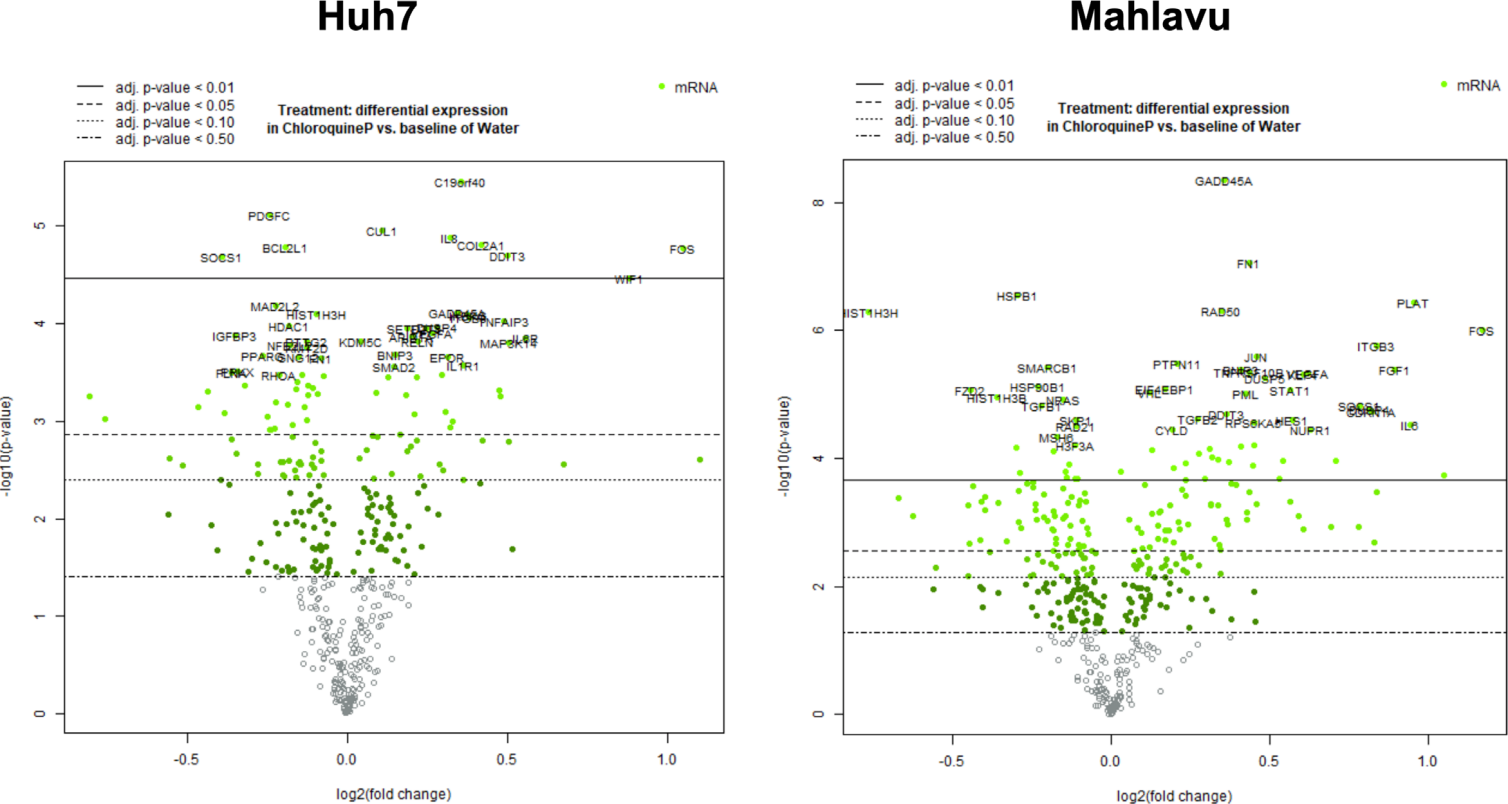
*In vitro* experimental results: volcano plots displaying differentially expressed genes in Chloroquine treated liver cells (Huh7 and Mahlavu). We checked the interaction between the significant DEGs (Supplementary Table S2) and genes in the large-scale COVID-19 KG, and applied Fisher's exact test to analyse the significance of the presence of 36 DEGs on the KG (Supplementary Table S4) as opposed to the non-DEGs in the multiplex panel of the gene expression analysis platform (NanoString). The results indicated that DEGs were significantly overrepresented (*P*-value = 1.5e–05).

Although COVID-19 is a respiratory disease and lung lesions have been considered the major damage caused by SARS‐CoV‐2, liver injury has also been reported in about one-third of hospitalized patients infected with the virus and the majority of COVID-19 patient deaths are associated with cytokine storm/release syndrome resulting in multi organ damage (39). Hence, with the aim of indicating the biological relevance of the information in CROssBAR-KGs, we conducted *in vitro* experimentation on drug treated liver cancer cell-lines and comparatively analyzed the results on both COVID-19 KGs. Chloroquine (CQ) phosphate has been used in treatment of COVID-19 with controversies (40) which was later revoked by FDA (https://www.fda.gov/news-events/press-announcements/coronavirus-covid-19-update-fda-revokes-emergency-use-authorization-chloroquine-and). CQ is an anti-inflammatory drug that has been used in autoimmune diseases and can significantly alter the production of pro-inflammatory and anti-inflammatory cytokines. We investigated the effect CQ on normal hepatocytes like Huh7 cells and poorly differentiated Mahlavu cells. Cells were treated with CQ and the differentially expressed gene (DEG) data were acquired from a large multiplex panel of genes using the NanoString platform (Figure 6). Our experimental data indicated significant alterations in JAK/STAT, PI3K, MAPK and other pathways involving cytokine production in liver cells (the full pathway list: Supplementary Table S3). These pathways were also presented in KGs along with additional cytokine related pathways, such as interleukin signaling, along with dense connections to other biological components in COVID-19 CROssBAR-KGs, which is biologically expected considering the mode of action of CQ in COVID-19. This way, we showed a correlation between *in vitro* experimental results (i.e. biological mechanism-based changes on CQ treated cells) and the contents of the knowledge graph that is constructed as a result of a relevant disease term query, completely independent from the results of our *in vitro* experiment. Since the information about the relations of these pathways to COVID-19 and CQ is not directly obtained from our source databases, this result indicates the biological relevance of the data presented by CROssBAR. In this sense, CROssBAR system can be utilized towards the systematic analysis of pharmacological effects of drugs as it brings relevant pieces of biological data together which can be manually explored by the expert user to build new hypotheses.

### Analysis of knowledge graph diversity and stability

Highly studied biological/biomedical entities (e.g. TP53 gene, JAK-STAT signalling pathway, etc.) usually have high a number of recorded relationships in databases. Consequently, they often appear in biological networks or in the resulting overrepresented term lists of gene set enrichment analyses. In CROssBAR, we aim to construct knowledge graphs with specialized content specific to the corresponding query term(s), thus, we expect to observe diversity in our KGs. Apart from that, we aim to produce stable outputs, meaning that, similar queries (i.e. searches with biologically related terms) should produce KGs with similar content, in terms of incorporated nodes and edges. With the aim of investigating both diversity and stability of CROssBAR KGs constructed as output of different biological/biomedical term queries, we conducted two experiments. The first one is a use case analysis, in which we independently queried three different diseases (types of cancer), the first two of which are similar to each other in terms of the affected biological mechanisms, and the third one is relatively dissimilar to the first two in the same sense. For this, we selected breast cancer, ovarian cancer, and osteosarcoma, respectively. The reason behind selecting another type of cancer as the third disease (instead of, for example, a rare disease, which would be highly unrelated to the first 2 diseases) was to create a rather realistic use case scenario that would allow us to observe the issues related to graph diversity, if there are any. Breast cancer and ovarian cancer are both associated with mutations and/or overexpression/amplification in certain genes (e.g. BRCA1, BRCA2, PIK3C, ERBB2, etc.) and aberrations in related pathways, which exhibits a risk of co-occurrence in women (41–43). On the other hand, osteosarcoma, the most common type of primary bone cancer, does not have a known direct relationship with breast or ovarian cancers. Besides, primary osteosarcomas of the breast and ovary are reported as very rare malignancies (44,45). Therefore, we expected to observe shared mechanisms/terms between KGs of breast and ovarian cancers, whereas the KG of osteosarcoma was expected to be relatively more diverse.

**Figure 7.**
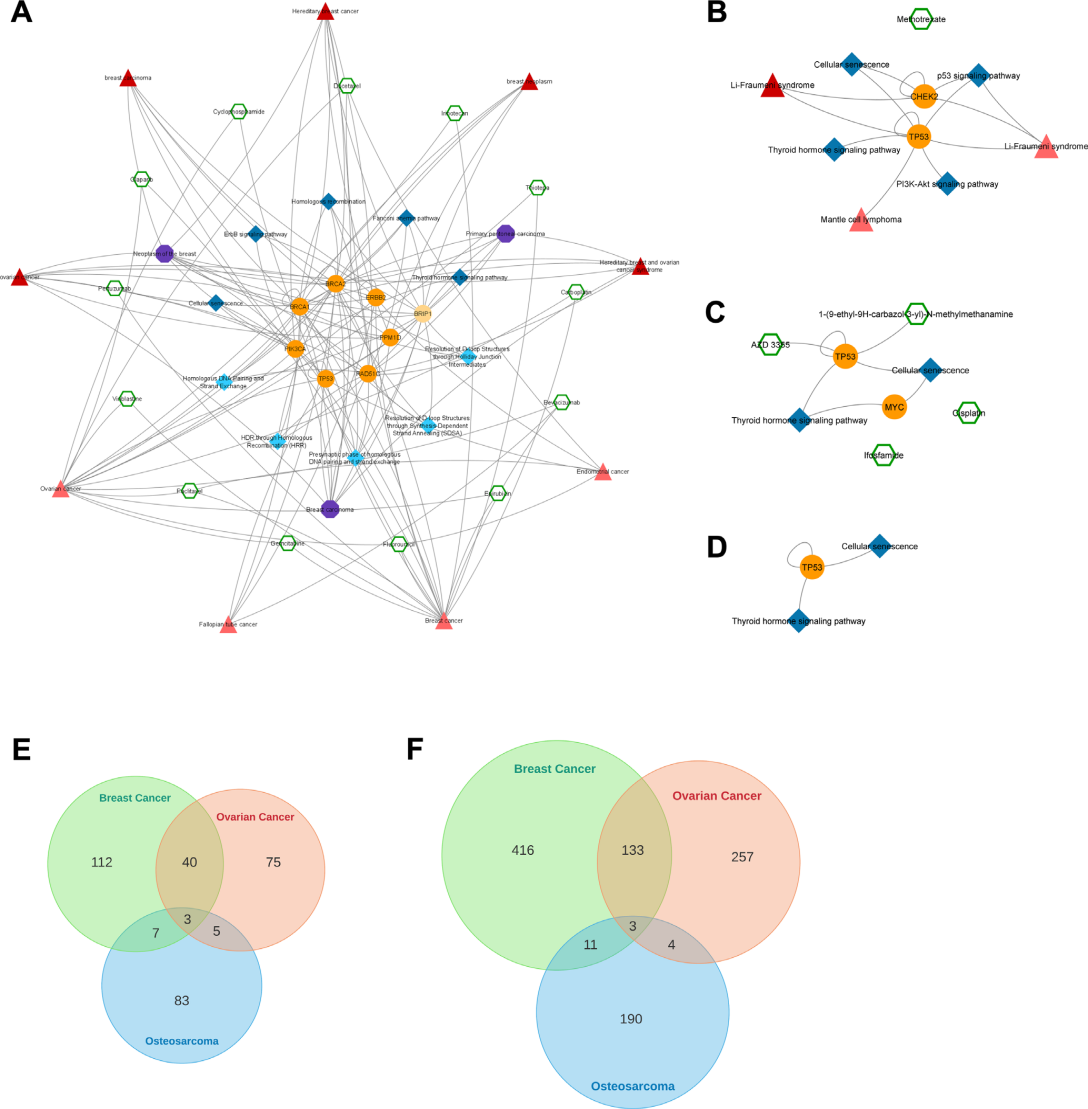
CROssBAR knowledge graph diversity analysis use case, intersection graphs between: (**A**) breast cancer and ovarian cancer, (**B**) breast cancer and osteosarcoma, (**C**) ovarian cancer and osteosarcoma, (**D**) breast cancer, ovarian cancer, and osteosarcoma (triple-wise) queries. Venn diagrams displaying the statistics of shared: (**E**) nodes and (**F**) edges, between KGs of different query terms.

We queried CROssBAR with these disease terms using default parameter values (i.e. the number of nodes to be included in KGs for each biological/biomedical component is 10, organism: human, only include reviewed protein entries from the UniProtKB/Swiss-Prot database) to construct the KGs. The resulting graphs are composed of 162 nodes and 563 edges for breast cancer, 123 nodes and 397 edges for ovarian cancer, and 98 nodes and 208 edges for osteosarcoma, and displayed in Supplementary Figure S3. After that, we calculated pairwise and triple-wise intersections between the contents of these three KGs. Graphs that are composed of intersecting nodes and edges are given together with Venn diagram-based statistics in Figure 7. We observed that the content-based identity (i.e. presence of the same nodes and edges) between KGs of breast and ovarian cancers is around 30%, whereas the overall identity between breast and osteosarcoma, and between ovarian and osteosarcoma are both ∼6%. It is also important to note that both breast and ovarian cancer graphs contain the other disease as a similar disease node. It is observed from Figure 7A and B that both BRCA1 and BRCA2 genes are presented in breast-ovarian intersection, in addition to well-known cancer driver genes such as TP53, PIK3CA and ERBB2. Breast cancer and ovarian cancer searches also contain other common associations such as pathways, phenotypes, drugs and other diseases (e.g. ErbB signaling pathway, primary peritoneal carcinoma, paclitaxel, fallopian tube cancer, etc.). Their differences are based on known and predicted bioactive compounds, due to the fact that these are selected from large pools of compounds that have direct relationship to the genes/proteins in the corresponding graph. When we omit ligands and only focus on the biological mechanism related components, the graph identity between breast and ovarian cancers is ∼35%. On the other hand, breast/ovarian and osteosarcoma intersection only included 3 nodes and 3 edges that involve the TP53 gene (Figure 7D), which was expected since TP53 mutations are critical in almost all types of cancer (46). The results of use case analysis indicated that CROssBAR constructs diverse and stable graphs specific to the user query term(s).

Since the first analysis was only a use case conducted on three sample disease queries, we decided to further investigate the matter with a quantitative test on a larger dataset. In our second experiment, we aimed to evaluate whether highly studied, and thus highly connected biological/biomedical entities tend to be presented in our graphs with high frequencies. This would be undesirable as it would mean certain terms usually end up in the graphs no matter what is searched for (i.e. the problem of limited diversity). To test this, we selected 20 terms from each biological/biomedical component (a total of 140 terms) that are among the most connected, by checking the number of their associations (degree) to different genes/proteins in our database. Names and identifiers of selected 140 terms are given in Supplementary Table S7. After that, we checked how many times these highly connected terms are presented in CROssBAR KGs. First, to construct these graphs, we queried randomly selected genes/proteins, Reactome and KEGG pathways, EFO and KEGG diseases, HPO terms, drugs and compounds one by one, and in combination with each other, on the CROssBAR web-service, resulting in a total of 1365 KGs. These query terms are listed in Supplementary Table S8, and resulting KGs can be found in our GitHub repository in json format. To evaluate whether selected highly connected terms are overly represented in CROssBAR KGs we applied Fisher's exact test independently for each term with the null hypothesis stating that the corresponding term is presented in KGs with an observed frequency (i.e. for a term *D*, observed frequency is given by: *g_D_*/*G*, where *g_D_*: number of KGs where term *D* is presented, and *G*: total number of KGs in the analysis) same as its expected frequency based on its general connectivity (i.e. for a term *D*, expected frequency is given by: t*M_D_/*M_sum_*, where *t*: number of terms/nodes in each KG from the same biological component as term *D*, *M_D_*: the total number of genes/proteins that are associated with term *D*, *M_sum_*: the total number of associations between all genes/proteins and all terms in the same biological component as term *D*). In the case that the null hypothesis is true, we would conclude that the system is not successful in terms of eliminating promiscuous/hub terms, and they are frequently presented in KGs probably because they are connected to many other terms in the database. On the other hand, a statistically significant deviation from the null hypothesis with an observed frequency of presence in KGs lower than the expected frequency would indicate that these hub terms are not presented in KGs as it would be expected based on their high connectedness, instead, they are successfully eliminated by our pipeline. We left compounds out of this analysis since their expected frequency values are extremely low due to their high number (e.g. 654 051 compounds have at least 1 target association). The results of this analysis are displayed in Supplementary Figure S4 as bar graphs drawn for each of the 140 terms, where observed and expected frequencies are shown within overlapping bars with different colours (‘*’ indicate that the corresponding observed frequency is significantly lower compared to the expected frequency). These results are also displayed in Supplementary Table S7 together with contingency table values used in statistical testing and the resulting significance (*P*-values). It is observed from the results of this analysis that 117 out of 140 highly connected terms are significantly less represented in CROssBAR KGs compared to their expected frequencies. Among these 117 terms, 22 highly connected ones (e.g. 14 HPO terms, 2 Reactome pathways and 6 drugs) were not presented in any KGs at all.

Furthermore, with the aim of calculating the diversity of graphs independent from any set of pre-selected terms, we calculated pairwise node identity percentages between all KG pair combinations (930 930 measurements between pairs of 1365 KGs) and drew a histogram of these values in log scale (Supplementary Figure S5). This histogram indicates that the node identity distribution roughly follows a power law distribution in the linear-scale, except for 106 graph pairs with a node identity value of 100%. We investigated these cases and found out that they either belong to query terms from two different source databases that indicate the exact same biological entity (e.g. disease entries from EFO and KEGG databases: ‘Orphanet:98820: Familial focal epilepsy with variable foci’ and ‘H02214: Familial focal epilepsy with variable foci’) or query terms with a close semantic relationship in the respective ontology (e.g. phenotype terms: ‘HP:0012693: Abnormal thalamic size’ and ‘HP:0012695: Decreased thalamic volume’, or Reactome pathways: ‘R-SSC-937039: IRAK1 recruits IKK complex’ and ‘R-SSC-975144: IRAK1 recruits IKK complex upon TLR7/8 or 9 stimulation’), that have the same gene/protein associations. As a result, they should not be taken into account. The mean node identity value of the distribution is 0.9% (dashed vertical line in Supplementary Figure S5), which is significantly lower compared to the identity value observed between KGs of 2 cancer types with similar biological mechanisms (i.e. breast and ovarian cancers with 30% pairwise node identity), even lower than the identity value observed between KGs of 2 dissimilar types of cancer (i.e. breast/ovarian cancers and osteosarcoma with 8% pairwise node identity) in the use case experiment given above. It is also important to note that, approximately 98.8% of the graph pairs have less than 10% node identity values, indicating the high diversity of CROssBAR KGs.

Finally, we calculated observed frequencies of all terms that are presented in our 1365 randomly generated KGs and plotted the results as biological component specific bar graphs, in which different terms are presented on the horizontal axis and their respective observed frequencies are shown on the vertical axis (terms are ranked from the highest observed frequency to the lowest) in Supplementary Figure S6. On each panel, terms from a distinct biological component are shown, together with their mean values as dashed lines. As shown in Supplementary Figure S6, observed frequency values of even the most frequent terms are considerably low (between 0.012 and 0.055) for all components except KEGG pathways. Moreover, these most frequent terms only constitute a very small portion of the total number of terms in their respective components, which is also indicated by low component-wise mean observed frequency values (dashed lines). For example, the mean frequency value considering core proteins is 0.0025, meaning that, on average a gene/protein is presented in only 1 out of 400 different KGs. Core genes/proteins with the highest observed frequencies are LNMA (P02545), MAPT (P10636) and RAB9A (P51151) with frequency values of 0.057, 0.043 and 0.036, respectively. KEGG pathways are presented in KGs with a mean observed frequency of 0.035 (highest among all biological components), and the most frequent pathways are ‘Metabolic pathways’ (hsa01100), ‘Neuroactive ligand-receptor interaction’ (hsa04080), and ‘PI3K-Akt signaling pathway’ (hsa04151) with 0.227, 0.123 and 0.100, respectively. This was expected since the total number of KEGG signalling pathways are 248 and 10 of them are included in each KG. Also, the one with the highest frequency, ‘Metabolic pathways’, is an umbrella term containing several pathways. A list of frequent terms from all components are provided with their respective observed frequency values in Supplementary Table S9.

### Graph construction runtime tests

Building a CROssBAR knowledge graph is a complex procedure that involved multiple rounds of API queries and quantitative analyses of query results to select biological terms to be represented as nodes, which is followed by the finalization of edges in-between. With the aim of observing the practicality of this procedure, we conducted runtime tests where we measured the time (in seconds) that pass from submitting the initial user query, to the finalization of the output KG. For this, we utilized the same 1365 random user queries explained in the previous section, which are composed of single term searches of 198 genes/proteins, 92 Reactome pathways, 100 KEGG pathways, 99 EFO diseases, 100 KEGG diseases, 199 HPO terms, 199 drugs and 186 compounds, together with 192 combinatory queries composed of one random term from each component (i.e. gene/protein, pathway, disease, phenotype, drug and compound). The actual query terms are given in Supplementary Table S8. The resulting runtimes are shown in Supplementary Figure S7 as histograms, where queries of distinct components are given in different panels, and median times are indicated by vertical dashed lines. As observed from Supplementary Figure S7, runtimes are variable both between the queries of the same component and across different components. Among single term queries, drugs and Reactome pathways constitute the fastest queries with median runtimes of 30 and 29 s, respectively, and HPO terms constitute the slowest with 62 s. Besides, combinatory term queries took ∼70 s on average. These results indicate that, on average, it is practical to query CROssBAR web-service and generate KGs on-the-fly. It is also important to note that runtimes are approximately linearly correlated with the number of collected core genes/proteins, and thus, querying terms that are associated with high number of genes/proteins (i.e. complex signalling pathways, generic HPO terms, etc.) can take significantly longer compared to mean times shown here. Runtimes also depend on the availability of servers.

## DISCUSSION

In this study, we presented CROssBAR, a new system that consumes large-scale biomedical data from various resources and distils it to present a coherent piece of information, relevant to a user-queried biomedical concept, communicated via heterogeneous knowledge graphs. To build CROssBAR, we constructed a NoSQL database and stored the data, developed deep-learning based models to enrich the data at hand by predicting missing DTI links, built networks of integrated information containing genes/proteins, diseases/phenotypes, biological processes/pathways and drugs/compounds, and presented all these to the life-sciences research community in an open access, user friendly web-service. Our main objective in developing CROssBAR was bringing the biological big-data together and presenting it to the user in a way that is easy to interpret. It is up to the user to evaluate the integrated and enriched data, and to draw conclusions from it. We expect that our well-maintained and continuously developed service will be adopted by researchers from various domains of life-sciences such as systems biology, cancer research and drug discovery, to aid their investigations by exploring high-level, indirect relations between specific entities/terms across different biological and biomedical concepts.

The incorporation of pathway-related information (both signaling and metabolic pathways) in CROssBAR is done within a membership-based approach, where pathways are expressed on the graph as single nodes, and the nodes of pathway member proteins are connected to them via edges. This approach leaves out the detailed reaction-based mechanistic information provided in pathway databases such as Reactome and KEGG pathways; however, the inclusion of this information via applying a pathway resource styled network approach would prevent the generation of large heterogeneous networks composed of tens of different pathways and other components. Nevertheless, it is possible to explore these pathways in detail using the provided links, which takes the user to the corresponding page on the pathway database. Both Reactome and KEGG pathways provide the same type of biological information at the level of large-scale biological processes; however, Reactome also divides these processes into sub-pathways, whereas KEGG only provides the pathway information at a generic level. In CROssBAR, due to the way the overrepresentation analysis is done, specific sub-pathways are incorporated from the Reactome database in most cases, whereas, the generic pathway information is incorporated to KGs via KEGG. As a result, pathway information is displayed at different levels of specificity, and thus, not redundant in knowledge graphs.

We presented a use case of the system by constructing two COVID-19 knowledge graphs. First, the large-scale version, in which nearly the whole of the COVID-19 related data recently accumulated in our source databases are integrated, organized and presented. Second, the simplified version, where the aim was to provide user with a source that is suitable for quick exploration, since the large-scale KG is not easily explorable due to its huge size. Since most of the COVID-19 related data are still being integrated into biological databases, the data could not be automatically pulled to the CROssBAR database at the time of writing this manuscript. As a result, we had to make manual interventions to obtain the data from CROssBAR data resources. We applied the same knowledge graph construction methodology incorporated in CROssBAR; however, we also conducted manual curation to a certain extent. We saved the pre-constructed KGs, which are directly accessible and viewable through the links given on our web-service (https://crossbar.kansil.org/covid_main.php). It is also important to note that, due to the content of integrated data resources, CROssBAR primarily contains rare and complex disease data, and mostly leaves infectious diseases out. Nevertheless, the constructed COVID-19 graphs provide rich biomedical information.

At each step of developing the CROssBAR system, we considered its alignment with FAIR data principles (47) to contribute to the movement towards fully open access, standard and easily (re)usable biomedical data. Since we mainly integrate data from other open access biomedical resources, we did not create new identifiers; however, we extensively share the identifiers in the source databases together with links, with the aim of making our data findable. Considering accessibility, CROssBAR system is reachable through various ways including the full open access API and web-service (interactively displaying the knowledge graphs) and through the project repositories (e.g. https://github.com/cansyl/CROssBAR) which includes the source codes and datasets. CROssBAR data is highly interoperable as it mainly integrates heterogeneous biomedical data that is normally scattered throughout many independent data resources, and presents it in a coherent and standardized form. CROssBAR is released under the Creative Commons Attribution 4.0 (CC BY 4.0) license which mostly prevents restrictions on the downstream reuse of data, thus aligning it with the reusability item of FAIR. It is also important to note that, since CROssBAR is mainly obtaining its data from other open access biomedical data services, their compliance with FAIR principles affects CROssBAR’s alignment as well, even though this effect is only indirect. For this reason, we plan to encourage biomedical data providers to consider prioritizing FAIR compliance while developing their own resources.

As future work, we plan to integrate additional biomedical resources to CROssBAR such as (i) the cell-type/tissue based transcriptomics, proteomics and other omics based data (e.g. glycomics, lipidomics, etc.) and genomic variation data (e.g. missense mutations, copy number variations, etc.) in relation to both biomolecules and patients (i.e. from TCGA) especially to be associated with diseases, (ii) evolutionary information such as paralogy and orthology relations across biomolecules of different species, (iii) biomolecular function annotations (e.g. GO terms, EC numbers), (iv) data sources and tools for infectious diseases and their mechanisms and (v) literature-based information (e.g. articles) as evidences of incorporated relationships. Furthermore, we plan to enhance evidence-based edge weights/labels in our knowledge graphs (currently we have PPI confidence scores and DTI bioactivity values as edge attributes), and also, to incorporate edge weights to the enrichment analysis procedure, with the aim of assessing the relevance of each biological term to the user's query with a higher specificity. We also plan to incorporate *in silico* relationship predictions between different layers of the biomedical data, in addition to compound/drug-target protein interactions, such as gene/protein-disease/phenotype, gene/protein-function and drug-disease associations, by both constructing novel in-house prediction systems and by incorporating approaches from the literature, including the state-of-the-art graph learning-based link prediction methods. It is also important to state that, we continuously focus on providing a well-maintained, fast, scalable and practical service to the life-sciences research community with both data updates and back-end/front-end improvements in our resource.

## DATA AVAILABILITY

CROssBAR web-service (including a tutorial on how to use the service) is available at https://crossbar.kansil.org, CROssBAR database is available through our API at https://www.ebi.ac.uk/Tools/crossbar/swagger-ui.html, all of the datasets, results and the source code of this project are available for download at https://github.com/cansyl/CROssBAR, additional information is available in the CROssBAR project web-site at https://cansyl.metu.edu.tr/crossbar.

## Supplementary Material

gkab543_Supplemental_File

## References

[1] Fabregat A., Jupe S., Matthews L., Sidiropoulos K., Gillespie M., Garapati P., Haw R., Jassal B., Korninger F., May B. et al. The reactome pathway knowledgebase. Nucleic Acids Res. 2018; 46:D649–D655. 29145629 10.1093/nar/gkx1132 PMC5753187

[2] Kanehisa M., Goto S., Sato Y., Kawashima M., Furumichi M., Tanabe M. Data, information, knowledge and principle: back to metabolism in KEGG. Nucleic Acids Res. 2014; 42:D199–D205. 24214961 10.1093/nar/gkt1076 PMC3965122

[3] Kutmon M., Riutta A., Nunes N., Hanspers K., Willighagen E.L., Bohler A., Mélius J., Waagmeester A., Sinha S.R., Miller R. et al. WikiPathways: capturing the full diversity of pathway knowledge. Nucleic Acids Res. 2016; 44:D488–D494. 26481357 10.1093/nar/gkv1024 PMC4702772

[4] Szklarczyk D., Gable A.L., Lyon D., Junge A., Wyder S., Huerta-Cepas J., Simonovic M., Doncheva N.T., Morris J.H., Bork P. et al. STRING v11: protein–protein association networks with increased coverage, supporting functional discovery in genome-wide experimental datasets. Nucleic Acids Res. 2019; 47:D607–D613. 30476243 10.1093/nar/gky1131 PMC6323986

[5] Szklarczyk D., Santos A., Von Mering C., Jensen L.J., Bork P., Kuhn M. STITCH 5: augmenting protein–chemical interaction networks with tissue and affinity data. Nucleic Acids Res. 2016; 44:D380–D384. 26590256 10.1093/nar/gkv1277 PMC4702904

[6] Franz M., Rodriguez H., Lopes C., Zuberi K., Montojo J., Bader G.D., Morris Q. GeneMANIA update 2018. Nucleic Acids Res. 2018; 46:W60–W64. 29912392 10.1093/nar/gky311 PMC6030815

[7] Whetzel P.L., Noy N.F., Shah N.H., Alexander P.R., Nyulas C., Tudorache T., Musen M.A. BioPortal: enhanced functionality via new Web services from the National Center for Biomedical Ontology to access and use ontologies in software applications. Nucleic Acids Res. 2011; 39:W541–W545. 21672956 10.1093/nar/gkr469 PMC3125807

[8] Côté R., Reisinger F., Martens L., Barsnes H., Vizcaino J.A., Hermjakob H. The ontology lookup service: bigger and better. Nucleic Acids Res. 2010; 38:W155–W160. 20460452 10.1093/nar/gkq331 PMC2896109

[9] Liekens A.M., De Knijf J., Daelemans W., Goethals B., De Rijk P., Del-Favero J. BioGraph: unsupervised biomedical knowledge discovery via automated hypothesis generation. Genome Biol. 2011; 12:R57. 21696594 10.1186/gb-2011-12-6-r57 PMC3218845

[10] Pareja-Tobes P., Tobes R., Manrique M., Pareja E., Pareja-Tobes E. Bio4j: a high-performance cloud-enabled graph-based data platform. 2015; bioRxiv doi:20 March 2015, preprint: not peer reviewed 10.1101/016758.

[11] Himmelstein D.S., Lizee A., Hessler C., Brueggeman L., Chen S.L., Hadley D., Green A., Khankhanian P., Baranzini S.E. Systematic integration of biomedical knowledge prioritizes drugs for repurposing. Elife. 2017; 6:e26726. 28936969 10.7554/eLife.26726 PMC5640425

[12] Messina A., Pribadi H., Stichbury J., Bucci M., Klarman S., Urso A. BioGrakn: a knowledge graph-based semantic database for biomedical sciences. Conference on Complex, Intelligent, and Software Intensive Systems. 2017; Cham Springer 299–309.

[13] Messina A., Fiannaca A., La Paglia L., La Rosa M., Urso A. BioGraph: a web application and a graph database for querying and analyzing bioinformatics resources. BMC Syst. Biol. 2018; 12:98. 30458802 10.1186/s12918-018-0616-4 PMC6245492

[14] Yuan J., Jin Z., Guo H., Jin H., Zhang X., Smith T., Luo J. Constructing biomedical domain-specific knowledge graph with minimum supervision. Knowl. Inf. Syst. 2020; 62:317–336.

[15] Cong Q., Feng Z., Li F., Zhang L., Rao G., Tao C. Constructing Biomedical Knowledge Graph Based on SemMedDB and Linked Open Data. 2018 IEEE International Conference on Bioinformatics and Biomedicine (BIBM). 2019; IEEE 1628–1631.

[16] Nicholson D.N., Himmelstein D.S., Greene C.S. Expanding a database-derived biomedical knowledge graph via multi-relation extraction from biomedical abstracts. 2020; bioRxiv doi:31 January 2020, preprint: not peer reviewed 10.1101/730085. PMC9578183 36258252

[17] Ernst P., Siu A., Weikum G. Knowlife: a versatile approach for constructing a large knowledge graph for biomedical sciences. BMC Bioinformatics. 2015; 16:157. 25971816 10.1186/s12859-015-0549-5 PMC4448285

[18] Livingston K.M., Bada M., Baumgartner W.A., Hunter L.E. KaBOB: ontology-based semantic integration of biomedical databases. BMC Bioinformatics. 2015; 16:126. 25903923 10.1186/s12859-015-0559-3 PMC4448321

[19] Waagmeester A., Stupp G., Burgstaller-Muehlbacher S., Good B.M., Griffith M., Griffith O.L., Hanspers K., Hermjakob H., Hudson T.S., Hybiske K. et al. Science forum: Wikidata as a knowledge graph for the life sciences. Elife. 2020; 9:e52614. 32180547 10.7554/eLife.52614 PMC7077981

[20] Turki H., Shafee T., Taieb M.A.H., Aouicha M.B., Vrandečić D., Das D., Hamdi H. Wikidata: a large-scale collaborative ontological medical database. J. Biomed. Inform. 2019; 99:103292. 31557529 10.1016/j.jbi.2019.103292

[21] Richardson P., Griffin I., Tucker C., Smith D., Oechsle O., Phelan A., Rawling M., Savory E., Stebbing J. Baricitinib as potential treatment for 2019-nCoV acute respiratory disease. Lancet. 2020; 395:e30. 32032529 10.1016/S0140-6736(20)30304-4 PMC7137985

[22] Stebbing J., Phelan A., Griffin I., Tucker C., Oechsle O., Smith D., Richardson P. COVID-19: combining antiviral and anti-inflammatory treatments. Lancet Infect. Dis. 2020; 20:400–402. 32113509 10.1016/S1473-3099(20)30132-8 PMC7158903

[23] Rifaioglu A., Sinoplu E., Atalay V., Martin M., Cetin-Atalay R., Doğan T. DEEPScreen: high performance drug-target interaction prediction with convolutional neural networks Using 2-D structural compound representations. Chem. Sci. 2020; 11:2531–2557. 33209251 10.1039/c9sc03414e PMC7643205

[24] Rifaioglu A., Cetin-Atalay R., Kahraman D.C., Dogan T., Martin M., Atalay V. MDeePred: novel multi-channel protein featurization for deep learning based binding affinity prediction in drug discovery. Bioinformatics. 2021; 37:693–704. 33067636 10.1093/bioinformatics/btaa858

[25] Rivals I., Personnaz L., Taing L., Potier M.C. Enrichment or depletion of a GO category within a class of genes: which test. Bioinformatics. 2007; 23:401–407. 17182697 10.1093/bioinformatics/btl633

[26] Gordon D.E., Jang G.M., Bouhaddou M., Xu J., Obernier K., White K.M., O’Meara M.J., Rezelj V.V., Guo J.Z., Swaney D.L. et al. A SARS-CoV-2 protein interaction map reveals targets for drug repurposing. Nature. 2020; 583:459–468. 32353859 10.1038/s41586-020-2286-9 PMC7431030

[27] Rifaioglu A.S., Atas H., Martin M.J., Cetin-Atalay R., Atalay V., Doğan T. Recent applications of deep learning and machine intelligence on in silico drug discovery: methods, tools and databases. Brief. Bioinform. 2019; 20:1878–1912. 30084866 10.1093/bib/bby061 PMC6917215

[28] Donmez A., Rifaioglu A., Acar A., Dogan T., Cetin-Atalay R.M., Atalay V. iBioProVis: interactive visualization and analysis of compound bioactivity space. Bioinformatics. 2020; 36:4227–4230. 32407491 10.1093/bioinformatics/btaa496 PMC7454317

[29] Wang Z., Zhang J., Feng J., Chen Z. Knowledge graph embedding by translating on hyperplanes. the Twenty-Eighth AAAI Conference on Artificial Intelligence. 2014; 14:AAAI 1112–1119.

[30] Shannon P., Markiel A., Ozier O., Baliga N.S., Wang J.T., Ramage D., Amin N., Schwikowski B., Ideker T. Cytoscape: a software environment for integrated models of biomolecular interaction networks. Genome Res. 2003; 13:2498–2504. 14597658 10.1101/gr.1239303 PMC403769

[31] Yeh C.T., Wu A.T., Chang P.M.H., Chen K.Y., Yang C.N., Yang S.C., Ho C.C., Chen C.C., Kuo Y.L., Lee P.Y. et al. Trifluoperazine, an antipsychotic agent, inhibits cancer stem cell growth and overcomes drug resistance of lung cancer. Am. J. Resp. Crit. Care. 2012; 186:1180–1188. 10.1164/rccm.201207-1180OC 23024022

[32] Xia Y., Jia C., Xue Q., Jiang J., Xie Y., Wang R., Ran Z., Xu F., Zhang Y., Ye T. Antipsychotic drug trifluoperazine suppresses colorectal cancer by inducing G0/G1 arrest and apoptosis. Front. Pharmacol. 2019; 10:1029. 31572198 10.3389/fphar.2019.01029 PMC6753363

[33] Huang C., Lan W., Fraunhoffer N., Meilerman A., Iovanna J., Santofimia-Castaño P. Dissecting the anticancer mechanism of trifluoperazine on pancreatic ductal adenocarcinoma. Cancers. 2019; 11:1869. 10.3390/cancers11121869 PMC6966621 31769431

[34] Hsieh Y.H., Chan H.L., Lin C.F., Liang S.H.Y., Lu M.L., McIntyre R.S., Lee Y., Lin T.C., Chiu W.C., Chen V.C.H. Antipsychotic use is inversely associated with gastric cancer risk: a nationwide population-based nested case-control study. Cancer Med. 2019; 8:4484–4496. 31183993 10.1002/cam4.2329 PMC6675741

[35] Cui C., Merritt R., Fu L., Pan Z. Targeting calcium signaling in cancer therapy. Acta Pharm. Sinica B. 2017; 7:3–17. 10.1016/j.apsb.2016.11.001 PMC5237760 28119804

[36] Xie R., Xu J., Xiao Y., Wu J., Wan H., Tang B., Liu J., Fan Y., Wang S., Wu Y. et al. Calcium promotes human gastric cancer via a novel coupling of calcium-sensing receptor and TRPV4 channel. Cancer Res. 2017; 77:6499–6512. 28951460 10.1158/0008-5472.CAN-17-0360

[37] Li X., Liu W., Wang H., Yang L., Li Y., Wen H., Ning H., Wang J., Zhang L., Li J., Fan D. Rap1 is indispensable for TRF2 function in etoposide-induced DNA damage response in gastric cancer cell line. Oncogenesis. 2015; 4:e144. 25821946 10.1038/oncsis.2015.1 PMC4491608

[38] Wu F., Zhao S., Yu B., Chen Y.M., Wang W., Song Z.G., Hu Y., Tao Z.W., Tian J.H., Pei Y.Y. et al. A new coronavirus associated with human respiratory disease in China. Nature. 2020; 579:265–269. 32015508 10.1038/s41586-020-2008-3 PMC7094943

[39] Wu J., Song S., Cao H.C., Li L.J. Liver diseases in COVID-19: etiology, treatment and prognosis. World J. Gastroentero. 2020; 26:2286. 10.3748/wjg.v26.i19.2286 PMC7243650 32476793

[40] Zhang C., Huang S., Zheng F., Dai Y. Controversial treatments: an updated understanding of the coronavirus disease 2019. J. Med. Virol. 2020; 92:1441–1448. 32219882 10.1002/jmv.25788 PMC7228369

[41] King M.C., Marks J.H., Mandell J.B. Breast and ovarian cancer risks due to inherited mutations in BRCA1 and BRCA2. Science. 2003; 302:643–646. 14576434 10.1126/science.1088759

[42] Costa R.L., Han H.S., Gradishar W.J. Targeting the PI3K/AKT/mTOR pathway in triple-negative breast cancer: a review. Breast Cancer Res. Tr. 2018; 169:397–406. 10.1007/s10549-018-4697-y 29417298

[43] Lheureux S., Gourley C., Vergote I., Oza A.M. Epithelial ovarian cancer. Lancet. 2019; 393:1240–1253. 30910306 10.1016/S0140-6736(18)32552-2

[44] Fadare O., Bossuyt V., Martel M., Parkash V. Primary osteosarcoma of the ovary: a case report and literature review. Int. J. Gynecol. Pathol. 2007; 26:21–25. 17197892 10.1097/01.pgp.0000225840.36750.a2

[45] Bahrami A., Resetkova E., Ro J.Y., Ibañez J.D., Ayala A.G. Primary osteosarcoma of the breast: report of 2 cases. Arch. Pathol. Lab. Med. 2007; 131:792–795. 17488168 10.5858/2007-131-792-POOTBR

[46] Whibley C., Pharoah P.D., Hollstein M. p53 polymorphisms: cancer implications. Nat. Rev. Cancer. 2009; 9:95–107. 19165225 10.1038/nrc2584

[47] Wilkinson M.D., Dumontier M., Aalbersberg I.J., Appleton G., Axton M., Baak A., Blomberg N., Boiten J.W., da Silva Santos L.B., Bourne P.E. et al. The FAIR Guiding principles for scientific data management and stewardship. Scientific Data. 2016; 3:160018. 26978244 10.1038/sdata.2016.18 PMC4792175

