# Supplementary material for: CROssBAR: comprehensive resource of biomedical relations with knowledge graph representations
Source: Nucleic Acids Res. 2021 Jun 28;49(16):e96. doi: 10.1093/nar/gkab543 (PMC8450100; doi:10.1093/nar/gkab543)
Supplement: gkab543_Supplemental_File [file gkab543_supplemental_file.pdf]

<u>**Supplementary Material**</u>

CROssBAR: Comprehensive Resource of Biomedical Relations with Knowledge Graph Representations

Tunca Doğan[1,2,3,4*], Heval Atas[3], Vishal Joshi[4], Ahmet Atakan[5,6], Ahmet Sureyya Rifaioglu[5,7], Esra Nalbat[3], Andrew Nightingale[4], Rabie Saidi[4], Vladimir Volynkin[4], Hermann Zellner[4], Rengul Cetin-Atalay[3,8], Maria Martin[4], Volkan Atalay[5]

[1] Department of Computer Engineering, Hacettepe University, Ankara, 06800, Turkey
[2] Institute of Informatics, Hacettepe University, Ankara, 06800, Turkey
[3] Cancer Systems Biology Laboratory, Graduate School of Informatics, METU, Ankara, 06800, Turkey
[4] European Molecular Biology Laboratory, European Bioinformatics Institute (EMBL–EBI), Hinxton, Cambridgeshire, CB10 1SD, UK
[5] Department of Computer Engineering, METU, Ankara, 06800, Turkey
[6] Department of Computer Engineering, EBYU, Erzincan, 24002, Turkey
[7] Department of Computer Engineering, İskenderun Technical University, Hatay, 31200, Turkey
[8] Section of Pulmonary and Critical Care Medicine, University of Chicago, Chicago IL, 60637, USA

* To whom correspondence should be addressed. Tel: +905055250011; Fax: +903122977194;
Email: tuncadogan@gmail.com

1. Investigation of COVID-19 Associated Drugs on the COVID-19 Knowledge Graph

CROssBAR COVID-19 knowledge graph (KG) incorporates several drugs that can be utilized for developing novel treatments against SARS-CoV-2. Several of these drugs have already been reported in the COVID-19 literature and included based on this information; however, some of them were completely new. These new drugs have been incorporated to the graph either due to the overrepresentation-based enrichment analysis (based on the COVID-19 related host genes/proteins in the graph) or predicted to interact to with host or SARS-CoV-2 proteins by our deep-learning-based tools DEEPScreen and MDeePred. Here, we demonstrate a short literature-based validation study on the relevance of these new drugs for COVID-19. Table S6 shows promising drugs in our knowledge graph together with the source (i.e., whether they entered the graph due to enrichment or predicted by DEEPScreen and/or MDeePred). It is interesting to observe that some of the drugs in this list are currently under clinical trials against COVID-19. The list includes calcineurin, IL-6 and IL-17a inhibitors such as cyclosporine, tocilizumab, and ixekizumab, which play roles in the immune system and effective against inflammatory diseases. As an immunomodulator agent, interferon beta-1a is also included in the list, inducing the synthesis of antiviral mediators by binding to type I interferon receptors. In addition to these, there are also other type of drugs in the list such as tenectaplase, vazegepant and simvastatin, which have promising clinical study results especially for the prevention of severe pulmonary damages and respiratory failures due to COVID-19. Ascorbic acid and Epigallocatechin gallate are two examples of natural products that have COVID-19 related clinical studies. Apart from these, other enriched/predicted drugs such as amlodipine (1, 2), artenimol (3)

lifitegrast (4, 5), amcinonide (6) and becatecarin (7) have been reported as potential drugs for COVID-19 via *in vitro*, *in vivo* and/or *in silico* studies including machine learning and molecular docking applications, although some of these studies are yet to be peer-reviewed. As a potent inhibitor of NF-κB activation in T-cells, rocaglamide and its derivatives may also be potential drug candidates for the treatment of COVID-19; however, there is no COVID-19 related study about these drugs in the literature yet, except from a study reviewing antiviral activity potential of rocaglamide as a flavagline (8). It is also important to mention that, further research is required to properly assess the potential of these drugs for repurposing against SARS-CoV-2 infection.

Supplementary References

1. Zhang, L. K., Sun, Y., Zeng, H., Wang, Q., Jiang, X., Shang, W. J., et al. (2020) Calcium channel blocker amlodipine besylate therapy is associated with reduced case fatality rate of COVID-19 patients with hypertension. Cell discovery, 6(1), 1-12.
2. Solaimanzadeh, I. (2020) Nifedipine and amlodipine are associated with improved mortality and decreased risk for intubation and mechanical ventilation in elderly patients hospitalized for COVID-19. Cureus, 12(5).
3. Sehailia, M. and Chemat, S. (2020) Antimalarial-agent artemisinin and derivatives portray more potent binding to Lys353 and Lys31-binding hotspots of SARS-CoV-2 spike protein than hydroxychloroquine: potential repurposing of artenimol for COVID-19. Journal of Biomolecular Structure and Dynamics, 1-11.
4. Talluri S. (2021) Molecular Docking and Virtual Screening based prediction of drugs for COVID-19. Combinatorial Chemistry & High Throughput Screening, 24(5), 716.
5. Day, C. J., Bailly, B., Guillon, P., Dirr, L., Jen, F. E. C., Spillings, B. L., et al. (2021) Multidisciplinary Approaches Identify Compounds that Bind to Human ACE2 or SARS-CoV-2 Spike Protein as Candidates to Block SARS-CoV-2-ACE2 Receptor Interactions. MBio, 12(2).
6. Anwar, M. U., Adnan, F., Abro, A., Khan, M. R., Rehman, A. U., Osama, M., et al. (2020) Combined Deep Learning and Molecular Docking Simulations Approach Identifies Potentially Effective FDA Approved Drugs for Repurposing Against SARS-CoV-2. ChemRxiv doi:10.26434/chemrxiv.12227363.v1, 09 June 2021, pre-print: not peer-reviewed.
7. Durdagi, S., Aksoydan, B., Dogan, B., Sahin, K., Shahraki, A. and Birgül-İyison, N. (2020) Screening of Clinically Approved and Investigation Drugs as Potential Inhibitors of SARS-CoV-2 Main Protease and Spike Receptor-Binding Domain Bound with ACE2 COVID19 Target Proteins: A Virtual Drug Repurposing Study. ChemRxiv doi:10.26434/chemrxiv.12032712.v2, 09 June 2021, pre-print: not peer-reviewed.
8. Schulz, G., Victoria, C., Kirschning, A. and Steinmann, E. (2021) Rocaglamide and silvestrol: a long story from anti-tumor to anti-coronavirus compounds. Natural Product Reports, 38(1), 18-23.
9. Kuleshov, M. V., Jones, M. R., Rouillard, A. D., Fernandez, N. F., Duan, Q., Wang, Z., et al. (2016) Enrichr: a comprehensive gene set enrichment analysis web server 2016 update. Nucleic acids research, 44(W1), W90-W97.
10. Liao, Y., Wang, J., Jaehnig, E. J., Shi, Z. and Zhang, B. (2019) WebGestalt 2019: gene set analysis toolkit with revamped UIs and APIs. Nucleic acids research, 47(W1), W199-W205.

Supplementary Figures

Figure S1

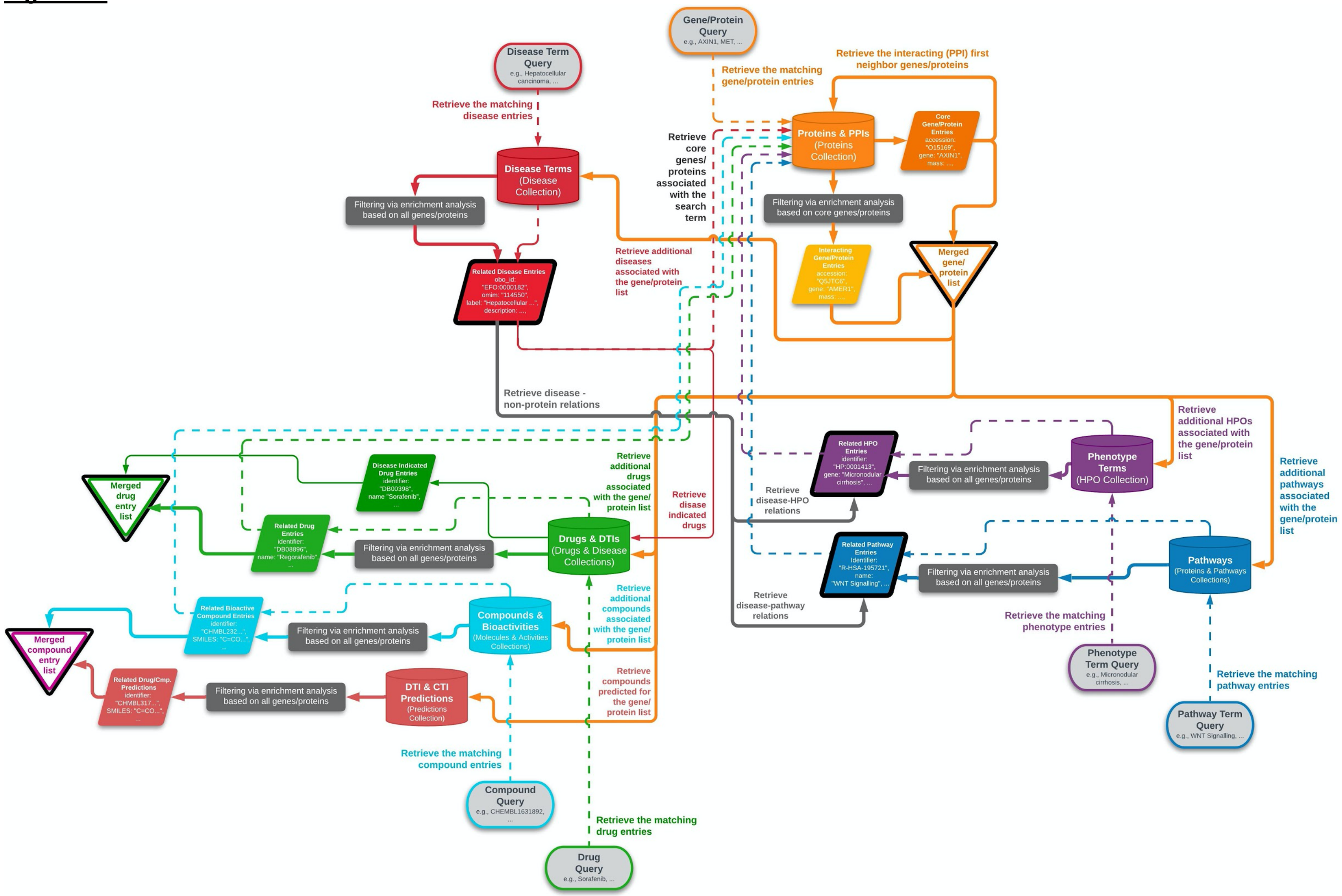

Figure S1. Full-scale work-flow of the CROssBAR knowledge graph construction process.

Figure S2

(a)

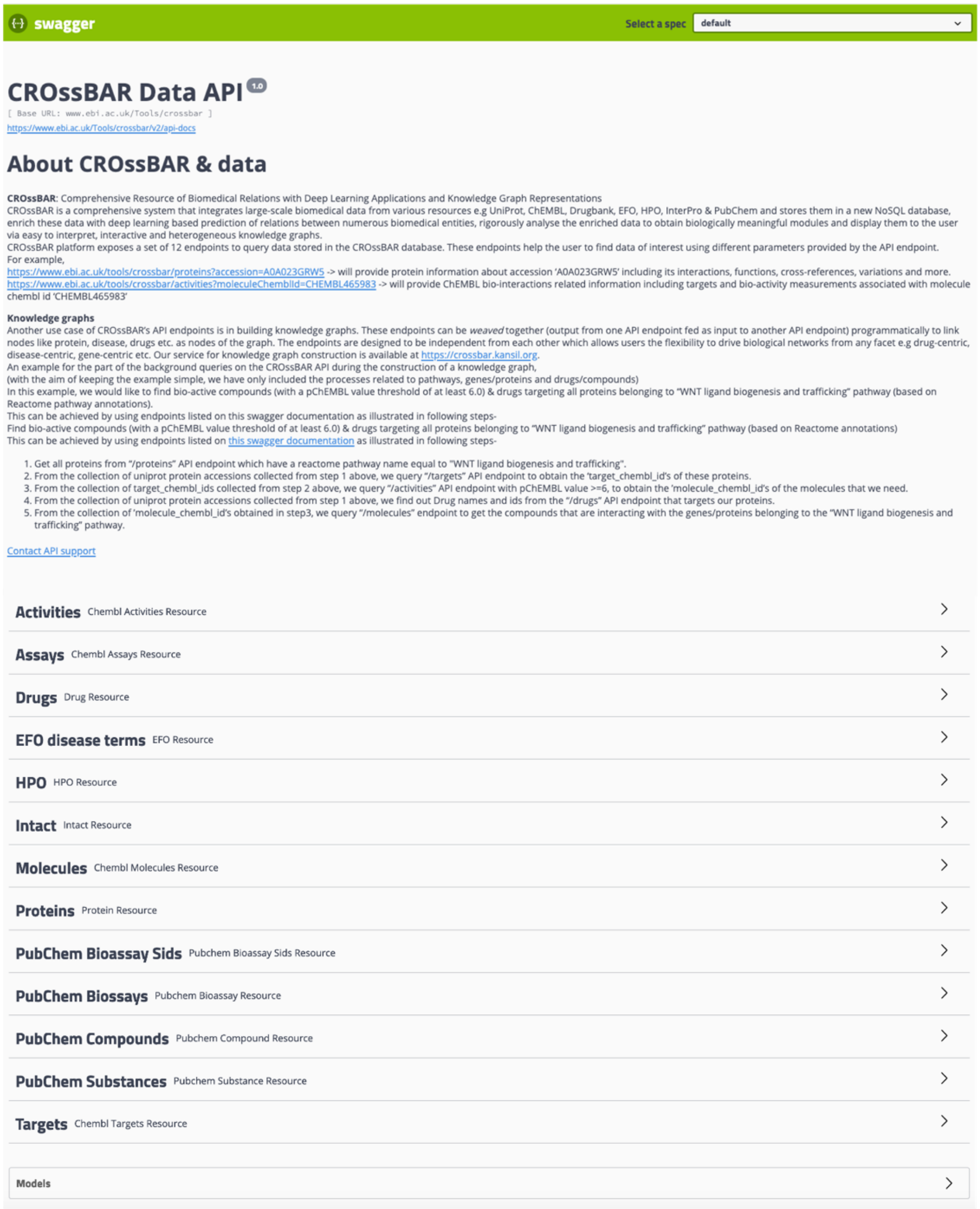
swagger
Select a spec
default
CROssBAR Data API
1.0
[Base URL: www.ebi.ac.uk/Tools/crossbar]
https://www.ebi.ac.uk/Tools/crossbar/v2/api-docs
About CROssBAR & data
CROssBAR: Comprehensive Resource of Biomedical Relations with Deep Learning Applications and Knowledge Graph Representations
CROssBAR is a comprehensive system that integrates large-scale biomedical data from various resources e.g UniProt, ChEMBL, Drugbank, EFO, HPO, InterPro & PubChem and stores them in a new NoSQL database, enrich these data with deep learning based prediction of relations between numerous biomedical entities, rigorously analyse the enriched data to obtain biologically meaningful modules and display them to the user via easy to interpret, interactive and heterogeneous knowledge graphs.
CROssBAR platform exposes a set of 12 endpoints to query data stored in the CROssBAR database. These endpoints help the user to find data of interest using different parameters provided by the API endpoint.
For example,
https://www.ebi.ac.uk/tools/crossbar/proteins?accession=A0A023GRW5 -> will provide protein information about accession 'A0A023GRW5' including its interactions, functions, cross-references, variations and more.
https://www.ebi.ac.uk/tools/crossbar/activities?moleculeChemblId=CHEMBL465983 -> will provide ChEMBL bio-interactions related information including targets and bio-activity measurements associated with molecule chembl id 'CHEMBL465983'
Knowledge graphs
Another use case of CROssBAR's API endpoints is in building knowledge graphs. These endpoints can be weaved together (output from one API endpoint fed as input to another API endpoint) programmatically to link nodes like protein, disease, drugs etc. as nodes of the graph. The endpoints are designed to be independent from each other which allows users the flexibility to drive biological networks from any facet e.g drug-centric, disease-centric, gene-centric etc. Our service for knowledge graph construction is available at https://crossbar.kansil.org.
An example for the part of the background queries on the CROssBAR API during the construction of a knowledge graph,
(with the aim of keeping the example simple, we have only included the processes related to pathways, genes/proteins and drugs/compounds)
In this example, we would like to find bio-active compounds (with a pChEMBL value threshold of at least 6.0) & drugs targeting all proteins belonging to "WNT ligand biogenesis and trafficking" pathway (based on Reactome pathway annotations).
This can be achieved by using endpoints listed on this swagger documentation as illustrated in following steps-
Find bio-active compounds (with a pChEMBL value threshold of at least 6.0) & drugs targeting all proteins belonging to "WNT ligand biogenesis and trafficking" pathway (based on Reactome annotations)
This can be achieved by using endpoints listed on this swagger documentation as illustrated in following steps-
1. Get all proteins from "/proteins" API endpoint which have a reactome pathway name equal to "WNT ligand biogenesis and trafficking".
2. From the collection of uniprot protein accessions collected from step 1 above, we query "/targets" API endpoint to obtain the 'target_chembl_id's of these proteins.
3. From the collection of target_chembl_ids collected from step 2 above, we query "/activities" API endpoint with pChEMBL value >=6, to obtain the 'molecule_chembl_id's of the molecules that we need.
4. From the collection of uniprot protein accessions collected from step 1 above, we find out Drug names and ids from the "/drugs" API endpoint that targets our proteins.
5. From the collection of 'molecule_chembl_id's obtained in step3, we query "/molecules" endpoint to get the compounds that are interacting with the genes/proteins belonging to the "WNT ligand biogenesis and trafficking" pathway.
Contact API support
Activities Chembl Activities Resource
Assays Chembl Assays Resource
Drugs Drug Resource
EFO disease terms EFO Resource
HPO HPO Resource
Intact Intact Resource
Molecules Chembl Molecules Resource
Proteins Protein Resource
PubChem Bioassay Sids Pubchem Bioassay Sids Resource
PubChem Biossays Pubchem Bioassay Resource
PubChem Compounds Pubchem Compound Resource
PubChem Substances Pubchem Substance Resource
Targets Chembl Targets Resource
Models

(b)

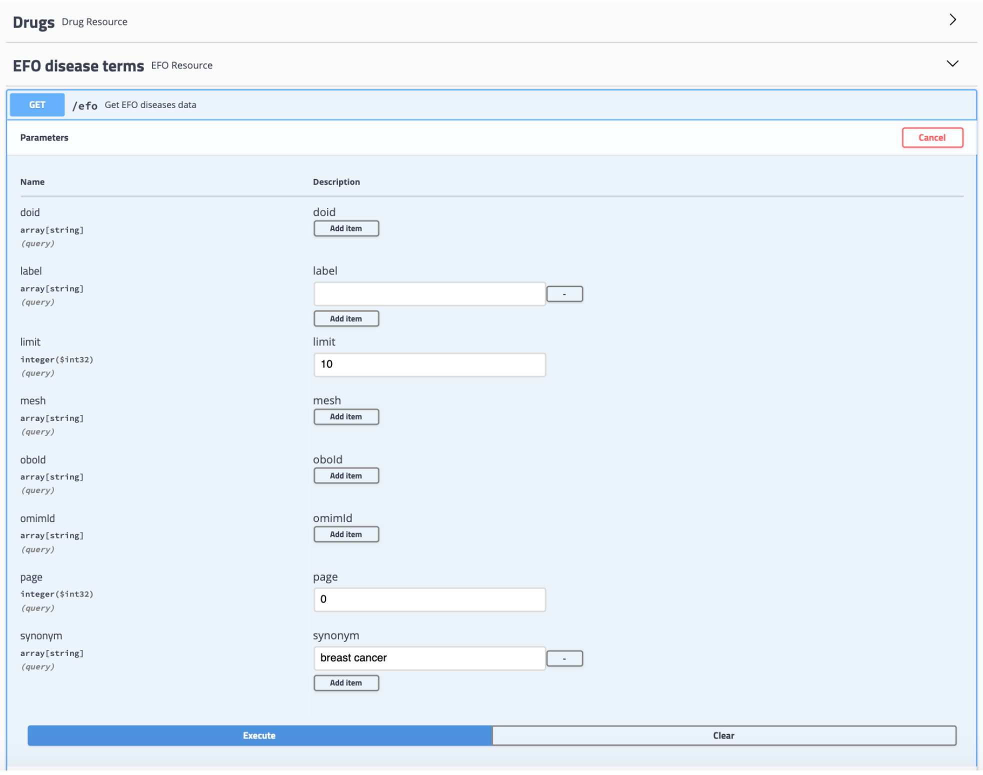

(c)

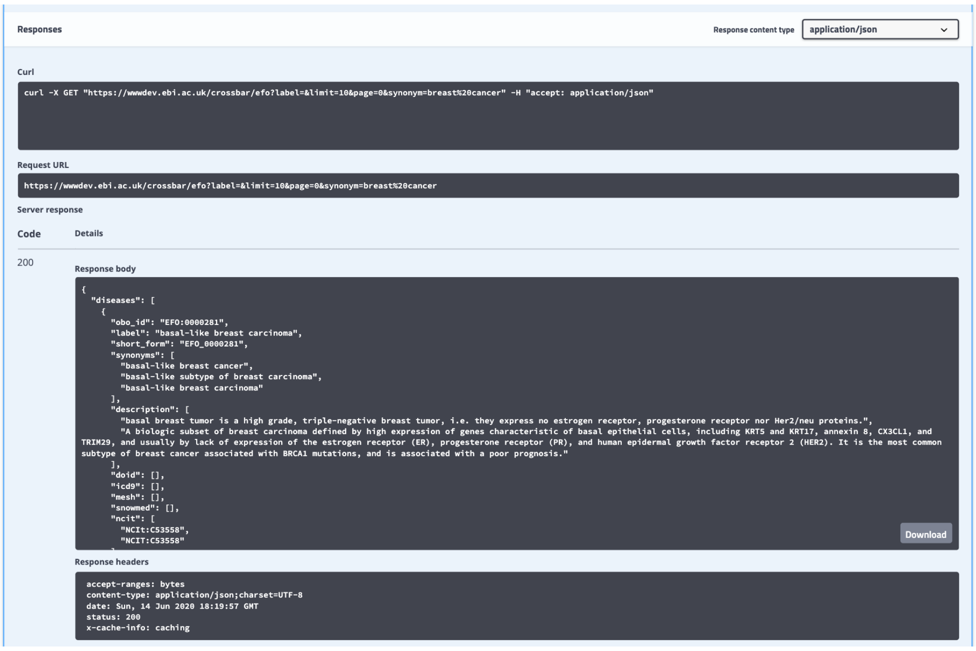

Figure S2. CROssBAR API Swagger web interface (https://www.ebi.ac.uk/Tools/crossbar/swagger-ui.html); **(a)** names of 12 endpoints at the main page; **(b)** an example API query on EFO disease terms collection with "breast cancer" searched as the synonymous term name; **(c)** query response: the same query in command line and as URL, and the list of resulting database entries that match the query together with their attributes.

(a)

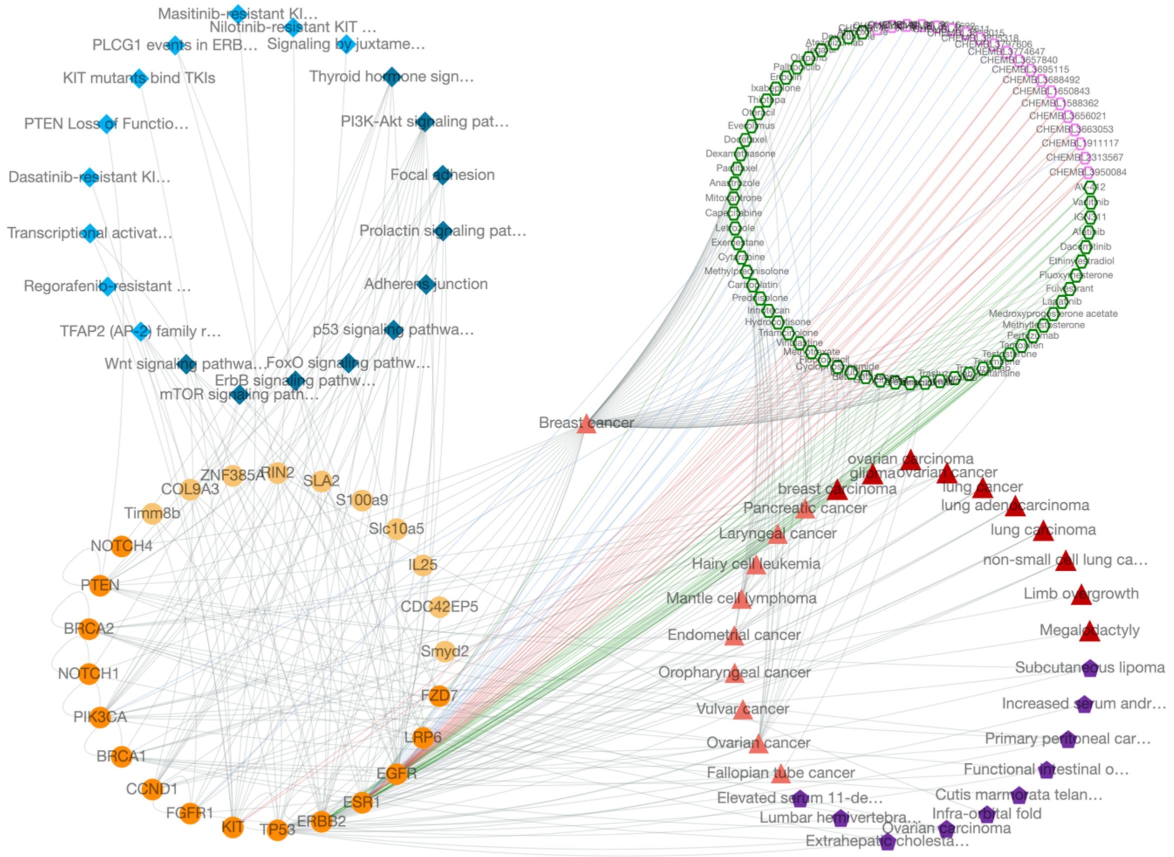
Masitinib-resistant KI…
Nilotinib-resistant KIT …
PLCG1 events in ERB…
Signaling by juxtame…
KIT mutants bind TKIs
Thyroid hormone sign…
PTEN Loss of Functio…
PI3K-Akt signaling pat…
Dasatinib-resistant KI…
Focal adhesion
Transcriptional activat…
Prolactin signaling pat…
Regorafenib-resistant …
Adherens junction
TFAP2 (AP-2) family r…
p53 signaling pathwa…
Wnt signaling pathwa…
FoxO signaling pathw…
ErbB signaling pathw…
mTOR signaling path…
COL9A3
ZNF385A
RIN2
SLA2
S100a9
Timm8b
Slc10a5
NOTCH4
IL25
PTEN
CDC42EP5
BRCA2
Smyd2
NOTCH1
FZD7
PIK3CA
LRP6
BRCA1
EGFR
CCND1
ESR1
FGFR1
KIT
TP53
ERBB2
Breast cancer
ovarian carcinoma
glioma
ovarian cancer
breast carcinoma
lung cancer
Pancreatic cancer
lung adenocarcinoma
Laryngeal cancer
lung carcinoma
Hairy cell leukemia
non-small cell lung ca…
Mantle cell lymphoma
Limb overgrowth
Endometrial cancer
Megalodactyly
Oropharyngeal cancer
Subcutaneous lipoma
Vulvar cancer
Increased serum andr…
Ovarian cancer
Primary peritoneal car…
Fallopian tube cancer
Functional intestinal o…
Elevated serum 11-de…
Cutis marmorata telan…
Lumbar hemivertebra…
Infra-orbital fold
Ovarian carcinoma
Extrahepatic cholesta…
Medroxyprogesterone acetate
Fulvestrant
Dacomitinib
Ethinylestradiol
Fluoxymesterone
Everolimus
Docetaxel
Dexamethasone
Paclitaxel
Anastrozole
Mitoxantrone
Capecitabine
Letrozole
Exemestane
Cytarabine
Methylprednisolone
Carboplatin
Prednisolone
Hydrocortisone
Thiotepa
Ixabepilone
Palbociclib

(b)

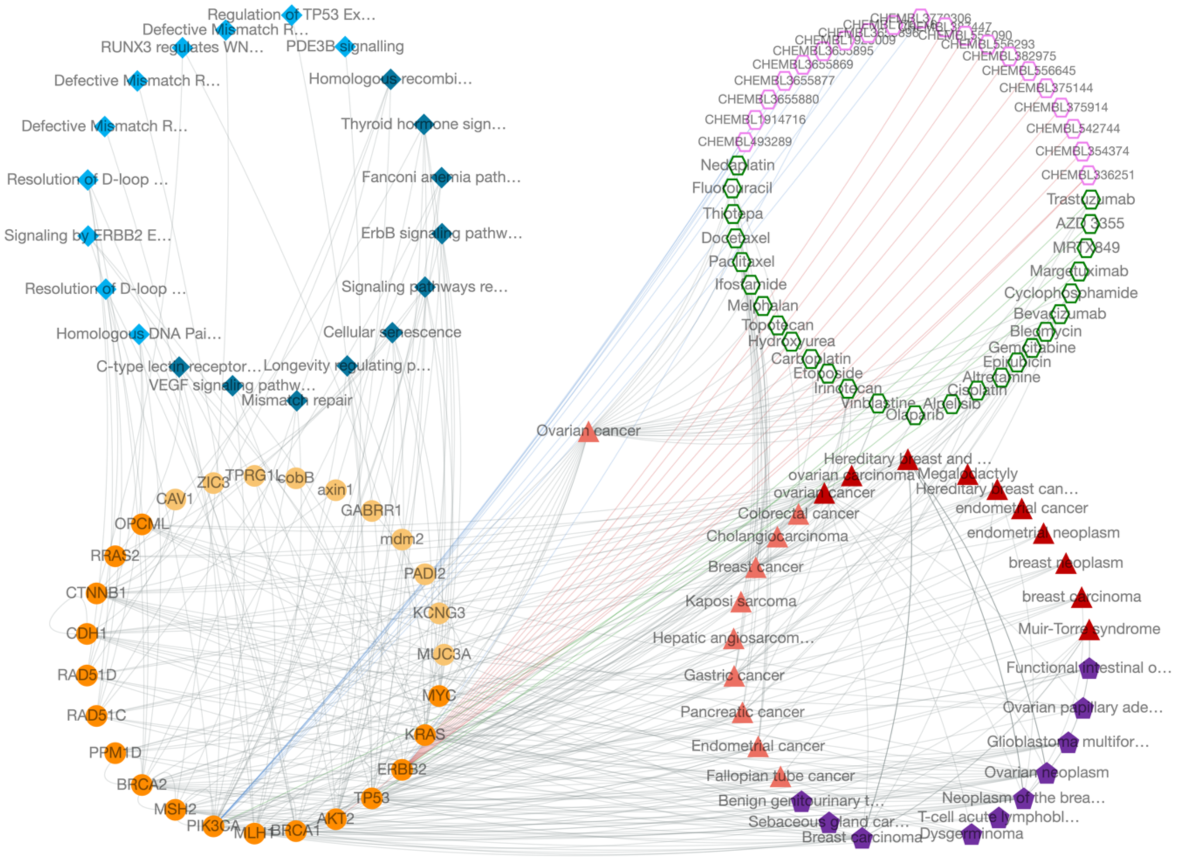
Regulation of TP53 Ex…
Defective Mismatch R…
RUNX3 regulates WN…
PDE3B signalling
Defective Mismatch R…
Homologous recombi…
Defective Mismatch R…
Thyroid hormone sign…
Resolution of D-loop …
Fanconi anemia path…
Signaling by ERBB2 E…
ErbB signaling pathw…
Resolution of D-loop …
Signaling pathways re…
Homologous DNA Pai…
Cellular senescence
C-type lectin receptor…
Longevity regulating p…
VEGF signaling pathw…
Mismatch repair
ZIC3
TPRG1
cobB
CAV1
axin1
OPCML
GABRR1
mdm2
RRAS2
PADI2
CTNNB1
KCNG3
CDH1
MUC3A
RAD51D
MYC
RAD51C
KRAS
PPM1D
ERBB2
BRCA2
TP53
MSH2
AKT2
PIK3CA
MLH1
BRCA1
Ovarian cancer
Hereditary breast and …
ovarian carcinoma
Megalodactyly
ovarian cancer
Hereditary breast can…
Colorectal cancer
endometrial cancer
Cholangiocarcinoma
endometrial neoplasm
Breast cancer
breast neoplasm
Kaposi sarcoma
breast carcinoma
Hepatic angiosarcom…
Muir-Torre syndrome
Gastric cancer
Functional intestinal o…
Pancreatic cancer
Ovarian papillary ade…
Endometrial cancer
Glioblastoma multifor…
Fallopian tube cancer
Ovarian neoplasm
Benign genitourinary t…
Neoplasm of the brea…
Sebaceous gland car…
T-cell acute lymphobl…
Breast carcinoma
Dysgerminoma
Nedaplatin
Fluorouracil
Thiotepa
Docetaxel
Paclitaxel
Ifosfamide
Melphalan
Topotecan
Hydroxyurea
Carboplatin
Etoposide
Irinotecan
Vinblastine
Olaparib
Alpelisib
Cisplatin
Altretamine
Epirubicin
Gemcitabine
Bleomycin
Bevacizumab
Cyclophosphamide
Margetuximab
MRTX849
AZD3355
Trastuzumab
CHEMBL3655869
CHEMBL3655877
CHEMBL3655880
CHEMBL1914716
CHEMBL493289
CHEMBL382975
CHEMBL556645
CHEMBL375144
CHEMBL375914
CHEMBL542744
CHEMBL354374
CHEMBL336251

(c)

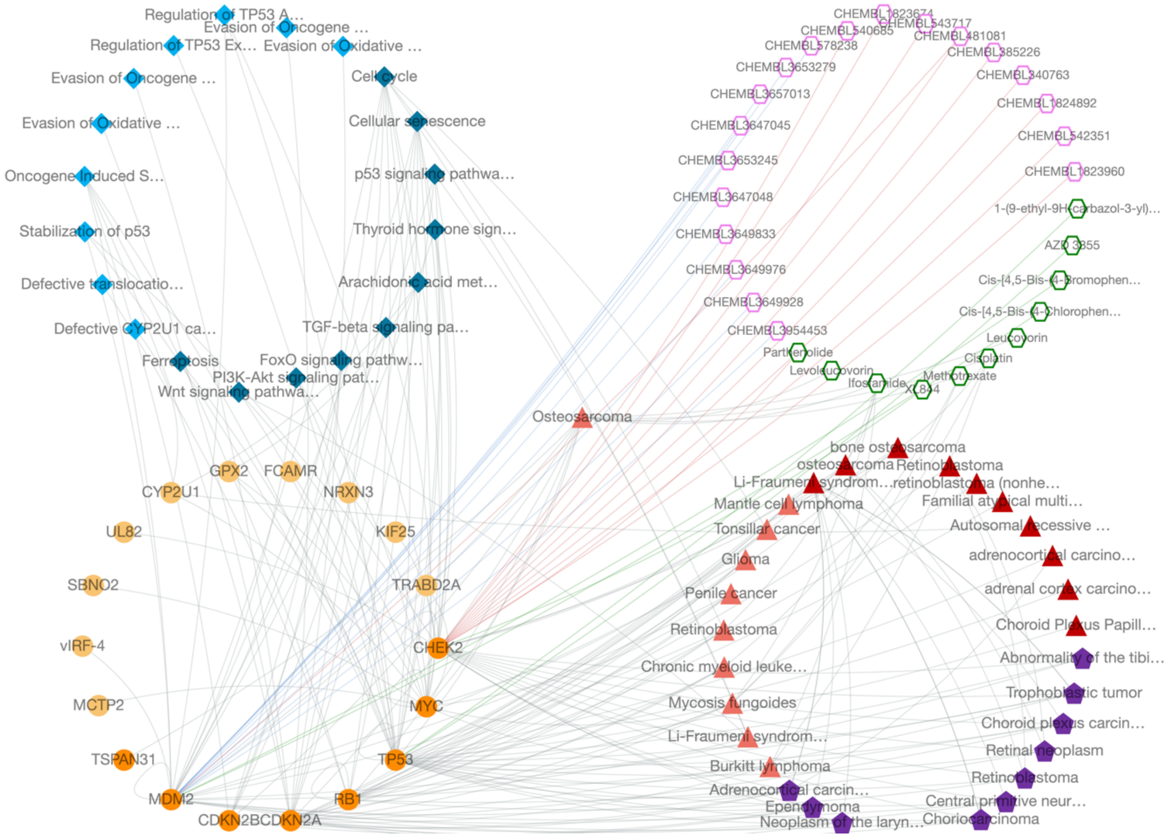

Figure S3. CROssBAR knowledge graph diversity analysis use-case, output KGs of disease queries: **(a)** breast cancer, **(b)** ovarian cancer, and **(c)** osteosarcoma.

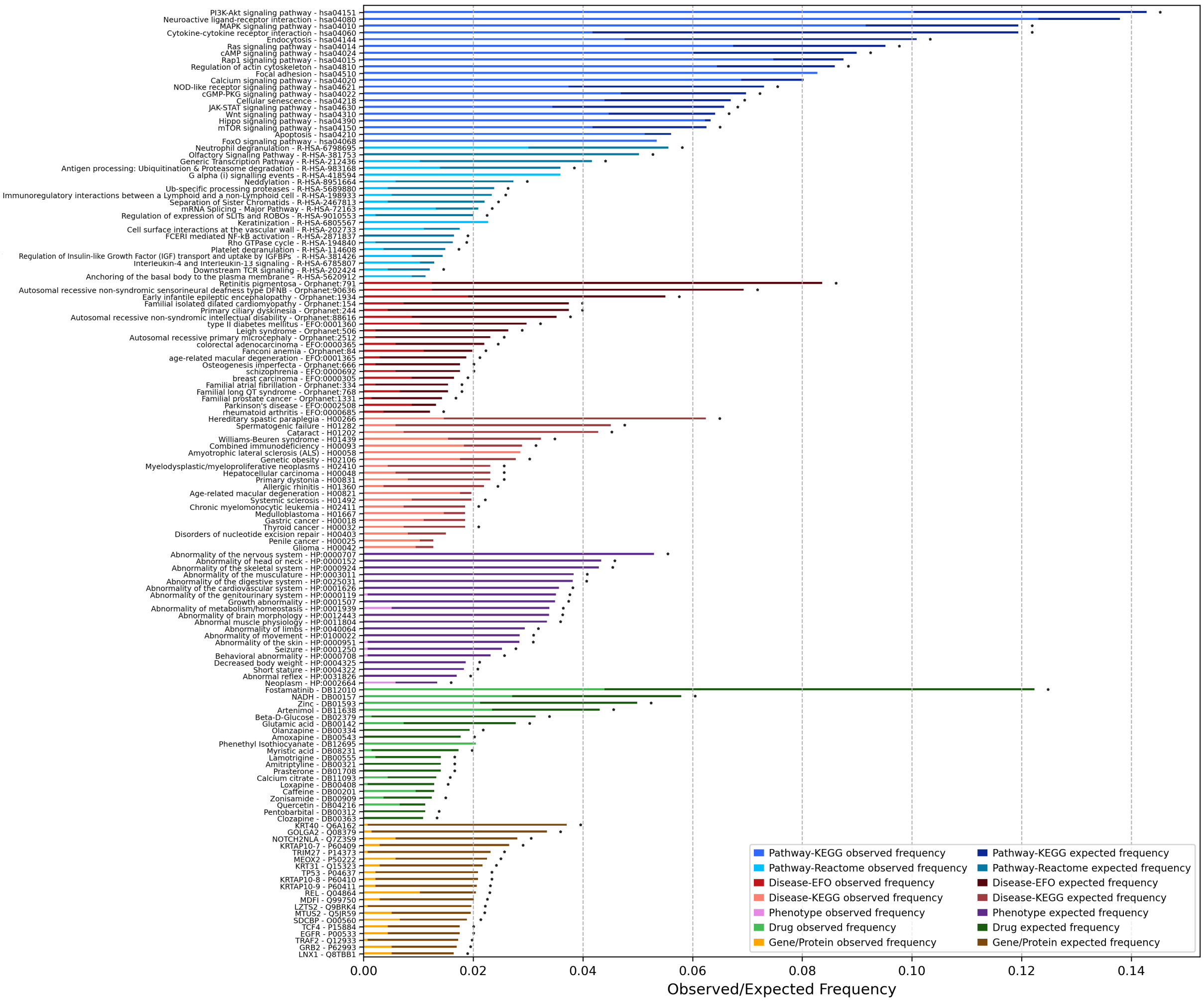

Figure S4. Bar graphs indicating observed and expected frequencies (overlapping bars with different shades of colours) for each of the 140 selected highly connected/hub terms in 1365 CROssBAR knowledge graphs constructed with random term queries. "*" indicate that the corresponding observed frequency is statistically significantly lower compared to the expected frequency according to Fisher's exact test.

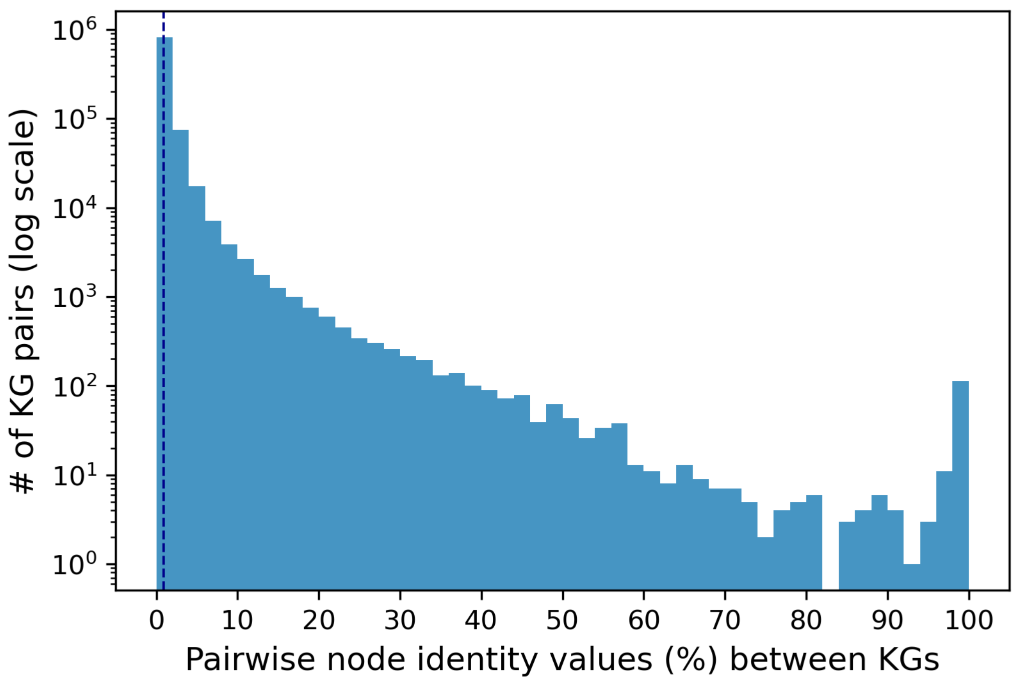

Figure S5. pairwise node identity percentage histogram (in log scale) between all KG pair combinations (930930 measurements) in our 1365 CROssBAR knowledge graphs constructed with random term queries.

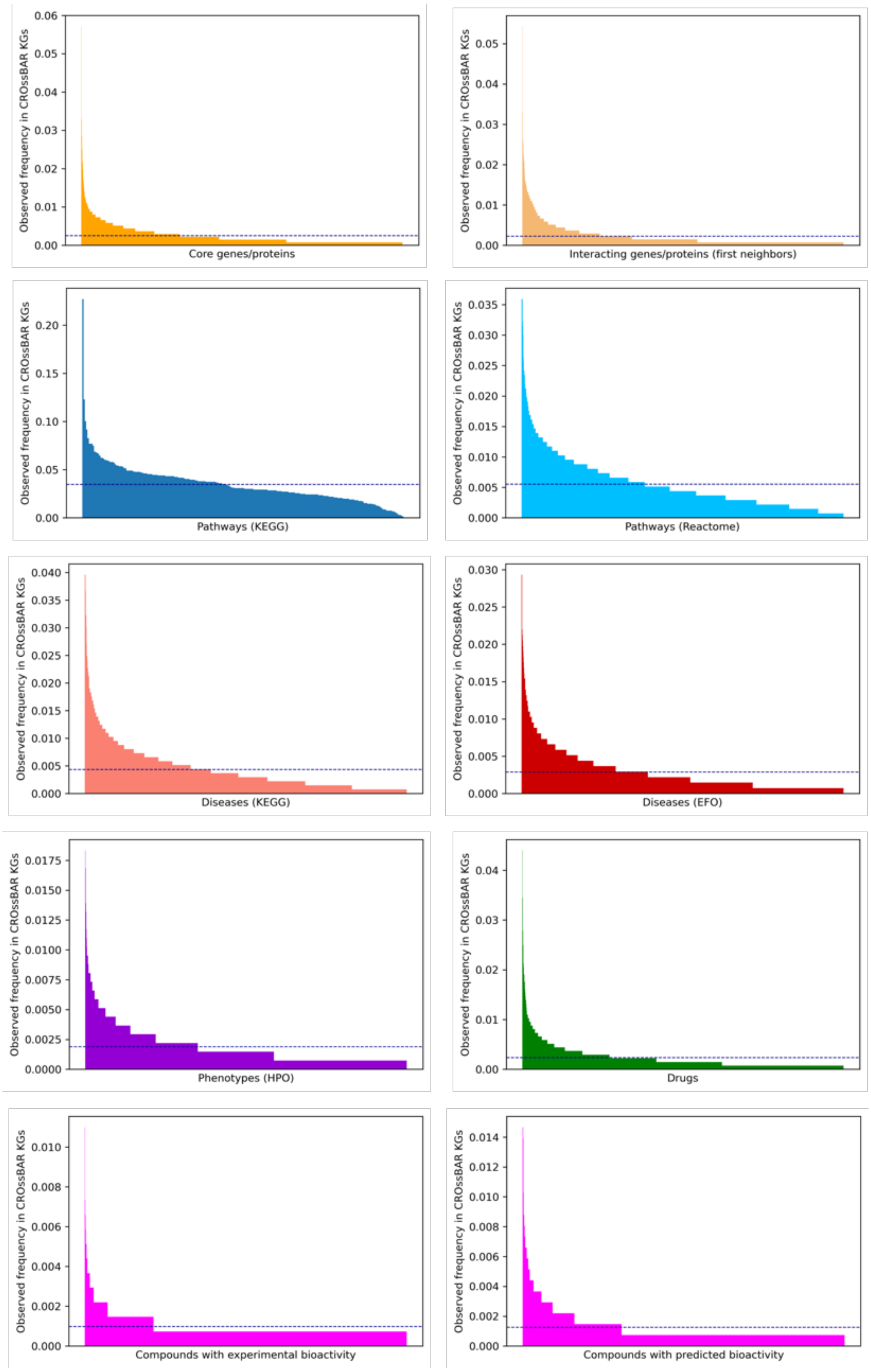

Figure S6. Biological component-wise bar graphs indicating observed frequencies (in vertical axis) of all terms (in horizontal axis by ranking the terms according to decreasing frequencies) that are presented in our 1365 CROssBAR knowledge graphs constructed with random term queries.

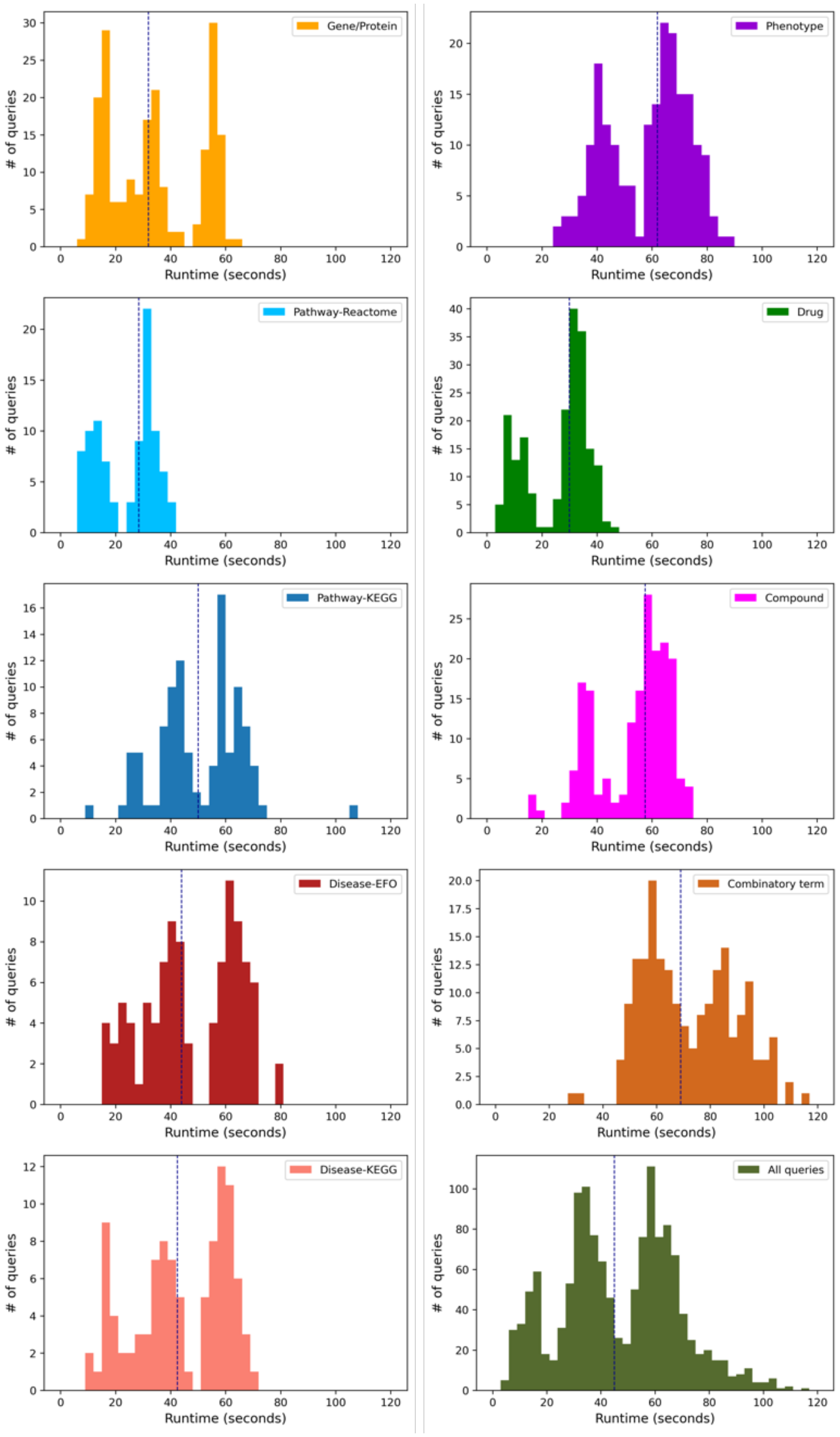

Figure S7. Biological component-wise query runtime histograms of 1365 CROssBAR knowledge graphs constructed with random term queries.

Supplementary Tables

Table S1. CROssBAR database statistics.

High-level data component group	Specific biomedical component	Number of entries
Genes/Proteins (UniProt)	Genes	122,930
	Proteins	608,350
	Proteins w/ PPI	55,689
	PPIs	852,820
	Protein Domain Annotations	949,175
Bioactivities (ChEMBL)	Bioassays	1,358,549
	Compounds	2,086,898
	Bioactivities	17,276,334
	Targets	14,347
Bioactivities (PubChem)	Bioassays	1,067,568
	Compounds	97,351,543
	Substances	252,877,567
	Bioactivities	268,562,338
Drugs (DrugBank)	Drugs	14,315
	Targets	4,867
	DTIs	18,866
Diseases (KEGG+EFO)	EFO Disease Terms	27,415
	EFO Genes (Unique #)	4,272
	EFO Annotations	14,062
	KEGG Disease Terms	1,901
	KEGG Disease Genes (Unique #)	3,710
	KEGG Disease Annotations	6,040
Phenotypes (HPO)	HPO Terms	9,595
	HPO related Genes (Unique #)	4,531
	HPO Annotations	645,861
Pathways (KEGG+Reactome)	KEGG Pathway Terms	337
	KEGG Pathway Genes (Unique #)	8,040
	KEGG Pathway Annotations	32,516
	Reactome Terms	7,254
	Reactome Proteins (Unique #)	43,034
	Reactome Annotations	136,242

Table S2. List of the differentially expressed genes (DEGs) of chloroquine phosphate treated liver cells (Huh7 and Mahlavu), with a p-value cut-off < 0.01.

Cell line: Huh7

Gene name	Log2 fold change	p-value	probe.ID
ABL1	0.183	0.000546	NM_005157.3:3200
ANGPT1	-0.171	0.00108	NM_001146.3:2080
ARID1A	0.203	0.000134	NM_006015.4:5495
ATM	-0.25	0.000887	NM_138292.3:6688
B2M	0.132	0.00608	NM_004048.2:25
BAMBI	-0.171	0.00145	NM_012342.2:832
BAP1	-0.149	0.00279	NM_004656.2:240
BCL2L1	-0.192	1.66E-05	NM_138578.1:1560
BCOR	-0.369	0.00449	NM_001123383.1:1630
BID	0.189	0.00203	NM_197966.1:2095
BNIP3	0.149	0.000208	NM_004052.2:325
BRCA2	0.0707	0.00607	NM_000059.3:115
C19orf40	0.355	3.51E-06	NM_152266.3:376
CAPN2	-0.513	0.00282	NM_001748.4:2085
CASP8	-0.105	0.00304	NM_001228.4:301
CCNA2	-0.0804	0.00201	NM_001237.2:1210
CCNB1	-0.0811	0.00251	NM_031966.2:715
CCNE2	0.218	0.00156	NM_057735.1:50
CDC25A	0.0634	0.00529	NM_001789.2:690
CDC6	-0.0589	0.00767	NM_001254.3:1300
CDH1	0.164	0.00136	NM_004360.2:1230
CDK2	-0.179	0.00541	NM_001798.2:220
CDK4	0.104	0.00765	NM_000075.2:1055
CDKN2D	-0.554	0.0024	NM_001800.3:870
CEBPA	0.137	0.00866	NM_004364.2:1320
COL2A1	0.417	1.56E-05	NM_001844.4:4745
CREB3L3	0.294	0.000332	NM_001271995.1:1050
CREB5	-0.228	0.00118	NM_182898.2:1885
CSF3R	0.675	0.00275	NM_156038.2:90
CUL1	0.111	1.11E-05	NM_003592.2:1487
DAXX	-0.117	0.0084	NM_001350.3:1875
DDIT3	0.5	1.98E-05	NM_004083.4:40
DKK1	-0.16	0.000474	NM_012242.2:75
DNMT1	-0.075	0.00034	NM_001379.2:1495
DUSP4	0.282	0.000107	NM_057158.2:3115
DUSP5	0.128	0.000351	NM_004419.3:675
E2F5	-0.0998	0.00168	NM_001951.3:444
EFNA1	0.285	0.00906	NM_004428.2:650
EIF4EBP1	-0.0695	0.0105	NM_004095.3:363
ENDOG	-0.106	0.00731	NM_004435.2:694
EP300	0.139	0.00345	NM_001429.2:715
EPHA2	-0.208	0.00263	NM_004431.2:1525

Gene name	Log2 fold change	p-value	probe.ID
IL6R	0.558	0.00014	NM_000565.2:993
IL8	0.321	1.32E-05	NM_000584.2:25
ITGA2	-0.115	0.00566	NM_002203.2:475
ITGB8	0.383	8.61E-05	NM_002214.2:2609
JAG1	-0.145	0.00865	NM_000214.2:915
JAK2	0.222	0.0055	NM_004972.2:455
KAT2B	0.229	0.00372	NM_003884.3:1220
KDM5C	0.0422	0.000153	NM_004187.2:1170
KMT2C	0.136	0.00696	NM_170606.2:12505
KMT2D	-0.127	0.000176	NM_003482.3:6070
LFNG	-0.755	0.000944	NM_001040168.1:717
LIFR	0.0762	0.00956	NM_002310.3:2995
MAD2L2	-0.223	6.49E-05	NM_001127325.1:290
MAML2	-0.806	0.00055	NM_032427.1:4125
MAP2K1	-0.0886	0.00458	NM_002755.2:970
MAP3K14	0.506	0.000158	NM_003954.1:620
MAPK8	0.0894	0.00051	NM_002750.2:945
MCM2	-0.148	0.00264	NM_004526.2:2945
MNAT1	0.0794	0.00721	NM_002431.2:975
MSH6	-0.0825	0.00904	NM_000179.1:3525
MYC	0.0783	0.0014	NM_002467.3:1610
NASP	-0.221	0.000635	NM_172164.1:2970
NF1	-0.0983	0.00692	NM_000267.2:1035
NFE2L2	-0.178	0.000167	NM_006164.3:995
NFKBIZ	0.308	0.000801	NM_001005474.1:2030
NOTCH1	0.0941	0.00144	NM_017617.3:735
NPM1	-0.383	0.000825	NM_002520.5:10
NRAS	-0.0928	0.000532	NM_002524.3:877
NTHL1	-0.241	0.00124	NM_002528.5:476
NUMBL	0.364	0.00397	NM_004756.3:591
NUPR1	1.1	0.00249	NM_001042483.1:829
PAK7	-0.28	0.00347	NM_177990.1:615
PDGFA	-0.558	0.00911	NM_002607.5:2460
PDGFC	-0.243	7.72E-06	NM_016205.1:10
PDGFRB	-0.198	0.00372	NM_002609.3:840
PIK3R3	0.216	0.000351	NM_003629.3:1800
PIM1	0.478	0.000563	NM_002648.2:1630
PLAU	0.474	0.000477	NM_002658.2:793
POLD1	-0.394	0.00403	NM_002691.2:2392
POLR2H	-0.126	0.00098	NM_001278698.1:940
PPARG	-0.263	0.000212	NM_015869.3:1035
PPP3CA	-0.319	0.000434	NM_000944.4:3920

EPOR	0.314	0.000218	NM_000121.2:1295
ERBB2	0.127	0.00756	NM_004448.2:2380
ETV1	-0.145	0.00276	NM_004956.4:1719
ETV4	-0.153	0.0027	NM_001079675.1:1535
FANCB	0.423	0.00158	NM_152633.2:2470
FANCE	-0.466	0.000715	NM_021922.2:1275
FANCG	-0.201	0.00355	NM_004629.1:1900
FGFR2	0.503	0.00163	NM_000141.4:647
FGFR3	-0.0863	0.0065	NM_022965.2:3170
FLNA	-0.362	0.000317	NM_001456.3:7335
FN1	-0.0797	0.000226	NM_212482.1:1776
FOS	1.05	1.68E-05	NM_005252.2:1475
FST	-0.141	0.000331	NM_006350.2:575
FUT8	0.247	0.000113	NM_004480.4:2841
GADD45A	0.345	7.75E-05	NM_001924.2:865
GNA11	-0.164	0.00319	NM_002067.1:555
GNAQ	-0.165	0.00862	NM_002072.2:1100
GNAS	0.0653	0.00911	NM_080425.1:1910
GNG12	-0.151	0.000221	NM_018841.3:245
GNG4	-0.279	0.00275	NM_004485.2:215
GNG7	0.24	0.00458	NM_052847.1:3920
GTF2H3	0.0392	0.00241	NM_001516.3:70
H3F3A	-0.108	0.000457	NM_002107.3:190
HDAC1	-0.183	0.000104	NM_004964.2:785
HIST1H3H	-0.0973	7.93E-05	NM_003536.2:355
HMGA1	-0.183	0.00252	NM_145904.1:871
HMGA2	-0.111	0.00275	NM_003484.1:328
HOXA9	0.289	0.00278	NM_152739.3:1015
HSPA2	-0.347	0.00214	NM_021979.3:2095
ID2	0.0544	0.00489	NM_002166.4:505
IDH1	0.2	0.00181	NM_005896.2:105
IGFBP3	-0.351	0.000131	NM_000598.4:1255
IKBKG	0.379	8.18E-05	NM_003639.2:470
IL1R1	0.362	0.000269	NM_000877.2:4295

PRKCA	-0.123	0.000426	NM_002737.2:5560
PRKDC	-0.075	0.00354	NM_006904.6:12750
PRKX	-0.341	0.000311	NM_005044.1:2590
PRLR	-0.184	0.000673	NM_001204318.1:563
PROM1	-0.238	0.00121	NM_006017.1:925
PTTG2	-0.127	0.000152	NM_006607.2:5
RAD50	0.147	0.00924	NM_005732.2:5397
RAF1	0.0629	0.00789	NM_002880.2:1990
RELA	-0.16	0.00378	NM_021975.3:1990
RELN	0.222	0.000153	NM_005045.2:345
RHOA	-0.212	0.00033	NM_001664.2:1230
RUNX1	0.416	0.0044	NM_001754.4:635
SETD2	0.188	0.000112	NM_014159.6:6160
SHC2	0.331	0.001	NM_012435.2:698
SMAD2	0.145	0.000271	NM_001003652.1:4500
SMAD4	0.0814	0.00388	NM_005359.3:1370
SMARCB1	-0.118	0.0036	NM_003073.3:1060
SOCS1	-0.389	2.06E-05	NM_003745.1:1025
SPRY1	-0.434	0.000495	NM_005841.1:810
SPRY2	-0.107	0.00232	NM_005842.2:85
STK11	-0.2	0.00263	NM_000455.4:2060
TBL1XR1	0.0898	0.00555	NM_024665.4:915
TCF7L1	0.3	0.00317	NM_031283.1:2215
TFDP1	-0.134	0.000719	NM_007111.4:1826
TGFB1	-0.361	0.00153	NM_000660.3:1260
TGFBR2	-0.121	0.000536	NM_001024847.1:1760
THBS4	0.25	0.0078	NM_003248.3:985
TNFAIP3	0.488	9.29E-05	NM_006290.2:260
TP53	-0.154	0.000392	NM_000546.2:1330
TSC1	0.322	0.00115	NM_000368.3:100
UBE2T	0.0624	0.00199	NM_014176.3:595
VEGFA	0.266	0.000124	NM_001025366.1:1325
WIF1	0.883	3.42E-05	NM_007191.2:765
XRCC4	0.209	0.000844	NM_003401.3:772

Cell line: Mahlavu

Gene name	Log2 fold change	P-value	probe.ID
ABL1	0.196	0.000142	NM_005157.3:3200
ALKBH2	0.23	0.00364	NM_001001655.2:907
ALKBH3	0.0718	0.00275	NM_139178.3:690
ANGPT1	0.183	0.00135	NM_001146.3:2080
APC	-0.24	0.000634	NM_000038.3:6850
ARID1A	-0.284	0.00124	NM_006015.4:5495
ARID2	-0.0882	0.0102	NM_152641.2:3355
ASXL1	-0.0805	0.000481	NM_001164603.1:472
ATR	-0.248	0.00643	NM_001184.2:565
B2M	0.169	0.0046	NM_004048.2:25

Gene name	Log2 fold change	P-value	probe.ID
LAMB3	0.343	0.00263	NM_000228.2:695
LEF1	0.118	0.00617	NM_016269.3:1165
LEPR	-0.397	0.000642	NM_001003679.1:2000
LIF	0.779	0.00119	NM_002309.3:1240
LTBP1	0.593	0.000799	NM_000627.3:4124
MAD2L2	-0.0859	0.00528	NM_001127325.1:290
MAML2	0.227	0.00547	NM_032427.1:4125
MAP2K1	0.216	0.00127	NM_002755.2:970
MAP2K4	-0.247	0.000241	NM_003010.2:2830
MAP2K6	-0.287	0.000167	NM_002758.3:555

BAP1	-0.0531	0.00866	NM_004656.2:240	MAP3K1	-0.0658	0.00628	NM_005921.1:2525
BCL2L1	0.0837	0.00122	NM_138578.1:1560	MAPK1	-0.0858	0.00232	NM_138957.2:430
BNIP3	0.409	4.03E-06	NM_004052.2:325	MAPK12	-0.45	0.000533	NM_002969.3:425
BRAF	-0.123	0.00297	NM_004333.3:565	MAPK8	0.096	0.00451	NM_002750.2:945
BRCA1	0.186	0.00578	NM_007305.2:1275	MCM2	-0.0885	0.00354	NM_004526.2:2945
BRCA2	0.206	0.00445	NM_000059.3:115	MCM5	-0.254	0.00695	NM_006739.3:1580
BRIP1	0.43	0.000703	NM_032043.1:1130	MCM7	0.0984	0.00256	NM_182776.1:1325
CASP7	0.25	0.00341	NM_001227.3:915	MED12	-0.67	0.000414	NM_005120.2:375
CASP8	0.0783	0.0053	NM_001228.4:301	MET	0.0583	0.000913	NM_000245.2:405
CBL	-0.211	0.000837	NM_005188.2:7485	MGMT	0.149	0.00182	NM_002412.3:323
CCNB1	-0.132	0.000125	NM_031966.2:715	MLLT3	-0.553	0.00496	NM_004529.2:1480
CCNB3	-0.0904	0.000954	NM_033671.1:35	MSH6	-0.17	4.64E-05	NM_000179.1:3525
CCND1	0.452	6.21E-05	NM_053056.2:690	MUTYH	-0.41	0.000465	NM_012222.2:412
CDC25B	-0.184	7.60E-05	NM_021873.2:3045	NBN	-0.0622	0.00497	NM_001024688.1:1105
CDC25C	-0.233	0.00309	NM_001790.2:1055	NCOR1	0.0934	0.00145	NM_006311.3:1390
CDC6	0.233	0.000373	NM_001254.3:1300	NF1	-0.062	0.00281	NM_000267.2:1035
CDK2	0.324	0.00183	NM_001798.2:220	NGF	0.411	6.58E-05	NM_002506.2:100
CDK4	0.128	7.50E-05	NM_000075.2:1055	NOG	-0.182	0.00277	NM_005450.4:1543
CDKN1A	0.824	1.87E-05	NM_000389.2:1975	NOTCH2	0.0875	0.00386	NM_024408.3:2842
CDKN1B	-0.24	0.000518	NM_004064.2:365	NPM1	-0.216	0.00405	NM_002520.5:10
CDKN2C	-0.264	0.00369	NM_001262.2:1295	NRAS	-0.152	1.20E-05	NM_002524.3:877
CDKN2D	-0.234	0.000906	NM_001800.3:870	NTHL1	-0.355	0.000495	NM_002528.5:476
CHEK2	0.121	0.00526	NM_007194.3:140	NUMBL	-0.184	0.00883	NM_004756.3:591
COL5A1	0.0662	0.00984	NM_000093.3:6345	NUPR1	0.63	3.53E-05	NM_001042483.1:829
CREB3L1	-0.113	0.000205	NM_052854.1:195	PBX1	-0.437	0.000271	NM_002585.2:368
CTNNB1	-0.0484	0.00303	NM_001904.3:2265	PDGFC	0.146	0.00113	NM_016205.1:10
CYLD	0.192	3.54E-05	NM_015247.1:2890	PIK3CA	-0.146	0.003	NM_006218.2:2445
DDB2	0.362	0.000916	NM_000107.1:840	PIK3R1	0.172	0.00185	NM_181504.2:1105
DDIT3	0.365	2.00E-05	NM_004083.4:40	PIK3R2	-0.125	0.000396	NM_005027.2:3100
DDIT4	0.564	0.000469	NM_019058.2:85	PIM1	0.341	0.00226	NM_002648.2:1630
DUSP4	0.813	1.73E-05	NM_057158.2:3115	PKMYT1	-0.135	0.00766	NM_004203.3:780
DUSP5	0.486	5.60E-06	NM_004419.3:675	PLA2G3	-0.27	0.00939	NM_015715.3:2415
DUSP6	0.836	0.000328	NM_001946.2:1535	PLA2G4C	1.05	0.000181	NM_003706.2:2310
E2F1	-0.083	0.00364	NM_005225.1:935	PLAT	0.957	3.73E-07	NM_000931.2:1334
EFNA5	0.395	0.000258	NM_001962.2:5035	PLAU	-0.0725	0.00152	NM_002658.2:793
EGFR	0.152	0.000696	NM_201282.1:360	PLCB1	0.459	0.000508	NM_182734.1:170
EIF4EBP1	0.172	8.06E-06	NM_004095.3:363	PLCE1	0.175	0.0037	NM_001165979.1:392
ENDOG	-0.137	5.00E-04	NM_004435.2:694	PLD1	0.24	0.00613	NM_002662.3:1265
EP300	-0.104	0.00266	NM_001429.2:715	PML	0.425	9.64E-06	NM_002675.3:281
EPHA2	0.0849	0.0104	NM_004431.2:1525	POLD4	0.172	0.00716	NM_021173.2:470
ERCC6	0.0841	0.00483	NM_000124.2:3235	POLR2H	0.31	7.13E-05	NM_001278698.1:940
ETS2	0.107	0.000263	NM_005239.4:1175	POLR2J	-0.385	0.00293	NM_006234.4:618
ETV4	0.693	0.00118	NM_001079675.1:1535	PPARGC1A	0.338	0.000103	NM_013261.3:1505
FANCB	0.16	0.00506	NM_152633.2:2470	PPP2R1A	-0.107	0.00886	NM_014225.3:1440
FANCE	-0.265	0.000251	NM_021922.2:1275	PPP3CA	-0.175	0.00507	NM_000944.4:3920
FANCG	-0.189	0.00447	NM_004629.1:1900	PPP3CC	0.434	0.000341	NM_005605.3:1460
FAS	0.277	0.00461	NM_152876.1:1740	PRKACA	-0.102	0.000545	NM_002730.3:400

FBXW7	-0.136	0.00807	NM_018315.4:1480
FEN1	0.13	0.000721	NM_004111.4:425
FGF1	0.893	4.04E-06	NM_033137.1:315
FGF2	-0.105	0.000208	NM_002006.4:620
FGFR1	-0.177	0.000794	NM_015850.2:1335
FN1	0.436	8.99E-08	NM_212482.1:1776
FOS	1.17	9.84E-07	NM_005252.2:1475
FOSL1	0.187	0.000545	NM_005438.2:280
FST	0.605	0.00129	NM_006350.2:575
FUBP1	-0.298	6.65E-05	NM_003902.3:820
FUT8	0.138	0.00723	NM_004480.4:2841
FZD2	-0.445	8.59E-06	NM_001466.2:845
FZD7	0.427	0.000902	NM_003507.1:1890
GADD45A	0.356	4.54E-09	NM_001924.2:865
GATA2	0.313	0.000546	NM_032638.3:1495
GNA11	-0.0539	0.00818	NM_002067.1:555
GNAS	0.0315	0.000164	NM_080425.1:1910
GRB2	-0.0778	0.00643	NM_002086.4:412
H3F3A	-0.114	6.11E-05	NM_002107.3:190
H3F3C	0.0729	0.00462	NM_001013699.2:829
HDAC1	-0.0938	0.0031	NM_004964.2:785
HDAC10	-0.414	0.00185	NM_032019.5:932
HDAC5	-0.155	0.00673	NM_005474.4:3160
HDAC6	-0.106	0.00736	NM_006044.2:536
HES1	0.573	2.50E-05	NM_005524.2:860
HIST1H3B	-0.36	1.10E-05	NM_003537.3:335
HIST1H3H	-0.768	5.07E-07	NM_003536.2:355
HOXA10	-0.167	0.00323	NM_018951.3:1503
HSP90B1	-0.232	7.47E-06	NM_003299.1:160
HSPA1A	-0.627	0.00078	NM_005345.5:98
HSPB1	-0.297	2.80E-07	NM_001540.3:374
ID1	-0.329	0.00199	NM_002165.2:345
ID2	-0.293	0.000322	NM_002166.4:505
IDH1	0.372	0.000114	NM_005896.2:105
IDH2	0.296	0.000212	NM_002168.2:944
IGFBP3	-0.13	0.00114	NM_000598.4:1255
IL1RAP	0.227	0.00317	NM_002182.2:460
IL6	0.942	2.98E-05	NM_000600.1:220
IL6R	0.113	0.00351	NM_000565.2:993
IL7R	0.278	8.56E-05	NM_002185.2:1610
IRAK2	0.368	0.00105	NM_001570.3:1285
ITGA2	-0.213	0.000358	NM_002203.2:475
ITGB3	0.838	1.70E-06	NM_000212.2:4485
JAG2	-0.291	0.000984	NM_145159.1:4225
JAK1	-0.186	0.00128	NM_002227.1:285
JUN	0.457	2.58E-06	NM_002228.3:140
KAT2B	0.169	0.00135	NM_003884.3:1220
PRKAR1B	0.234	0.000221	NM_001164759.1:1112
PRKAR2B	-0.205	0.00885	NM_002736.2:1350
PTPN11	0.209	3.37E-06	NM_002834.3:1480
PTPRR	0.377	0.000247	NM_001207015.1:1652
PTTG2	-0.1	0.000445	NM_006607.2:5
RAD21	-0.112	3.10E-05	NM_006265.2:1080
RAD50	0.349	5.04E-07	NM_005732.2:5397
RB1	-0.0768	0.0012	NM_000321.1:2110
RFC3	0.0397	0.0093	NM_002915.3:740
RHOA	-0.142	0.000195	NM_001664.2:1230
RPS6KA5	0.452	2.72E-05	NM_004755.2:855
RUNX1	-0.247	0.000278	NM_001754.4:635
SETD2	-0.182	0.00134	NM_014159.6:6160
SF3B1	-0.0852	0.000348	NM_001005526.1:0
SIN3A	-0.397	0.000405	NM_015477.1:1605
SKP1	-0.11	2.50E-05	NM_170679.2:630
SMAD2	-0.143	0.000164	NM_001003652.1:4500
SMAD4	0.186	0.00874	NM_005359.3:1370
SMARCA4	-0.153	0.000294	NM_003072.3:5400
SMARCB1	-0.202	3.73E-06	NM_003073.3:1060
SOCS1	0.784	1.49E-05	NM_003745.1:1025
SOS1	-0.109	0.00599	NM_005633.2:1635
SOS2	0.314	0.000529	NM_006939.2:3845
SPRY2	0.337	0.00054	NM_005842.2:85
SPRY4	0.346	0.0064	NM_030964.3:1900
SRSF2	-0.202	0.00094	NM_003016.3:312
STAG2	-0.153	0.000859	NM_001042749.1:4040
STAT1	0.566	8.49E-06	NM_007315.2:205
STK11	-0.146	0.000781	NM_000455.4:2060
STMN1	-0.175	0.00217	NM_203401.1:478
SUV39H2	-0.175	0.00179	NM_024670.3:2035
TGFB1	-0.219	1.51E-05	NM_000660.3:1260
TGFB2	0.274	2.37E-05	NM_003238.2:1125
TGFBR2	0.224	0.000309	NM_001024847.1:1760
TIAM1	0.831	0.00205	NM_003253.2:5620
TLR4	0.531	0.00021	NM_138554.2:2570
TNC	0.235	0.000118	NM_002160.3:4
TNFAIP3	0.709	0.00011	NM_006290.2:260
TNFRSF10B	0.436	4.46E-06	NM_003842.3:565
TNFRSF10D	-0.45	0.00692	NM_003840.3:2380
TP53	-0.102	0.00227	NM_000546.2:1330
TSC1	0.243	0.00108	NM_000368.3:100
TSLP	0.542	0.000111	NM_033035.4:899
TTK	-0.138	0.0022	NM_003318.3:1200
UBE2T	0.12	0.00239	NM_014176.3:595
VEGFA	0.622	4.65E-06	NM_001025366.1:1325
VHL	0.121	9.16E-06	NM_000551.2:1280

KDM6A	0.195	0.00201	NM_021140.2:2590
KITLG	0.313	0.000525	NM_003994.4:1155
KLF4	0.608	4.89E-06	NM_004235.4:1980
LAMA1	0.212	0.000991	NM_005559.2:5230
LAMA3	0.319	9.68E-05	NM_000227.3:4260
LAMA5	-0.413	0.01	NM_005560.3:787

WEE1	0.155	0.000653	NM_003390.3:1225
WT1	-0.449	0.00213	NM_000378.3:2160
XPA	-0.182	0.000662	NM_000380.3:265
ZAK	-0.13	0.00181	NM_016653.2:995
ZIC2	0.447	0.000131	NM_007129.2:1849

Table S3. Significantly enriched KEGG signalling pathways of chloroquine phosphate treated liver cells (Huh7 and Mahlavu). Only the pathways that are reported to be enriched in both Enrichr (9) and Webgestalt (10) analysis are given. Significance values and scores are obtained from Enrichr.

Cell-line	Term	Odds Ratio	Combined Score	P-value	Adjusted P-value
Huh7	PI3K-Akt signaling pathway	15.611	1352.847	2.31E-38	3.56E-36
	MAPK signaling pathway	15.165	1040.176	1.63E-30	1.67E-28
	Cell cycle	26.528	1718.160	7.44E-29	5.73E-27
	Ras signaling pathway	14.179	687.902	8.50E-22	2.01E-20
	Apoptosis	17.483	716.143	1.62E-18	2.77E-17
	FoxO signaling pathway	16.946	613.216	1.92E-16	2.47E-15
	TGF-beta signaling pathway	19.006	559.205	1.67E-13	1.83E-12
	Th17 cell differentiation	15.986	433.755	1.65E-12	1.54E-11
	p53 signaling pathway	20.102	516.678	6.88E-12	5.05E-11
	Longevity regulating pathway	15.480	383.006	1.80E-11	1.18E-10
	TNF signaling pathway	14.354	342.161	4.44E-11	2.74E-10
	T cell receptor signaling pathway	14.330	314.235	3.00E-10	1.74E-09
	Gap junction	14.952	305.973	1.30E-09	7.01E-09
	Toll-like receptor signaling pathway	8.856	98.595	1.46E-05	4.33E-05
	cGMP-PKG signaling pathway	6.341	64.137	4.05E-05	1.13E-04
	Cytokine-cytokine receptor interaction	3.580	22.484	0.002	0.004
Mahlavu	MAPK signaling pathway	14.860	1427.261	1.93E-42	2.97E-40
	PI3K-Akt signaling pathway	12.641	1137.673	8.20E-40	6.31E-38
	Ras signaling pathway	13.384	855.224	1.77E-28	2.73E-27
	Focal adhesion	11.932	547.317	1.20E-20	1.09E-19
	JAK-STAT signaling pathway	12.402	493.162	5.38E-18	3.60E-17
	TNF signaling pathway	15.774	617.099	1.02E-17	6.71E-17
	Apoptosis	12.134	413.198	1.63E-15	9.10E-15
	HIF-1 signaling pathway	14.612	465.393	1.47E-14	7.43E-14
	TGF-beta signaling pathway	15.221	465.149	5.34E-14	2.49E-13
	Toll-like receptor signaling pathway	13.172	373.438	4.87E-13	2.14E-12
	Oxytocin signaling pathway	8.953	203.071	1.41E-10	5.30E-10
	Cholinergic synapse	8.969	152.336	4.21E-08	1.32E-07
	IL-17 signaling pathway	9.820	161.257	7.38E-08	2.25E-07
	NOD-like receptor signaling pathway	6.670	108.520	8.59E-08	2.59E-07
	Necroptosis	5.637	63.755	1.23E-05	3.07E-05
	Circadian entrainment	5.649	41.080	0.001	0.001
	Renin secretion	6.618	46.006	0.001	0.002
	Endocytosis	2.994	15.531	0.006	0.011

Table S4. The list of 36 genes/proteins shared between the CROssBAR COVID-19 knowledge graph and the differentially expressed genes of the chloroquine phosphate treated liver cells (Huh7 and Mahlavu cell-lines) found using the NanoString platform.

Gene name	Protein accession	Protein name
ATR	Q13535	Serine/threonine-protein kinase ATR
B2M	P61769	Beta-2-microglobulin
BAP1	Q92560	Ubiquitin carboxyl-terminal hydrolase BAP1
BCL2L1	Q07817	Bcl-2-like protein 1
CCNB1	P14635	G2/mitotic-specific cyclin-B1
CCND1	P24385	G1/S-specific cyclin-D1
CDK4	P11802	Cyclin-dependent kinase 4
CDKN1B	P46527	Cyclin-dependent kinase inhibitor 1B
CHEK2	O96017	Serine/threonine-protein kinase Chk2
CUL1	Q13616	Cullin-1
DNMT1	P26358	DNA (cytosine-5)-methyltransferase 1
EGFR	P00533	Epidermal growth factor receptor
EP300	Q09472	Histone acetyltransferase p300
FN1	P02751	Fibronectin
HSPA1A	P0DMV8	Heat shock 70 kDa protein 1A
IKBKG	Q9Y6K9	NF-kappa-B essential modulator
IL6	P05231	Interleukin-6
IL6R	P08887	Interleukin-6 receptor subunit alpha
JUN	P05412	Transcription factor AP-1
LIF	P15018	Leukemia inhibitory factor
MNAT1	P51948	CDK-activating kinase assembly factor MAT1
NPM1	P06748	Nucleophosmin
PLAT	P00750	Tissue-type plasminogen activator
PRKACA	P17612	cAMP-dependent protein kinase catalytic subunit alpha
PRKAR2B	P31323	cAMP-dependent protein kinase type II-beta regulatory subunit
RB1	P06400	Retinoblastoma-associated protein
RHOA	P61586	Transforming protein RhoA
RUNX1	Q01196	Runt-related transcription factor 1
SMAD4	Q13485	Mothers against decapentaplegic homolog 4
STAT1	P42224	Signal transducer and activator of transcription 1-alpha/beta
TGFBR2	P37173	TGF-beta receptor type-2
TIAM1	Q13009	T-lymphoma invasion and metastasis-inducing protein 1
TP53	P04637	Cellular tumor antigen p53
VEGFA	P15692	Vascular endothelial growth factor A
VHL	P40337	von Hippel-Lindau disease tumor suppressor
XPA	P23025	DNA repair protein complementing XP-A cells

Table S5. CROssBAR COVID-19 large-scale and simplified knowledge graphs node and edge statistics.

**Large-scale COVID-19 KG**	
**Node Type**	**Node Stats**
Human genes/proteins	746
SARS-CoV-1 genes/proteins	15
SARS-CoV-2 genes/proteins	17
Drugs	158
Compounds	167
KEGG Pathways	32
Reactome Pathways	68
Phenotype terms (HPO)	43
KEGG Diseases	19
EFO Diseases	22
Organism	2
**TOTAL**	**1289**
**Edge Type**	**Edge Stats**
PPIs	1674
Approved/investigational DTIs*	120
Bioassay-based exp. DTIs (ChEMBL)	382
Predicted DTIs	508
Gene/protein-KEGG Pathway assoc.**	557
Gene/protein-Reactome Pathway assoc.	776
Gene/protein-KEGG Disease assoc.	67
Gene/protein-EFO Disease assoc.	53
Gene/protein-HPO assoc.	2427
HPO-EFO Disease assoc.	56
Drug-KEGG Disease Indications	14
Drug-COVID-19 Disease Indications	62
KEGG Pathway-Disease modulations	3
Orthology relations between SARS-CoVs	12
Organism-gene/protein relations	32
**TOTAL**	**6743**

**Simplified COVID-19 KG**	
**Node_Type**	**Node Stats**
Human genes/proteins	63
SARS-CoV-1 genes/proteins	15
SARS-CoV-2 genes/proteins	17
Drugs	140
Compounds	92
KEGG Pathways	22
Reactome Pathways	43
Phenotype terms (HPO)	27
KEGG Diseases	4
EFO Diseases	9
Organism	3
**TOTAL**	**435**
**Edge_Type**	**Edge Stats**
PPIs	166
Approved/investigational DTIs	36
Bioassay-based exp. DTIs (ChEMBL)	147
Predicted DTIs	16
Gene/protein-KEGG Pathway assoc.	119
Gene/protein-Reactome Pathway assoc.	150
Gene/protein-KEGG Disease assoc.	9
Gene/protein-EFO Disease assoc.	14
Gene/protein-HPO assoc.	188
HPO-EFO Disease assoc.	41
Drug-KEGG Disease Indications	2
Drug-COVID-19 Disease Indications	62
KEGG Pathway-Disease modulations	4
Orthology relations between SARS-CoVs	12
Organism-gene/protein relations	95
**TOTAL**	**1061**

*DTIs: drug/compound-target interactions, **assoc.: associations

Table S6. Literature based information for new potential COVID-19 based repurposing of CROssBAR COVID-19 knowledge graph drugs.

Drug Name	DrugBank id	Description	Prediction Source *	Clinical trial Id	Current state **
Cyclosporine	DB00091	calcineurin inhibitor	Enrichment (DrugBank/ ChEMBL) & Prediction (DEEPScreen)	NCT04392531	Phase 4
Interferon beta-1a	DB00060	immunomodulator	Enrichment (DrugBank)	NCT02735707	Phase 4
Tocilizumab	DB06273	IL-6 inhibitor	Enrichment (DrugBank)	NCT04377750	Phase 4
Tenecteplase	DB00031	fibrin-specific tissue-plasminogen activator	Enrichment (DrugBank)	NCT04558125	Phase 4
Ascorbic acid (Vitamin C)	DB00126	water-soluble vitamin	Enrichment (DrugBank) & Prediction (DEEPScreen)	NCT04401150	Phase 3
Ixekizumab	DB11569	IL-17A inhibitor	Enrichment (DrugBank)	NCT04724629	Phase 3
Epigallocatechin gallate	DB12116	phenolic antioxidant	Enrichment (ChEMBL) & Prediction (DEEPScreen)	NCT04446065	Phase 2-3
Vazegepant	DB15688	calcitonin gene-related peptide (CGRP) receptor antagonist	Prediction (DEEPScreen)	NCT04346615	Phase 2-3
Simvastatin	DB00641	HMG-CoA reductase inhibitor	Enrichment (DrugBank) & Prediction (DEEPScreen)	NCT04348695	Phase 2
Amlodipine	DB00381	calcium channel blocker	Prediction (MDeePred / DEEPScreen)	-	*in vitro* & *in vivo* studies (1,2)
Lifitegrast	DB11611	integrin antagonist	Enrichment (DrugBank)	-	*In silico* & *in vitro* studies (4,5)
Artenimol	DB11638	artemisinin derivative and antimalarial agent	Enrichment (DrugBank) & Prediction (DEEPScreen)	-	*In silico* study (3)
Becatecarin	DB06362	diethylaminoethyl analogue of rebeccamycin	Prediction (MDeePred / DEEPScreen)	-	*In silico* study (7)
Amcinonide	DB00288	corticosteroid	Prediction (MDeePred)	-	*In silico* study (6)
Rocaglamide	DB15495	eIF4A inhibitor	Enrichment (DrugBank) & Prediction (DEEPScreen)	-	-
Didesmethyl rocaglamide	DB15496	eIF4A inhibitor	Enrichment (DrugBank)	-	-

* The source of the drug prediction as either enrichment analysis or DL prediction (prediction of our deep learning-based tools).
** Some of these drugs have multiple clinical trials concerning COVID-19. In these cases, the one with the latest phase is given.

Table S7. Statistics regarding the presence of 140 selected highly connected/hub terms in 1365 CROssBAR knowledge graphs constructed with random term queries, including expected and observed frequencies, and Fisher's exact test significance values.

Biological comp. type	Term id	Term name	g_D*	G*	M_D*	Msum *	t*	Observed frequency (g_D/G)	Expected frequency (t^*M_D/M_{sum})	Fisher's exact test p-value	Is observed frequency significantly lower?
KEGG Pathway	hsa04151	PI3K-Akt signaling pathway	137	1365	354	21440	8.65	0.1004	0.1428	6.43E-06	Yes
KEGG Pathway	hsa04080	Neuroactive ligand-receptor interaction	168	1365	342	21440	8.65	0.1231	0.1380	1.33E-01	No
KEGG Pathway	hsa04010	MAPK signaling pathway	125	1365	296	21440	8.65	0.0916	0.1194	1.79E-03	Yes
KEGG Pathway	hsa04060	Cytokine-cytokine receptor interaction	57	1365	296	21440	8.65	0.0418	0.1194	1.74E-22	Yes
KEGG Pathway	hsa04144	Endocytosis	65	1365	250	21440	8.65	0.0476	0.1008	3.38E-12	Yes
KEGG Pathway	hsa04014	Ras signaling pathway	92	1365	236	21440	8.65	0.0674	0.0952	4.55E-04	Yes
KEGG Pathway	hsa04024	cAMP signaling pathway	82	1365	223	21440	8.65	0.0601	0.0900	9.65E-05	Yes
KEGG Pathway	hsa04015	Rap1 signaling pathway	102	1365	217	21440	8.65	0.0747	0.0875	1.12E-01	No
KEGG Pathway	hsa04810	Regulation of actin cytoskeleton	88	1365	213	21440	8.65	0.0645	0.0859	4.94E-03	Yes
KEGG Pathway	hsa04020	Calcium signaling pathway	94	1365	199	21440	8.65	0.0689	0.0803	1.35E-01	No
KEGG Pathway	hsa04510	Focal adhesion	113	1365	199	21440	8.65	0.0828	0.0803	7.20E-01	No
KEGG Pathway	hsa04621	NOD-like receptor signaling pathway	51	1365	181	21440	8.65	0.0374	0.0730	1.11E-07	Yes
KEGG Pathway	hsa04022	cGMP-PKG signaling pathway	64	1365	173	21440	8.65	0.0469	0.0698	8.81E-04	Yes
KEGG Pathway	hsa04218	Cellular senescence	60	1365	166	21440	8.65	0.0440	0.0670	5.67E-04	Yes
KEGG Pathway	hsa04630	JAK-STAT signaling pathway	47	1365	163	21440	8.65	0.0344	0.0658	1.07E-06	Yes
KEGG Pathway	hsa04310	Wnt signaling pathway	61	1365	159	21440	8.65	0.0447	0.0641	3.33E-03	Yes
KEGG Pathway	hsa04390	Hippo signaling pathway	85	1365	157	21440	8.65	0.0623	0.0633	9.54E-01	No
KEGG Pathway	hsa04150	mTOR signaling pathway	57	1365	155	21440	8.65	0.0418	0.0625	1.35E-03	Yes
KEGG Pathway	hsa04210	Apoptosis	70	1365	139	21440	8.65	0.0513	0.0561	5.03E-01	No
KEGG Pathway	hsa04068	FoxO signaling pathway	73	1365	130	21440	8.65	0.0535	0.0524	8.51E-01	No
Reactome Pathway	R-HSA-6798695	Neutrophil degranulation	41	1365	478	79623	9.26	0.0300	0.0556	1.17E-05	Yes
Reactome Pathway	R-HSA-381753	Olfactory Signaling Pathway	0	1365	432	79623	9.26	0.0000	0.0502	1.12E-30	Yes
Reactome Pathway	R-HSA-212436	Generic Transcription Pathway	14	1365	358	79623	9.26	0.0103	0.0416	1.37E-11	Yes
Reactome Pathway	R-HSA-983168	Antigen processing: Ubiquitination & Proteasome degradation	19	1365	309	79623	9.26	0.0139	0.0359	1.38E-06	Yes
Reactome Pathway	R-HSA-418594	G alpha (i) signalling events	49	1365	264	79623	9.26	0.0359	0.0307	2.68E-01	No
Reactome Pathway	R-HSA-8951664	Neddylation	8	1365	235	79623	9.26	0.0059	0.0273	1.56E-08	Yes

Reactome Pathway	R-HSA-5689880	Ub-specific processing proteases	6	1365	205	79623	9.26	0.0044	0.0238	2.39E-08	Yes
Reactome Pathway	R-HSA-198933	Immunoregulatory interactions between a Lymphoid and a non-Lymphoid cell	7	1365	201	79623	9.26	0.0051	0.0234	2.32E-07	Yes
Reactome Pathway	R-HSA-2467813	Separation of Sister Chromatids	6	1365	190	79623	9.26	0.0044	0.0221	1.79E-07	Yes
Reactome Pathway	R-HSA-72163	mRNA Splicing - Major Pathway	18	1365	180	79623	9.26	0.0132	0.0209	4.44E-02	Yes
Reactome Pathway	R-HSA-9010553	Regulation of expression of SLITs and ROBOs	3	1365	172	79623	9.26	0.0022	0.0200	1.02E-08	Yes
Reactome Pathway	R-HSA-6805567	Keratinization	31	1365	155	79623	9.26	0.0227	0.0180	2.17E-01	No
Reactome Pathway	R-HSA-202733	Cell surface interactions at the vascular wall	15	1365	151	79623	9.26	0.0110	0.0176	7.50E-02	No
Reactome Pathway	R-HSA-2871837	FCERI mediated NF-kB activation	0	1365	142	79623	9.26	0.0000	0.0165	3.48E-10	Yes
Reactome Pathway	R-HSA-194840	Rho GTPase cycle	3	1365	140	79623	9.26	0.0022	0.0163	1.05E-06	Yes
Reactome Pathway	R-HSA-114608	Platelet degranulation	5	1365	128	79623	9.26	0.0037	0.0149	9.82E-05	Yes
Reactome Pathway	R-HSA-381426	Regulation of Insulin-like Growth Factor (IGF) transport and uptake by Insulin-like Growth Factor Binding Proteins (IGFBPs)	12	1365	124	79623	9.26	0.0088	0.0144	8.47E-02	No
Reactome Pathway	R-HSA-6785807	Interleukin-4 and Interleukin-13 signaling	14	1365	111	79623	9.26	0.0103	0.0129	4.67E-01	No
Reactome Pathway	R-HSA-202424	Downstream TCR signaling	6	1365	104	79623	9.26	0.0044	0.0121	5.43E-03	Yes
Reactome Pathway	R-HSA-5620912	Anchoring of the basal body to the plasma membrane	12	1365	97	79623	9.26	0.0088	0.0113	5.15E-01	No
EFO Disease	Orphanet:791	Retinitis pigmentosa	17	1365	76	6798	7.48	0.0125	0.0837	1.37E-27	Yes
EFO Disease	Orphanet:90636	Autosomal recessive non-syndromic sensorineural deafness type DFNB	17	1365	63	6798	7.48	0.0125	0.0693	8.09E-21	Yes
EFO Disease	Orphanet:1934	Early infantile epileptic encephalopathy	26	1365	50	6798	7.48	0.0190	0.0550	9.22E-10	Yes
EFO Disease	Orphanet:154	Familial isolated dilated cardiomyopathy	10	1365	34	6798	7.48	0.0073	0.0374	4.66E-11	Yes
EFO Disease	Orphanet:244	Primary ciliary dyskinesia	6	1365	34	6798	7.48	0.0044	0.0374	6.95E-14	Yes
EFO Disease	Orphanet:88616	Autosomal recessive non-syndromic intellectual disability	12	1365	32	6798	7.48	0.0088	0.0352	7.97E-09	Yes
EFO Disease	EFO:0001360	type II diabetes mellitus	14	1365	27	6798	7.48	0.0103	0.0297	1.10E-05	Yes
EFO Disease	Orphanet:506	Leigh syndrome	3	1365	24	6798	7.48	0.0022	0.0264	3.24E-11	Yes
EFO Disease	Orphanet:2512	Autosomal recessive primary microcephaly	3	1365	21	6798	7.48	0.0022	0.0231	1.37E-09	Yes
EFO Disease	EFO:0000365	colorectal adenocarcinoma	8	1365	20	6798	7.48	0.0059	0.0220	1.24E-05	Yes
EFO Disease	Orphanet:84	Fanconi anemia	15	1365	18	6798	7.48	0.0110	0.0198	2.64E-02	Yes

EFO Disease	EFO:0001365	age-related macular degeneration	4	1365	17	6798	7.48	0.0029	0.0187	1.35E-06	Yes
EFO Disease	EFO:0000692	schizophrenia	8	1365	16	6798	7.48	0.0059	0.0176	7.00E-04	Yes
EFO Disease	Orphanet:666	Osteogenesis imperfecta	3	1365	16	6798	7.48	0.0022	0.0176	9.54E-07	Yes
EFO Disease	EFO:0000305	breast carcinoma	12	1365	15	6798	7.48	0.0088	0.0165	3.82E-02	Yes
EFO Disease	Orphanet:334	Familial atrial fibrillation	3	1365	14	6798	7.48	0.0022	0.0154	1.03E-05	Yes
EFO Disease	Orphanet:768	Familial long QT syndrome	9	1365	14	6798	7.48	0.0066	0.0154	1.05E-02	Yes
EFO Disease	Orphanet:1331	Familial prostate cancer	2	1365	13	6798	7.48	0.0015	0.0143	4.41E-06	Yes
EFO Disease	EFO:0002508	Parkinson's disease	12	1365	12	6798	7.48	0.0088	0.0132	2.27E-01	No
EFO Disease	EFO:0000685	rheumatoid arthritis	5	1365	11	6798	7.48	0.0037	0.0121	3.52E-03	Yes
KEGG Disease	H00266	Hereditary spastic paraplegia	20	1365	54	5985	6.92	0.0147	0.0624	1.73E-15	Yes
KEGG Disease	H01282	Spermatogenic failure	8	1365	39	5985	6.92	0.0059	0.0451	9.48E-16	Yes
KEGG Disease	H01202	Cataract	10	1365	37	5985	6.92	0.0073	0.0428	2.56E-13	Yes
KEGG Disease	H01439	Williams-Beuren syndrome	21	1365	28	5985	6.92	0.0154	0.0324	4.68E-04	Yes
KEGG Disease	H00093	Combined immunodeficiency	25	1365	25	5985	6.92	0.0183	0.0289	3.26E-02	Yes
KEGG Disease	H00058	Amyotrophic lateral sclerosis (ALS)	39	1365	24	5985	6.92	0.0286	0.0278	8.56E-01	No
KEGG Disease	H02106	Genetic obesity	24	1365	24	5985	6.92	0.0176	0.0278	3.70E-02	Yes
KEGG Disease	H00048	Hepatocellular carcinoma	8	1365	20	5985	6.92	0.0059	0.0231	7.23E-06	Yes
KEGG Disease	H00831	Primary dystonia	11	1365	20	5985	6.92	0.0081	0.0231	1.64E-04	Yes
KEGG Disease	H02410	Myelodysplastic/myeloproliferative neoplasms	6	1365	20	5985	6.92	0.0044	0.0231	3.75E-07	Yes
KEGG Disease	H01360	Allergic rhinitis	5	1365	19	5985	6.92	0.0037	0.0220	2.92E-07	Yes
KEGG Disease	H00821	Age-related macular degeneration	24	1365	17	5985	6.92	0.0176	0.0197	7.43E-01	No
KEGG Disease	H01492	Systemic sclerosis	12	1365	17	5985	6.92	0.0088	0.0197	5.72E-03	Yes
KEGG Disease	H00018	Gastric cancer	15	1365	16	5985	6.92	0.0110	0.0185	6.27E-02	No
KEGG Disease	H00032	Thyroid cancer	10	1365	16	5985	6.92	0.0073	0.0185	2.81E-03	Yes
KEGG Disease	H01667	Medulloblastoma	20	1365	16	5985	6.92	0.0147	0.0185	4.25E-01	No
KEGG Disease	H02411	Chronic myelomonocytic leukemia	10	1365	16	5985	6.92	0.0073	0.0185	2.81E-03	Yes
KEGG Disease	H00403	Disorders of nucleotide excision repair	11	1365	13	5985	6.92	0.0081	0.0150	5.17E-02	No
KEGG Disease	H00025	Penile cancer	14	1365	11	5985	6.92	0.0103	0.0127	5.85E-01	No
KEGG Disease	H00042	Glioma	13	1365	11	5985	6.92	0.0095	0.0127	4.10E-01	No
Phenotype (HPO)	HP:0000707	Abnormality of the nervous system	0	1365	3175	552814	9.22	0.0000	0.0530	1.37E-32	Yes

Phenotype (HPO)	HP:0000152	Abnormality of head or neck	0	1365	2597	552814	9.22	0.0000	0.0433	1.13E-26	Yes
Phenotype (HPO)	HP:0000924	Abnormality of the skeletal system	0	1365	2572	552814	9.22	0.0000	0.0429	2.73E-26	Yes
Phenotype (HPO)	HP:0003011	Abnormality of the musculature	0	1365	2295	552814	9.22	0.0000	0.0383	1.43E-23	Yes
Phenotype (HPO)	HP:0025031	Abnormality of the digestive system	0	1365	2287	552814	9.22	0.0000	0.0382	2.31E-23	Yes
Phenotype (HPO)	HP:0001626	Abnormality of the cardiovascular system	0	1365	2137	552814	9.22	0.0000	0.0357	8.03E-22	Yes
Phenotype (HPO)	HP:0000119	Abnormality of the genitourinary system	1	1365	2103	552814	9.22	0.0007	0.0351	5.84E-20	Yes
Phenotype (HPO)	HP:0001507	Growth abnormality	0	1365	2092	552814	9.22	0.0000	0.0349	1.60E-21	Yes
Phenotype (HPO)	HP:0001939	Abnormality of metabolism/homeostasis	7	1365	2031	552814	9.22	0.0051	0.0339	1.12E-12	Yes
Phenotype (HPO)	HP:0012443	Abnormality of brain morphology	0	1365	2027	552814	9.22	0.0000	0.0338	7.75E-21	Yes
Phenotype (HPO)	HP:0011804	Abnormal muscle physiology	0	1365	2004	552814	9.22	0.0000	0.0334	1.87E-20	Yes
Phenotype (HPO)	HP:0040064	Abnormality of limbs	0	1365	1761	552814	9.22	0.0000	0.0294	4.03E-18	Yes
Phenotype (HPO)	HP:0100022	Abnormality of movement	0	1365	1707	552814	9.22	0.0000	0.0285	1.28E-17	Yes
Phenotype (HPO)	HP:0000951	Abnormality of the skin	1	1365	1705	552814	9.22	0.0007	0.0284	5.99E-16	Yes
Phenotype (HPO)	HP:0001250	Seizure	1	1365	1514	552814	9.22	0.0007	0.0253	6.17E-14	Yes
Phenotype (HPO)	HP:0000708	Behavioral abnormality	1	1365	1389	552814	9.22	0.0007	0.0232	8.17E-13	Yes
Phenotype (HPO)	HP:0004325	Decreased body weight	0	1365	1117	552814	9.22	0.0000	0.0186	1.41E-11	Yes
Phenotype (HPO)	HP:0004322	Short stature	0	1365	1098	552814	9.22	0.0000	0.0183	2.05E-11	Yes
Phenotype (HPO)	HP:0031826	Abnormal reflex	0	1365	1020	552814	9.22	0.0000	0.0170	1.43E-10	Yes
Phenotype (HPO)	HP:0002664	Neoplasm	8	1365	805	552814	9.22	0.0059	0.0134	1.26E-02	Yes
Drug	DB12010	Fostamatinib	60	1365	304	19028	7.66	0.0440	0.1223	3.04E-22	Yes
Drug	DB00157	NADH	37	1365	144	19028	7.66	0.0271	0.0580	2.47E-07	Yes
Drug	DB01593	Zinc	29	1365	124	19028	7.66	0.0212	0.0499	1.80E-07	Yes
Drug	DB11638	Artenimol	32	1365	107	19028	7.66	0.0234	0.0431	2.51E-04	Yes
Drug	DB02379	Beta-D-Glucose	2	1365	78	19028	7.66	0.0015	0.0314	8.02E-16	Yes
Drug	DB00142	Glutamic acid	10	1365	69	19028	7.66	0.0073	0.0278	2.67E-07	Yes
Drug	DB00334	Olanzapine	0	1365	48	19028	7.66	0.0000	0.0193	1.13E-11	Yes
Drug	DB00543	Amoxapine	0	1365	44	19028	7.66	0.0000	0.0177	1.34E-10	Yes
Drug	DB12695	Phenethyl Isothiocyanate	28	1365	44	19028	7.66	0.0205	0.0177	4.58E-01	No
Drug	DB08231	Myristic acid	2	1365	43	19028	7.66	0.0015	0.0173	4.87E-08	Yes
Drug	DB00321	Amitriptyline	0	1365	35	19028	7.66	0.0000	0.0141	1.74E-08	Yes
Drug	DB00555	Lamotrigine	3	1365	35	19028	7.66	0.0022	0.0141	2.13E-05	Yes
Drug	DB01708	Prasterone	0	1365	35	19028	7.66	0.0000	0.0141	1.74E-08	Yes
Drug	DB11093	Calcium citrate	6	1365	33	19028	7.66	0.0044	0.0133	2.39E-03	Yes

Drug	DB00201	Caffeine	13	1365	32	19028	7.66	0.0095	0.0129	3.78E-01	No
Drug	DB00408	Loxapine	1	1365	32	19028	7.66	0.0007	0.0129	1.11E-06	Yes
Drug	DB00909	Zonisamide	5	1365	31	19028	7.66	0.0037	0.0125	1.68E-03	Yes
Drug	DB00312	Pentobarbital	0	1365	28	19028	7.66	0.0000	0.0113	6.09E-07	Yes
Drug	DB04216	Quercetin	9	1365	28	19028	7.66	0.0066	0.0113	1.36E-01	No
Drug	DB00363	Clozapine	0	1365	27	19028	7.66	0.0000	0.0109	9.00E-07	Yes
Gene/ Protein	Q6A162	KRT40	1	1365	275	62455	8.42	0.0007	0.0371	5.84E-21	Yes
Gene/ Protein	Q08379	GOLGA2	2	1365	248	62455	8.42	0.0015	0.0334	2.21E-17	Yes
Gene/ Protein	Q7Z3S9	NOTCH2NLA	8	1365	208	62455	8.42	0.0059	0.0280	7.47E-09	Yes
Gene/ Protein	P60409	KRTAP10-7	4	1365	197	62455	8.42	0.0029	0.0266	2.46E-11	Yes
Gene/ Protein	P14373	TRIM27	1	1365	172	62455	8.42	0.0007	0.0232	1.13E-12	Yes
Gene/ Protein	P50222	MEOX2	8	1365	167	62455	8.42	0.0059	0.0225	2.47E-06	Yes
Gene/ Protein	Q15323	KRT31	4	1365	161	62455	8.42	0.0029	0.0217	1.11E-08	Yes
Gene/ Protein	P04637	TP53	3	1365	155	62455	8.42	0.0022	0.0209	3.18E-09	Yes
Gene/ Protein	P60410	KRTAP10-8	3	1365	155	62455	8.42	0.0022	0.0209	3.18E-09	Yes
Gene/ Protein	P60411	KRTAP10-9	3	1365	153	62455	8.42	0.0022	0.0206	4.58E-09	Yes
Gene/ Protein	Q04864	REL	14	1365	152	62455	8.42	0.0103	0.0205	6.21E-03	Yes
Gene/ Protein	Q99750	MDFI	4	1365	149	62455	8.42	0.0029	0.0201	6.20E-08	Yes
Gene/ Protein	Q9BRK4	LZTS2	1	1365	146	62455	8.42	0.0007	0.0197	1.08E-10	Yes
Gene/ Protein	Q5JR59	MTUS2	7	1365	145	62455	8.42	0.0051	0.0195	1.41E-05	Yes
Gene/ Protein	O00560	SDCBP	9	1365	140	62455	8.42	0.0066	0.0189	2.42E-04	Yes
Gene/ Protein	P00533	EGFR	6	1365	130	62455	8.42	0.0044	0.0175	3.19E-05	Yes
Gene/ Protein	P15884	TCF4	6	1365	130	62455	8.42	0.0044	0.0175	3.19E-05	Yes
Gene/ Protein	Q12933	TRAF2	1	1365	128	62455	8.42	0.0007	0.0173	3.41E-09	Yes
Gene/ Protein	P62993	GRB2	7	1365	126	62455	8.42	0.0051	0.0170	1.63E-04	Yes
Gene/ Protein	Q8TBB1	LNX1	7	1365	122	62455	8.42	0.0051	0.0164	3.00E-04	Yes

* g_D: number of KGs where term D is presented, *G*: total number of KGs, *t*: number of terms/nodes in each KG from the same biological component as term *D*, M_D: the total number of genes/proteins that are associated with term *D*, M_{sum}: the total number of associations between all genes/proteins and all terms in the same biological component as term *D*.

Table S8. The list of query terms for 1365 CROssBAR knowledge graphs.

**Gene/protein queries (UniProt protein accessions)**					
P41279	Q08881	Q13370	P50616	P61081	Q7LBR1
Q9Y2M5	Q96QH2	P17987	Q86Y82	Q30201	Q96SB3
Q5JZY3	A1L3X4	P10636	P40337	Q5T5X7	Q9UBQ0
Q92841	Q8NAV1	Q15653	Q9H3H3	P28749	Q8NFJ9
Q9NPG2	P15336	Q5T5A8	Q14669	Q6IQ23	Q9UBT7
Q96Q35	Q5T4F4	Q9P0T4	Q5W5X9	Q2TAY7	Q12906
Q9UQ90	O14745	Q13064	P04233	Q9HD67	O95447
P04233	P43034	Q8TEW0	O60885	Q9UJY4	Q14978
Q9HCJ0	P48729	Q969Q1	P11831	P42768	Q96A56
Q9HCU8	Q86U70	Q3T906	O75022	P07195	Q6W2J9
P40617	Q13127	O60563	Q13835	P55199	P46098
Q96AH0	P23467	Q8TBZ8	O76071	Q9NZL4	P09228
Q70UQ0	Q9P0U4	Q14919	Q9NX70	Q53GG5	Q14164
O60566	Q86Y13	Q9BYE7	Q8TBC4	P09913	Q5T124
Q969T4	P49760	Q9H0F6	Q9BU76	P67775	B2RUY7
Q9UJ04	Q9Y2X0	Q14152	Q15025	O96028	Q53GI3
Q7L4P6	P36896	P16473	Q6IBW4	Q09FC8	Q9UNI6
O43704	P60981	O15116	Q01484	P43351	Q92688
Q9NQX5	B2RUY7	O00303	Q9UGP5	Q96GE4	O14936
O15389	Q8IYX3	Q8NDC4	O00512	Q8NA82	Q8WYQ5
P48061	O75581	Q8N5A5	Q9NRM7	Q96GS4	O43561
P46779	P09629	Q9UQM7	Q5TD97	P09622	O43157
Q06455	Q9P0L0	P30307	O15131	Q9Y223	Q04941
O95983	Q15459	Q13064	Q15528	Q13867	P29373
Q92636	O15524	Q13496	Q14562	Q9UBU8	Q504Q3
O14613	P26678	Q66K89	Q9BYM8	Q7Z7H3	P61962
Q9Y2R2	Q14929	Q16629	P13688	P38432	O75031
P29144	O14627	Q71DI3	Q9UBU8	Q08AF8	Q96EQ0
Q01201	P50461	P51668	O14653	O15031	O95352
Q8N9W6	Q13363	Q49A88	Q71U36	P84090	O75564
O60684	Q6XZF7	Q9BYQ2	P28749	Q13541	
Q96BY2	O00746	Q9HCU9	Q8TDY4	Q15399	
Q9H0I2	Q92556	P35240	Q13049	P08670	
O15085	Q969G2	Q15262	Q8IYX3	P55735	

**Reactome pathway queries (pathway names)**	
Neurofascin interactions	Activation of DNA fragmentation factor
tRNA processing in the mitochondrion	DEx/H-box helicases activate type I IFN and inflammatory cytokines production
Translocation of ZAP-70 to Immunological synapse	IRAK1 recruits IKK complex upon TLR7/8 or 9 stimulation
Negative regulation of the PI3K/AKT network	Endosomal/Vacuolar pathway

Pyrimidine catabolism	Synthesis of IP3 and IP4 in the cytosol
Trafficking of AMPA receptors	VLDLR internalisation and degradation
IRAK1 recruits IKK complex	Transport of Ribonucleoproteins into the Host Nucleus
AXIN missense mutants destabilize the destruction complex	ERCC6 (CSB) and EHMT2 (G9a) positively regulate rRNA expression
Removal of aminoterminal propeptides from gamma-carboxylated proteins	Chylomicron assembly
Recognition and association of DNA glycosylase with site containing an affected pyrimidine	SeMet incorporation into proteins
CTLA4 inhibitory signaling	Other interleukin signaling
Presynaptic phase of homologous DNA pairing and strand exchange	Tristetraprolin (TTP, ZFP36) binds and destabilizes mRNA
Phosphate bond hydrolysis by NUDT proteins	Highly calcium permeable postsynaptic nicotinic acetylcholine receptors
PI-3K cascade:FGFR3	Regulation of signaling by NODAL
Netrin mediated repulsion signals	DNA methylation
Glyoxylate metabolism and glycine degradation	Surfactant metabolism
Pausing and recovery of HIV elongation	Hh mutants that don't undergo autocatalytic processing are degraded by ERAD
Beta oxidation of decanoyl-CoA to octanoyl-CoA-CoA	PLC beta mediated events
Estrogen biosynthesis	Attenuation phase
NTRK2 activates RAC1	Cristae formation
Fibronectin matrix formation	Bicarbonate transporters
Formation of HIV elongation complex in the absence of HIV Tat	Formation of TC-NER Pre-Incision Complex
Fatty acyl-CoA biosynthesis	SUMOylation of SUMOylation proteins
MECP2 regulates transcription factors	SUMO is conjugated to E1 (UBA2:SAE1)
TRAF6 mediated IRF7 activation in TLR7/8 or 9 signaling	FasL/ CD95L signaling
Heme degradation	Cross-presentation of soluble exogenous antigens (endosomes)
Carboxyterminal post-translational modifications of tubulin	Mismatch repair (MMR) directed by MSH2:MSH3 (MutSbeta)
TFAP2A acts as a transcriptional repressor during retinoic acid induced cell differentiation	Methionine salvage pathway
Electric Transmission Across Gap Junctions	Glutathione conjugation
Eicosanoids	Clathrin derived vesicle budding
Signal regulatory protein family interactions	DAG and IP3 signaling
Mitochondrial Fatty Acid Beta-Oxidation	Regulation of necroptotic cell death
Opsins	Meiotic recombination
Intraflagellar transport	Signaling by cytosolic FGFR1 fusion mutants
Synthesis of glycosylphosphatidylinositol (GPI)	mitochondrial fatty acid beta-oxidation of unsaturated fatty acids
Electric Transmission Across Gap Junctions	Negative regulation of activity of TFAP2 (AP-2) family transcription factors
Advanced glycosylation endproduct receptor signaling	Norepinephrine Neurotransmitter Release Cycle
N-Glycan antennae elongation	Stabilization of p53
Truncations of AMER1 destabilize the destruction complex	Glutathione conjugation
VxPx cargo-targeting to cilium	Serotonin receptors
Ficolins bind to repetitive carbohydrate structures on the target cell surface	PTK6 Expression
PTK6 Expression	Aryl hydrocarbon receptor signalling
RAF-independent MAPK1/3 activation	NCAM signaling for neurite out-growth

RUNX3 regulates BCL2L11 (BIM) transcription	RUNX1 and FOXP3 control the development of regulatory T lymphocytes (Tregs)
p130Cas linkage to MAPK signaling for integrins	Phase 4 - resting membrane potential
PI3K events in ERBB2 signaling	TFAP2A acts as a transcriptional repressor during retinoic acid induced cell differentiation
Synthesis of IP3 and IP4 in the cytosol	PI3K Cascade
Activation of E2F1 target genes at G1/S	Interleukin-3, Interleukin-5 and GM-CSF signaling
Nitric oxide stimulates guanylate cyclase	SMAC(DIABLO)-mediated dissociation of IAP:caspase complexes
CHL1 interactions	Synthesis of PA
**KEGG pathway queries (pathway names)**	
Various types of N-glycan biosynthesis	N-Glycan biosynthesis
Lipoic acid metabolism	Starch and sucrose metabolism
Glycosaminoglycan biosynthesis	Glutathione metabolism
Porphyrin and chlorophyll metabolism	Glycosphingolipid biosynthesis
Carbohydrate digestion and absorption	Circadian rhythm
Ascorbate and aldarate metabolism	Pentose and glucuronate interconversions
Glycosphingolipid biosynthesis	Aminoacyl-tRNA biosynthesis
Thiamine metabolism	Butanoate metabolism
Thiamine metabolism	Pyruvate metabolism
Vasopressin-regulated water reabsorption	N-Glycan biosynthesis
Ascorbate and aldarate metabolism	Arginine and proline metabolism
Synthesis and degradation of ketone bodies	N-Glycan biosynthesis
Sphingolipid metabolism	SNARE interactions in vesicular transport
Non-homologous end-joining	Sphingolipid metabolism
Riboflavin metabolism	Galactose metabolism
Fatty acid degradation	Riboflavin metabolism
Hedgehog signaling pathway	Tyrosine metabolism
beta-Alanine metabolism	Cholesterol metabolism
Thiamine metabolism	Biosynthesis of unsaturated fatty acids
Pentose phosphate pathway	Synthesis and degradation of ketone bodies
SNARE interactions in vesicular transport	DNA replication
Circadian rhythm	Notch signaling pathway
Sphingolipid metabolism	Other types of O-glycan biosynthesis
Carbohydrate digestion and absorption	Hedgehog signaling pathway
Synthesis and degradation of ketone bodies	One carbon pool by folate
Glycosaminoglycan biosynthesis	Citrate cycle (TCA cycle)
Homologous recombination	Vitamin digestion and absorption
Taurine and hypotaurine metabolism	Arginine biosynthesis
Cysteine and methionine metabolism	Biosynthesis of unsaturated fatty acids
Citrate cycle (TCA cycle)	Mannose type O-glycan biosynthesis
Steroid biosynthesis	Synthesis and degradation of ketone bodies
Starch and sucrose metabolism	Proximal tubule bicarbonate reclamation
Base excision repair	Synthesis and degradation of ketone bodies

Linoleic acid metabolism	Pentose phosphate pathway
Sulfur metabolism	Other types of O-glycan biosynthesis
Selenocompound metabolism	N-Glycan biosynthesis
N-Glycan biosynthesis	Steroid biosynthesis
Various types of N-glycan biosynthesis	Nicotinate and nicotinamide metabolism
Propanoate metabolism	Vitamin digestion and absorption
Taurine and hypotaurine metabolism	Renin-angiotensin system
Apoptosis	Butanoate metabolism
Glycine, serine and threonine metabolism	Steroid biosynthesis
Glycosylphosphatidylinositol (GPI)-anchor biosynthesis	Circadian rhythm
beta-Alanine metabolism	Mucin type O-glycan biosynthesis
Riboflavin metabolism	Glyoxylate and dicarboxylate metabolism
Vitamin B6 metabolism	Caffeine metabolism
Glycosphingolipid biosynthesis	Fructose and mannose metabolism
Glycosaminoglycan biosynthesis	Cholesterol metabolism
Pantothenate and CoA biosynthesis	Glycine, serine and threonine metabolism
Riboflavin metabolism	Histidine metabolism
**EFO disease queries (disease names)**	
Infantile bilateral striatal necrosis	Isolated focal cortical dysplasia type IIa
sporadic amyotrophic lateral sclerosis	HIV-1 infection
Cowden syndrome	Hyper-IgM syndrome with susceptibility to opportunistic infections
Alzheimer's disease	Generalized epilepsy with febrile seizures-plus
Hypokalemic periodic paralysis	Split hand-split foot malformation
Rh deficiency syndrome	Renal tubular dysgenesis of genetic origin
familial hypercholesterolemia	Sulfite oxidase deficiency due to molybdenum cofactor deficiency
Primary hyperoxaluria	Adams-Oliver syndrome
Short rib-polydactyly syndrome	restless legs syndrome
Autosomal recessive cutis laxa type 2	Stargardt disease
Generalized pustular psoriasis	Autosomal dominant progressive external ophthalmoplegia
embryonal rhabdomyosarcoma	focal segmental glomerulosclerosis
Ehlers-Danlos syndrome, classic type	Stargardt disease
Familial hemophagocytic lymphohistiocytosis	Fetal akinesia deformation sequence
Matthew-Wood syndrome	Zimmermann-Laband syndrome
Activated PIK3-delta syndrome	Lamellar ichthyosis
Familial infantile bilateral striatal necrosis	Autosomal recessive distal renal tubular acidosis
Localized junctional epidermolysis bullosa, non-Herlitz type	Distal hereditary motor neuropathy type 2
Branchio-otic syndrome	Tangier disease
Primary systemic amyloidosis	Persistent Müllerian duct syndrome
Syndactyly type 2	Paroxysmal nocturnal hemoglobinuria
Distal myopathy, Nonaka type	Juvenile or adult CACH syndrome
MELAS	Generalized junctional epidermolysis bullosa, non-Herlitz type

Facioscapulohumeral dystrophy	Familial hypodysfibrinogenemia
Krabbe disease	Ondine syndrome
Agammaglobulinemia	chronic myeloproliferative disorder
Familial focal epilepsy with variable foci	red-green color blindness
obsolete_Alagille syndrome	Cardiofaciocutaneous syndrome
congenital disorder of glycosylation type II	Junctional epidermolysis bullosa, non-Herlitz type
Immunodeficiency due to a complement cascade protein anomaly	Juvenile polyposis of infancy
Annular epidermolytic ichthyosis	Hereditary chronic pancreatitis
Dowling-Degos disease	Hyper-IgM syndrome with susceptibility to opportunistic infections
Lethal congenital contracture syndrome type 3	Mitochondrial myopathy and sideroblastic anemia
Partial pancreatic agenesis	Coffin-Siris syndrome
Pontocerebellar hypoplasia type 1	Combined pituitary hormone deficiencies, genetic forms
congenital heart malformation	Phosphoenolpyruvate carboxykinase deficiency
Oligodontia	Myopia
Waardenburg syndrome type 2	Perrault syndrome
Autosomal recessive spastic ataxia with leukoencephalopathy	Sotos syndrome
Adenosine monophosphate deaminase deficiency	Uncombable hair syndrome
Pseudoxanthoma elasticum	Kenny-Caffey syndrome
Congenital muscular dystrophy, Ullrich type	X-linked non-syndromic sensorineural deafness type DFN
Saldino-Mainzer syndrome	Piebaldism
Familial hypospadias	Triphalangeal thumb - polysyndactyly syndrome
Schizencephaly	Barrett's esophagus
Prader-Willi syndrome	osteoporosis
medulloblastoma	De Barsy syndrome
Posterior polar cataract	Metaphyseal anadysplasia
Rare isolated myopia	Jeune syndrome
Lacrimoauriculodentodigital syndrome	Familial episodic pain syndrome
**KEGG disease queries (disease names)**	
Pitt-Hopkins syndrome	Autosomal dominant tubulointerstitial kidney disease
Spastic quadriplegic cerebral palsy	Chromosome 15q13.3 microdeletion syndrome
3MC syndrome	Bruck syndrome
Proximal symphalangism	Fatal infantile cardioencephalomyopathy
SEMD with joint laxity type	Thalassemia
Familial epilepsy temporal lobe (ETL)	Vogt-Koyanagi-Harada syndrome
Hyperkalemic distal renal tubular acidosis (RTA type 4)	Medullary thyroid cancer
Anemia due to disorders of nucleotide metabolism	Pachyonychia congenita
Primary hyperparathyroidism	Postaxial polydactyly
Multi-minicore disease	Coronary artery disease
Anemia due to disorders of glutathione metabolism	Cerebral creatine deficiency syndrome
D-2-hydroxyglutaric aciduria	Infantile hypotonia with psychomotor retardation and characteristic facies
Immunodeficiency associated with DNA repair defects	Non-epidermolytic palmoplantar keratoderma

Oculocutaneous albinism	Xeroderma pigmentosum
Dandy-Walker syndrome	Van Maldergem syndrome
Other phagocyte defects	Familial Mediterranean fever
Rett syndrome	Familial partial lipodystrophy
Anemia due to disorders of nucleotide metabolism	Wiskott-Aldrich syndrome
Celiac disease	Mismatch repair deficiency
Warburg micro syndrome	Pattern dystrophies of the retinal pigment epithelium
AMP deaminase deficiency	Vesicoureteral reflux
Bare lymphocyte syndrome type2	Bilateral sudden sensorineural hearing loss
Familial focal epilepsy with variable foci	Pituitary adenomas
Obsessive-compulsive disorder	Lymphangioleiomyomatosis
Hyperbilirubinemia	Radioulnar synostosis with amegakaryocytic thrombocytopenia
Chronic granulomatous disease	Down syndrome
Central areolar choroidal dystrophy	Permanent neonatal diabetes mellitus
Congenital adrenal hyperplasia	Myelofibrosis
Lewy body dementia (LBD)	Pyruvate dehydrogenase complex deficiency
Nonsyndromic congenital nail disorder	Ectopia lentis
Polycystic liver disease	Blue cone monochromacy
Hereditary hypotrichosis simplex	Heparan sulfate proteoglycan gene defects
Zimmermann-Laband syndrome	Dyslexia
Oral cancer	Porencephaly
Familial hemophagocytic lymphohistiocytosis	Hypopituitarism
Malignant paraganglioma	Kearns-Sayre syndrome
Spondyloepiphyseal dysplasia tarda	Hereditary stomatocytosis
Ellis-van Creveld syndrome	Dent disease
Primary aldosteronism	Desbuquois syndrome
Familial Mediterranean fever	Tuberous sclerosis complex
Congenital mirror movements	Scapuloperoneal myopathy
Budd-Chiari syndrome	Hyperlipidemia
Mosaic variegated aneuploidy syndrome	Microphthalmia with linear skin defects syndrome
Tricho-hepato-enteric syndrome	Vogt-Koyanagi-Harada syndrome
Multiple sclerosis	Xanthinuria
Pseudoxanthoma elasticum	Cerebral creatine deficiency syndrome
Frontonasal dysplasia	Familial adult myoclonic epilepsy
Emery-Dreifuss muscular dystrophy	Brachyolmia
Miyoshi myopathy	Nocturnal frontal lobe epilepsy
Tyrosinemia	Infantile liver failure

Phenotype queries (HPO term name)

Abnormal thalamic size	Ulnar deviation of the 2nd finger	Broad alveolar ridges
Recurrent cutaneous abscess formation	Decreased serum estradiol	Episodic abdominal pain
Prolinuria	Abnormal vitreous humor morphology	Bowel incontinence

Laryngeal hypoplasia	Upper eyelid coloboma	Myoclonic spasms
Bull's eye maculopathy	Sudden death	Conical incisor
Fat malabsorption	Periauricular skin pits	Anterior pituitary dysgenesis
Asymmetric growth	Craniofacial asymmetry	Elevated alpha-fetoprotein
Hyperphosphaturia	Arrhinencephaly	Vertebral clefting
Temporal pattern	Severe infection	Metrorrhagia
Abnormal foveal morphology	Decreased serum estradiol	Brachyturricephaly
Type 1 muscle fiber atrophy	Pierre-Robin sequence	Abnormality of the 1st metacarpal
Pustule	Increased circulating renin level	Periodic hypokalemic paresis
Incomitant strabismus	Abdominal wall muscle weakness	Enchondroma
Decreased circulating luteinizing hormone level	Persistence of hemoglobin F	Pseudoepiphyses of hand bones
Hyperaldosteronism	Reduced systolic function	Urinary bladder sphincter dysfunction
Large earlobe	Pseudoepiphyses of hand bones	Impaired T cell function
Decreased patellar reflex	Tracheomalacia	Frontalis muscle weakness
Toxemia of pregnancy	Decreased serum leptin	Episodic respiratory distress
Exercise-induced myalgia	11 pairs of ribs	Centrocecal scotoma
Joint subluxation	Metamorphopsia	Severe intrauterine growth retardation
Stooped posture	Abnormal serum bile acid concentration	Abnormal urine hormone level
Expressive language delay	Impaired proprioception	Visual acuity test abnormality
Deviation of the 5th toe	Reduced factor XIII activity	Barrett esophagus
Narrow vertebral interpedicular distance	Aplasia/Hypoplasia involving the skeletal musculature	Narrow sacroiliac notch
Palmar hyperhidrosis	Enlarged pituitary gland	Quadriceps muscle weakness
Nasal regurgitation	Epicanthus inversus	Abnormal natural killer cell morphology
Abnormal pupillary light reflex	Generalized hypopigmentation	Fetal distress
Premature graying of hair	Hypokalemic alkalosis	Abnormal renal collecting system morphology
Acute lymphoblastic leukemia	Low serum calcitriol	Low levels of vitamin A
Thin nail	Macular edema	Peritonitis
Humeroradial synostosis	Aplasia/Hypoplasia of the cochlea	Choroidal neovascularization
EEG with spike-wave complexes (2.5-3.5 Hz)	Flattened femoral epiphysis	Persistent bleeding after trauma
Abnormality of the distal phalanx of the 5th finger	Female infertility	Ectopia pupillae
Increased circulating cortisol level	Caudate atrophy	Abnormality of the intrahepatic bile duct
Shallow acetabular fossae	Nephronophthisis	Pendular nystagmus
Hypoplastic vertebral bodies	Progressive gait ataxia	Thromboembolism
Thenar muscle atrophy	Septate vagina	Torticollis
Chronic hepatic failure	Abnormal circulating methionine concentration	Intestinal pseudo-obstruction
Heat intolerance	Patchy changes of bone mineral density	Bilateral microphthalmos
Vertical supranuclear gaze palsy	Noncompaction cardiomyopathy	Reduced factor XII activity
Aplasia/Hypoplasia of the scapulae	Abnormal cell morphology	Triangular mouth
Hypoplastic heart	Hyperintensity of cerebral white matter on MRI	Abnormal response to endocrine stimulation test
Prominent supraorbital ridges	Abnormality of the ischium	Abnormality of DNA repair

Decreased thalamic volume	Reduced factor XI activity	Low plasma citrulline
Recurrent infection of the gastrointestinal tract	Abnormal size of pituitary gland	Square face
Thyrotoxicosis with diffuse goiter	Anteverted ears	Sea-blue histiocytosis
Acral ulceration	Abnormal eyelid physiology	Absent toenail
Anterior open bite	Decreased patellar reflex	Calcification of falx cerebri
Rectal polyposis	Prolonged bleeding time	Small basal ganglia
Tracheomalacia	Macrocephaly at birth	Accessory oral frenulum
Abnormality of the lumbar spine	Ovotestis	Recurrent upper and lower respiratory tract infections
Leydig cell neoplasia	Abnormal urine potassium concentration	Retinal nonattachment
Subglottic stenosis	Aplasia/Hypoplasia of the phalanges of the toes	Neoplasm of the gallbladder
Abnormal sweat homeostasis	Abnormality of the peritoneum	Oval face
Pulmonary opacity	Abnormal serum interferon-gamma level	Abnormal mean corpuscular hemoglobin concentration
Sparse axillary hair	Hypercapnia	Limb myoclonus
Conjunctival hamartoma	Multiple renal cysts	Erythema nodosum
Abnormal renal artery morphology	Asymmetry of the thorax	Erythroderma
Myofibrillar myopathy	Low levels of vitamin E	Drowsiness
Aplasia/Hypoplasia of the middle phalanx of the 2nd finger	Calcification of the aorta	Hypoproteinemia
Angioid streaks of the fundus	Chylothorax	Loss of subcutaneous adipose tissue in limbs
Ulnar bowing	Antiphospholipid antibody positivity	Fetal distress
Pseudoepiphyses of hand bones	Lower limb asymmetry	Calcium oxalate nephrolithiasis
Genu recurvatum	Heparan sulfate excretion in urine	Increased skull ossification
Writer's cramp	Biliary atresia	Focal impaired awareness seizure
Overfolded helix	Thoracolumbar scoliosis	Hypoplastic ischia
Aplasia of the musculature	Hemoglobinuria	

Drug queries (DrugBank drug id)

DB00193	DB00054	DB04841	DB02526	DB01686	DB00848
DB00798	DB03434	DB00275	DB03876	DB13995	DB02402
DB00521	DB00677	DB13346	DB05552	DB13179	DB05169
DB00592	DB06401	DB09092	DB04573	DB01083	DB02375
DB00673	DB07150	DB07664	DB02142	DB00431	DB04576
DB00039	DB04132	DB01561	DB01744	DB03900	DB00521
DB00736	DB08867	DB06594	DB00066	DB03403	DB01364
DB00587	DB04237	DB00004	DB07841	DB03541	DB05472
DB08217	DB02329	DB08018	DB07857	DB03900	DB04846
DB07218	DB04574	DB01011	DB00399	DB01766	DB03691
DB01118	DB05944	DB00541	DB00985	DB07374	DB03309
DB02076	DB00657	DB00146	DB01050	DB13994	DB00374
DB00093	DB03487	DB01033	DB03945	DB00433	DB01834
DB07091	DB02557	DB06021	DB04083	DB08888	DB06831
DB00610	DB00591	DB05882	DB00636	DB08855	DB01353

DB02559	DB01268	DB00641	DB01558	DB08073	DB00275
DB00129	DB09244	DB00820	DB06714	DB13923	DB06719
DB08403	DB00613	DB00431	DB07652	DB00383	DB08402
DB00322	DB08811	DB00368	DB09033	DB00122	DB03136
DB03449	DB01011	DB00243	DB02153	DB06831	DB04334
DB06070	DB01234	DB06802	DB01062	DB00877	DB02515
DB05459	DB05777	DB00322	DB00048	DB04557	DB12228
DB00938	DB09166	DB13151	DB07218	DB01103	DB12698
DB02559	DB11691	DB00823	DB02824	DB00098	DB07662
DB01043	DB04185	DB06202	DB00044	DB00210	DB07137
DB04387	DB12023	DB08896	DB03128	DB00414	DB02281
DB03088	DB00610	DB08059	DB00276	DB00565	DB00545
DB00692	DB00530	DB00590	DB02552	DB00912	DB07530
DB01592	DB00656	DB07131	DB00472	DB00031	DB00294
DB00125	DB05676	DB02998	DB09052	DB02701	DB13951
DB07812	DB13872	DB00181	DB00966	DB00042	
DB04016	DB08222	DB00768	DB00541	DB07712	
DB03978	DB09099	DB01203	DB01643	DB00934	
DB00511	DB07562	DB00254	DB08568	DB00160	
**Compound queries (ChEMBL compound id)**					
CHEMBL1540570	CHEMBL1333386	CHEMBL1241950	CHEMBL566340	CHEMBL113621	CHEMBL1347030
CHEMBL204426	CHEMBL164660	CHEMBL1585390	CHEMBL432038	CHEMBL65374	CHEMBL181633
CHEMBL1784637	CHEMBL319557	CHEMBL1356395	CHEMBL244990	CHEMBL7204	CHEMBL140872
CHEMBL4213352	CHEMBL1544947	CHEMBL1969506	CHEMBL91636	CHEMBL2377590	CHEMBL121405
CHEMBL1760035	CHEMBL1437753	CHEMBL1561738	CHEMBL1887153	CHEMBL1402587	CHEMBL1406702
CHEMBL1583049	CHEMBL2333956	CHEMBL1565328	CHEMBL93577	CHEMBL76897	CHEMBL1377687
CHEMBL1550048	CHEMBL2380394	CHEMBL3193196	CHEMBL1650951	CHEMBL1519472	CHEMBL266292
CHEMBL2403369	CHEMBL332898	CHEMBL1459746	CHEMBL2004118	CHEMBL4209440	CHEMBL4095596
CHEMBL28079	CHEMBL673	CHEMBL2414354	CHEMBL1405834	CHEMBL7724	CHEMBL476513
CHEMBL1427061	CHEMBL2312168	CHEMBL3335300	CHEMBL4168669	CHEMBL2001712	CHEMBL1558070
CHEMBL1200348	CHEMBL1347071	CHEMBL4293085	CHEMBL1425715	CHEMBL1998953	CHEMBL1457902
CHEMBL3986068	CHEMBL24057	CHEMBL3647677	CHEMBL2396719	CHEMBL2315244	CHEMBL199405
CHEMBL242644	CHEMBL2112774	CHEMBL17645	CHEMBL4069601	CHEMBL254857	CHEMBL59687
CHEMBL3670703	CHEMBL3401990	CHEMBL3212972	CHEMBL1417159	CHEMBL1611170	CHEMBL403402
CHEMBL45	CHEMBL1451772	CHEMBL1525599	CHEMBL448784	CHEMBL1313485	CHEMBL3622193
CHEMBL453066	CHEMBL1411285	CHEMBL12375	CHEMBL1343689	CHEMBL4080797	CHEMBL1454931
CHEMBL302998	CHEMBL31354	CHEMBL1987579	CHEMBL4087906	CHEMBL1452003	CHEMBL294878
CHEMBL1112	CHEMBL1422230	CHEMBL1558822	CHEMBL1966722	CHEMBL1550184	CHEMBL498373
CHEMBL352039	CHEMBL3586404	CHEMBL1329712	CHEMBL1086997	CHEMBL1589224	CHEMBL491771
CHEMBL576409	CHEMBL310843	CHEMBL2324753	CHEMBL2003304	CHEMBL381207	CHEMBL1337226
CHEMBL164	CHEMBL67716	CHEMBL1967094	CHEMBL303313	CHEMBL1311826	CHEMBL3128043
CHEMBL1558893	CHEMBL1583414	CHEMBL97333	CHEMBL398765	CHEMBL4288256	CHEMBL460291

CHEMBL1999630	CHEMBL553833	CHEMBL1522275	CHEMBL1602926	CHEMBL4087132	CHEMBL1309430
CHEMBL1241770	CHEMBL322970	CHEMBL1813315	CHEMBL236902	CHEMBL284377	CHEMBL580727
CHEMBL1076	CHEMBL25230	CHEMBL214332	CHEMBL4090770	CHEMBL1974574	CHEMBL3608450
CHEMBL1242977	CHEMBL1980178	CHEMBL1555396	CHEMBL1976499	CHEMBL123443	CHEMBL405317
CHEMBL1336469	CHEMBL1586726	CHEMBL3639695	CHEMBL1391256	CHEMBL504791	CHEMBL1498886
CHEMBL457504	CHEMBL86931	CHEMBL428496	CHEMBL1966279	CHEMBL45891	CHEMBL1562333
CHEMBL568385	CHEMBL3093311	CHEMBL1969735	CHEMBL1078178	CHEMBL1982383	CHEMBL1328369
CHEMBL3660186	CHEMBL600	CHEMBL1984842	CHEMBL471043	CHEMBL1802360	CHEMBL3586577
CHEMBL1084969	CHEMBL3976721	CHEMBL1554	CHEMBL2387265	CHEMBL225071	
CHEMBL80	CHEMBL600336	CHEMBL276140	CHEMBL313842	CHEMBL1983449	
CHEMBL2385591	CHEMBL1230020	CHEMBL3969665	CHEMBL481245	CHEMBL3800138	
CHEMBL1288582	CHEMBL317956	CHEMBL1971947	CHEMBL315538	CHEMBL3408737	

**Combinatory term queries**   **(UniProt protein acc. | DrugBank drug id | ChEMBL compound id | Pathway name | Disease name | HPO name)**
Q96FG2 | DB04400 | CHEMBL281211 | Tyrosine metabolism | Renal-hepatic-pancreatic dysplasia | Proximal lower limb amyotrophy
Q86XI8 | DB08150 | CHEMBL2205832 | D-Glutamine and D-glutamate metabolism | Congenital myasthenic syndromes with glycosylation defect | Alveolar rhabdomyosarcoma
Q6IAA8 | DB08149 | CHEMBL2004417 | One carbon pool by folate | Juvenile polyposis of infancy | Hyperhomocystinemia
Q9H9Q4 | DB05688 | CHEMBL261387 | alpha-Linolenic acid metabolism | focal segmental glomerulosclerosis | Impacted tooth
Q92794 | DB03144 | CHEMBL1365701 | One carbon pool by folate | Wilson-Turner syndrome | White hair
Q9BYE7 | DB03977 | CHEMBL1602632 | Vitamin digestion and absorption | Lung Lymphangioleiomyomatosis | Generalized hypotonia due to defect at the neuromuscular junction
Q9UKW4 | DB00810 | CHEMBL2337697 | Pantothenate and CoA biosynthesis | Juvenile polyposis of infancy | Incomplete partition of the cochlea
P05112 | DB00772 | CHEMBL1452003 | Proximal tubule bicarbonate reclamation | Achondrogenesis | Elevated plasma branched chain amino acids
O75603 | DB03632 | CHEMBL3883820 | Nitrogen metabolism | Monilethrix | Chronic tubulointerstitial nephritis
Q6E0U4 | DB07707 | CHEMBL3098606 | Renin-angiotensin system | Phosphoenolpyruvate carboxykinase deficiency | Testicular dysgenesis
Q86X02 | DB12228 | CHEMBL572163 | Biotin metabolism | Bilateral striopallidodentate calcinosis | Respiratory paralysis
O43293 | DB08855 | CHEMBL1602926 | Autophagy | Pseudoxanthoma elasticum | Thickening of the glomerular basement membrane
P21964 | DB08248 | CHEMBL1370189 | DNA replication | Polyglucosan body myopathy | Type II transferrin isoform profile
O43918 | DB01520 | CHEMBL326962 | DNA replication | cirrhosis of liver | Aplasia of the ovary
Q9Y239 | DB01036 | CHEMBL2011154 | Aldosterone-regulated sodium reabsorption | Multiple osteochondromas | Abnormal pelvis bone morphology
O14654 | DB11275 | CHEMBL3590081 | Glycosphingolipid biosynthesis | Duane retraction syndrome | Hepatic cysts
Q92614 | DB04707 | CHEMBL1471698 | Glycosylphosphatidylinositol (GPI)-anchor biosynthesis | hyperthyroxinemia | Macular hypoplasia
Q9UHJ3 | DB08371 | CHEMBL317956 | Butanoate metabolism | Intermediate maple syrup urine disease | Streak ovary
Q14088 | DB07795 | CHEMBL1304004 | Glycosphingolipid biosynthesis | Hypotonia - cystinuria syndrome | Low back pain
Q8NFG4 | DB09409 | CHEMBL4083333 | Glycosaminoglycan degradation | Perrault syndrome | Tibial torsion
Q9H078 | DB04930 | CHEMBL1324434 | Glycosphingolipid biosynthesis | tibia, hypoplasia or aplasia of, with polydactyly | Hepatic necrosis
Q9Y561 | DB00890 | CHEMBL1491933 | Phenylalanine, tyrosine and tryptophan biosynthesis | Jackson-Weiss syndrome | Agenesis of lateral incisor
Q9Y6D6 | DB06713 | CHEMBL1597827 | Valine, leucine and isoleucine biosynthesis | Idiopathic ventricular fibrillation, not Brugada type | Right-to-left shunt
Q02410 | DB03948 | CHEMBL1509338 | SNARE interactions in vesicular transport | Myoclonus-dystonia syndrome | Lingual dystonia
Q99593 | DB02835 | CHEMBL4072879 | Pentose and glucuronate interconversions | Ehlers-Danlos syndrome, periodontitis type | Elevated creatine kinase after exercise
Q96GT9 | DB00245 | CHEMBL1586534 | Selenocompound metabolism | Autosomal recessive progressive external ophthalmoplegia | Abnormality of neck blood vessel

Q13813 | DB08808 | CHEMBL489738 | Starch and sucrose metabolism | Asperger syndrome | Long foot
Q99819 | DB00651 | CHEMBL2205362 | Renin-angiotensin system | Krabbe disease | Hyperlysinuria
P07711 | DB00892 | CHEMBL536950 | Renin-angiotensin system | obsolete_polycystic lipomembranous osteodysplasia with sclerosing leukoencephalopathy | Abnormality of the Eustachian tube
O14497 | DB07636 | CHEMBL1172251 | Riboflavin metabolism | Muscle-eye-brain disease | Misalignment of incisors
P02686 | DB09068 | CHEMBL2441342 | Mannose type O-glycan biosynthesis | alcohol dependence | Periventricular heterotopia
Q9NX58 | DB03109 | CHEMBL1331514 | Glycosphingolipid biosynthesis | Hyperostosis corticalis generalisata | Adenocarcinoma of the colon
O60936 | DB00798 | CHEMBL2425959 | Other glycan degradation | Non-acquired isolated growth hormone deficiency | Morning myoclonic jerks
Q9NV29 | DB00451 | CHEMBL272401 | Phenylalanine metabolism | Dehydrated hereditary stomatocytosis | Colonic diverticula
P50553 | DB01154 | CHEMBL258405 | Renin-angiotensin system | habitual abortion | Impaired visuospatial constructive cognition
P23297 | DB00584 | CHEMBL1241775 | Linoleic acid metabolism | Subcortical band heterotopia | Multiple impacted teeth
Q13951 | DB00262 | CHEMBL1076478 | Propanoate metabolism | Hypocalcified amelogenesis imperfecta | Pulmonary cyst
Q13144 | DB04566 | CHEMBL241115 | Arginine biosynthesis | Chronic mucocutaneous candidosis | Cavum septum pellucidum
O43707 | DB06271 | CHEMBL3945264 | Citrate cycle (TCA cycle) | persistent fetal circulation syndrome | Pancreatic hypoplasia
Q969H8 | DB09059 | CHEMBL1389047 | Fat digestion and absorption | Autosomal recessive cerebellar ataxia | Fatigable weakness of distal limb muscles
P20774 | DB07715 | CHEMBL3600698 | Glycosphingolipid biosynthesis | hepatocellular carcinoma | Retinal arteriolar tortuosity
P20783 | DB01120 | CHEMBL243712 | RNA polymerase | Hereditary chronic pancreatitis | Narcolepsy
Q8TAC9 | DB00498 | CHEMBL177820 | Folate biosynthesis | cataract | Abnormal size of pituitary gland
P09681 | DB06822 | CHEMBL1995948 | Primary bile acid biosynthesis | osteoporosis | Optic neuritis
O75445 | DB00884 | CHEMBL143210 | Butanoate metabolism | type II hypersensitivity reaction disease | Lack of insight
Q9BRP4 | DB00422 | CHEMBL4293085 | Phenylalanine metabolism | Cornelia de Lange syndrome | Entrapment neuropathy
Q5T5X7 | DB05149 | CHEMBL164747 | Glycosphingolipid biosynthesis | Barrett's esophagus | Sinus bradycardia
Q00975 | DB04217 | CHEMBL1516500 | Other glycan degradation | Autosomal agammaglobulinemia | Abnormality of the urachus
O94955 | DB01011 | CHEMBL1472703 | Selenocompound metabolism | Immunodeficiency due to a complement cascade protein anomaly | Moyamoya phenomenon
P20809 | DB05078 | CHEMBL1515004 | Folate biosynthesis | Hereditary spherocytosis | Hyperechogenic kidneys
Q9HBW0 | DB04223 | CHEMBL3401988 | Detoxification of Reactive Oxygen Species | Multiple pterygium syndrome | Abnormality of renin-angiotensin system
O60673 | DB00117 | CHEMBL1369262 | Signal regulatory protein family interactions | Prader-Willi syndrome | Vertebral wedging
Q8WUH2 | DB00716 | CHEMBL3628807 | Initiation of Nuclear Envelope Reformation | Aniridia | EEG with polyspike wave complexes
Q14802 | DB01013 | CHEMBL1526414 | Signaling by ERBB2 | Sideroblastic anemia | Head titubation
Q9H6W3 | DB09099 | CHEMBL2012686 | Negative regulation of FGFR3 signaling | Frontotemporal dementia and amyotrophic lateral sclerosis | Supernumerary vertebrae
P62508 | DB13151 | CHEMBL468167 | Muscarinic acetylcholine receptors | Melanoma | Abnormality of the protein C anticoagulant pathway
Q8IYB3 | DB01247 | CHEMBL3110005 | Passive transport by Aquaporins | Cerebral cavernous malformation | True hermaphroditism
Q8TEX9 | DB12334 | CHEMBL1529320 | ATF6 (ATF6-alpha) activates chaperones | Infantile hemangioma | Neonatal insulin-dependent diabetes mellitus
Q08ER8 | DB04327 | CHEMBL407734 | Truncations of AMER1 destabilize the destruction complex | Essential tremor | Civatte bodies
Q7KZI7 | DB00574 | CHEMBL1385673 | Choline catabolism | Familial amyloidosis | Cardiac shunt
Q13569 | DB02900 | CHEMBL2007375 | Removal of the Flap Intermediate | ATP synthase deficiency | Abnormal brain lactate level by MRS
P18074 | DB13944 | CHEMBL559015 | STING mediated induction of host immune responses | MELAS Syndrome | Femoral hernia
O60732 | DB00876 | CHEMBL3196267 | Scavenging by Class F Receptors | Vulvar cancer | Thyroid hyperplasia
Q5VSL9 | DB04299 | CHEMBL118919 | Synthesis of IP2, IP, and Ins in the cytosol | Behcet disease | Reduced antithrombin III activity
P13674 | DB09073 | CHEMBL4295242 | CD209 (DC-SIGN) signaling | Hypomagnesemia | Male sexual dysfunction
Q9H270 | DB03536 | CHEMBL1306267 | PKA-mediated phosphorylation of CREB | Capillary malformation-arteriovenous malformation | Proximal symphalangism

Q9Y6Y9 | DB03501 | CHEMBL3355683 | POU5F1 (OCT4), SOX2, NANOG repress genes related to differentiation | Malignant pleural mesothelioma | Abnormal muscle fiber protein expression
P28288 | DB00579 | CHEMBL533602 | TGF-beta receptor signaling in EMT (epithelial to mesenchymal transition) | Congenital diarrhea | Hypoplastic scapulae
P61158 | DB08299 | CHEMBL1329507 | mRNA Editing: C to U Conversion | Tetralogy of Fallot | Facial hirsutism
P09565 | DB00335 | CHEMBL318018 | ERBB2 Activates PTK6 Signaling | Spondyloepimetaphyseal dysplasia | Chronic hepatitis due to cryptosporidium infection
Q8NE63 | DB00449 | CHEMBL192293 | Trafficking of myristoylated proteins to the cilium | Griscelli syndrome | Absent axillary hair
Q8N2N9 | DB01954 | CHEMBL252403 | rRNA modification in the mitochondrion | Mantle cell lymphoma | Hyperchloremia
P15692 | DB02032 | CHEMBL1668605 | Ion channel transport | Syndromic craniosynostoses | Hypereosinophilia
P00414 | DB04325 | CHEMBL1474625 | MECP2 regulates transcription factors | X-linked lymphoproliferative syndrome | Vestibular areflexia
Q8TBB5 | DB01821 | CHEMBL2313377 | Transcriptional regulation by the AP-2 (TFAP2) family of transcription factors | Ectopia lentis | Testicular dysgenesis
O75971 | DB05260 | CHEMBL2323582 | Metallothioneins bind metals | Dent disease | Carotid artery stenosis
P31321 | DB00222 | CHEMBL2443204 | Scavenging by Class H Receptors | Distal arthrogryposis | Prolonged PR interval
P36402 | DB02559 | CHEMBL219586 | Glucocorticoid biosynthesis | Merkel cell carcinoma | Prominent digit pad
P11684 | DB08271 | CHEMBL491771 | Regulation of APC/C activators between G1/S and early anaphase | Saethre-Chotzen syndrome | Aplasia/Hypoplasia of the distal phalanges of the toes
Q8NFA0 | DB08234 | CHEMBL80155 | Regulation of signaling by CBL | Ehlers-Danlos syndrome kyphoscoliosis type | Aplasia/Hypoplasia of the tragus
Q9BW83 | DB00883 | CHEMBL295806 | RAF-independent MAPK1/3 activation | Wiskott-Aldrich syndrome | Narrow vertebral interpedicular distance
Q9H3F6 | DB06021 | CHEMBL111589 | p75NTR negatively regulates cell cycle via SC1 | Renal hypouricemia | Gastrointestinal eosinophilia
Q6ZP80 | DB01288 | CHEMBL3356927 | Sodium-coupled sulphate, di- and tri-carboxylate transporters | Striate palmoplantar keratoderma | Partial atrioventricular canal defect
Q13698 | DB01842 | CHEMBL2007375 | PKA-mediated phosphorylation of CREB | Complement regulatory protein defects | Abnormal circulating serine family amino acid concentration
Q8NFD5 | DB09205 | CHEMBL1501724 | Transcription of E2F targets under negative control by p107 (RBL1) and p130 (RBL2) in complex with HDAC1 | Bare lymphocyte syndrome type2 | Thick nasal alae
Q12772 | DB00093 | CHEMBL1511350 | Interaction With The Zona Pellucida | Aicardi-Goutieres syndrome | Abnormal atrioventricular connection
P17181 | DB12095 | CHEMBL2385582 | Phosphorylation of CD3 and TCR zeta chains | Malignant paraganglioma | Hypolipidemia
Q8IYS1 | DB08149 | CHEMBL340211 | TRAF6 mediated IRF7 activation | Alpha-ketoglutarate dehydrogenase complex deficiency | Sparse lower eyelashes
O43143 | DB07557 | CHEMBL1395196 | Termination of O-glycan biosynthesis | Chronic lymphocytic leukemia | Full-thickness macular hole
P22001 | DB02068 | CHEMBL2387068 | Competing endogenous RNAs (ceRNAs) regulate PTEN translation | Stickler syndrome | Reduced factor VIII activity
P10145 | DB01863 | CHEMBL1463349 | CYP2E1 reactions | Primary hyperparathyroidism | Abnormality of the endometrium
Q9C0J9 | DB07950 | CHEMBL3935597 | The canonical retinoid cycle in rods (twilight vision) | Tyrosinemia | Chronic mucocutaneous candidiasis
P08571 | DB00685 | CHEMBL3912801 | eNOS activation | Primary hyperoxaluria | Bladder diverticulum
P36578 | DB05266 | CHEMBL1256655 | Gap-filling DNA repair synthesis and ligation in GG-NER | Hermansky-Pudlak syndrome | Platelet anisocytosis
P05113 | DB00135 | CHEMBL203637 | Regulation of insulin secretion | Mitochondrial trifunctional protein deficiency | No social interaction
Q03518 | DB07771 | CHEMBL196598 | TFAP2 (AP-2) family regulates transcription of cell cycle factors | Mosaic variegated aneuploidy syndrome | Ectropion of lower eyelids
O75132 | DB03904 | CHEMBL1630940 | Creatine metabolism | SEMD with joint laxity type | Y-linked inheritance
Q86UD4 | DB02180 | CHEMBL4063105 | TWIK related potassium channel (TREK) | Short rib-polydactyly syndrome | Marked muscular hypertrophy
Q9BW62 | DB00083 | CHEMBL1303 | CREB phosphorylation | Frontotemporal dementia and amyotrophic lateral sclerosis | Excessive daytime sleepiness
Q01658 | DB05037 | CHEMBL1363706 | Transport of gamma-carboxylated protein precursors from the endoplasmic reticulum to the Golgi apparatus | Brachydactyly | Morphological abnormality of the semicircular canal

Table S9. Statistics regarding all frequently observed terms in 1365 CROssBAR knowledge graphs constructed with random term queries.

Biological component type	Term id	Term name	Count (g)	Observed frequency (g/G)	Mean observed frequency of the component
Gene/Protein (Core)	P02545	LMNA	78	0.0571	0.0025
	P10636	MAPT	58	0.0425	0.0025
	P51151	RAB9A	49	0.0359	0.0025
	P05177	CYP1A2	45	0.0330	0.0025
	Q16637	SMN1	45	0.0330	0.0025
Gene/Protein (Neighboring)	Q8TDT2	GPR152	74	0.0542	0.0023
	O76011	KRT34	52	0.0381	0.0023
	Q8NBJ4	GOLM1	45	0.0330	0.0023
	Q9Y320	TMX2	42	0.0308	0.0023
	Q9NUH8	TMEM14B	37	0.0271	0.0023
Reactome Pathway	R-HSA-418594	G alpha (i) signalling events	49	0.0359	0.0055
	R-HSA-9027307	Biosynthesis of maresin-like SPMs	49	0.0359	0.0055
	R-HSA-390696	Adrenoceptors	46	0.0337	0.0055
	R-HSA-390651	Dopamine receptors	44	0.0322	0.0055
	R-HSA-352238	Breakdown of the nuclear lamina	43	0.0315	0.0055
KEGG Pathway	hsa01100	Metabolic pathways	310	0.2271	0.0349
	hsa04080	Neuroactive ligand-receptor interaction	168	0.1231	0.0349
	hsa04151	PI3K-Akt signaling pathway	137	0.1004	0.0349
	hsa04010	MAPK signaling pathway	125	0.0916	0.0349
	hsa04510	Focal adhesion	113	0.0828	0.0349
EFO Disease	Orphanet:300751	Familial dilated cardiomyopathy with conduction defect due to LMNA mutation	40	0.0293	0.0029
	Orphanet:99750	Atypical progressive supranuclear palsy	30	0.0220	0.0029
	EFO:0003829	alcohol dependence	30	0.0220	0.0029
	Orphanet:90153	Mandibuloacral dysplasia with type A lipodystrophy	29	0.0212	0.0029
	Orphanet:740	Hutchinson-Gilford progeria syndrome	29	0.0212	0.0029
KEGG Disease	H00601	Hutchinson-Gilford progeria syndrome	54	0.0396	0.0044
	H00606	Early infantile epileptic encephalopathy	50	0.0366	0.0044
	H01611	Alcohol dependence	49	0.0359	0.0044
	H01450	Obsessive-compulsive disorder	44	0.0322	0.0044
	H00077	Progressive supranuclear palsy	44	0.0322	0.0044
Phenotype (HPO)	HP:0002528	Granulovacuolar degeneration	25	0.0183	0.0019
	HP:0012166	Skin-picking	25	0.0183	0.0019
	HP:0031825	Freezing of gait	23	0.0168	0.0019
	HP:0031937	Tachylalia	23	0.0168	0.0019
	HP:0002439	Frontolimbic dementia	23	0.0168	0.0019
Drug	DB12010	Fostamatinib	60	0.0440	0.0023
	DB00715	Paroxetine	47	0.0344	0.0023
	DB00433	Prochlorperazine	47	0.0344	0.0023
	DB00243	Ranolazine	40	0.0293	0.0023
	DB00924	Cyclobenzaprine	38	0.0278	0.0023
Compound (Experimentally measured bioactivity)	CHEMBL1214871	CHEMBL1214871	15	0.0110	0.0010
	CHEMBL1762790	CHEMBL1762790	10	0.0073	0.0010
	CHEMBL3939307	CHEMBL3939307	10	0.0073	0.0010
	CHEMBL80919	CHEMBL80919	9	0.0066	0.0010
	CHEMBL338988	CHEMBL338988	9	0.0066	0.0010
Compound (Predicted)	CHEMBL551249	CHEMBL551249	20	0.0147	0.0013
	CHEMBL553548	CHEMBL553548	20	0.0147	0.0013
	CHEMBL1822108	CHEMBL1822108	20	0.0147	0.0013
	CHEMBL547077	CHEMBL547077	20	0.0147	0.0013
	CHEMBL551228	CHEMBL551228	19	0.0139	0.0013
